# Isoform-specific and ubiquitination dependent recruitment of Tet1 to replicating heterochromatin modulates methylcytosine oxidation

**DOI:** 10.1038/s41467-022-32799-8

**Published:** 2022-09-02

**Authors:** María Arroyo, Florian D. Hastert, Andreas Zhadan, Florian Schelter, Susanne Zimbelmann, Cathia Rausch, Anne K. Ludwig, Thomas Carell, M. Cristina Cardoso

**Affiliations:** 1 Cell Biology and Epigenetics, Department of Biology, Technical University of Darmstadt, Schnittspahnstr. 10, 64287 Darmstadt, Germany; 2 Section AIDS and newly emerging pathogens, Paul Ehrlich Institute, Paul-Ehrlich-Str. 51-59, 63225 Langen, Germany; 3 Department of Chemistry, Ludwig Maximilians University, Butenandstr. 5-13, 81377 Munich, Germany; 4 Present Address: Luxembourg Centre for Systems Biomedicine, University of Luxembourg, 6, avenue du Swing, L-4367 Belvaux, Luxembourg; 5 Present Address: Department of Medicine, Hematology, Oncology and Rheumatology, University Hospital Heidelberg, Im Neuenheimer Feld 410, 69120 Heidelberg, Germany

**Keywords:** Cellular imaging, Nuclear organization, Ubiquitylation, DNA methylation

## Abstract

Oxidation of the epigenetic DNA mark 5-methylcytosine by Tet dioxygenases is an established route to diversify the epigenetic information, modulate gene expression and overall cellular (patho-)physiology. Here, we demonstrate that Tet1 and its short isoform Tet1s exhibit distinct nuclear localization during DNA replication resulting in aberrant cytosine modification levels in human and mouse cells. We show that Tet1 is tethered away from heterochromatin via its zinc finger domain, which is missing in Tet1s allowing its targeting to these regions. We find that Tet1s interacts with and is ubiquitinated by CRL4(VprBP). The ubiquitinated Tet1s is then recognized by Uhrf1 and recruited to late replicating heterochromatin. This leads to spreading of 5-methylcytosine oxidation to heterochromatin regions, LINE 1 activation and chromatin decondensation. In summary, we elucidate a dual regulation mechanism of Tet1, contributing to the understanding of how epigenetic information can be diversified by spatio-temporal directed Tet1 catalytic activity.

## Abstract (web summary)

A short isoform of the Tet1 enzyme (Tet1s) that oxidizes the DNA 5-methylcytosine (5mC) mark is overexpressed in tumors. Here the authors show Tet1s, but not full length Tet1, changes localization over the cell cycle upon ubiquitination and Uhrf1 interaction and is targeted to heterochromatin during S-phase. This leads to 5mC oxidation and loss of DNA methylation in heterochromatin.

## Introduction

Covalent modifications of the fifth cytosine carbon atom in mammalian DNA play a crucial role in cellular homeostasis and faulty cytosine modification patterns are linked to a multitude of diseases, namely cancer^1^. Consequently, proteins responsible for these modifications or capable of interacting with them were implicated in essential, physiological and pathophysiological cellular processes^2^. The best-studied eukaryotic DNA modification is 5-methylcytosine (5mC), which correlates with transcriptional silencing. The levels of 5mC are maintained during DNA replication in the S-phase of the cell cycle by DNA methyltransferase 1 (Dnmt1), the founding member of the DNA methyltransferase family (Dnmt)^2,3^. During DNA replication in euchromatin and facultative heterochromatin, Dnmt1 associates with the replication machinery via its polymerase clamp PCNA binding domain (PBD)^4^. In late S-phase an important cofactor of Dnmt1 is the E3-ligase Uhrf1 which plays a crucial role in cellular homeostasis and maintenance of DNA methylation. Besides the E3-ligase activity mediated by its RING-domain, Uhrf1 harbors an Ubl domain; the PHD and TTD domains capable of binding different histone modifications; and the SRA domain which recognizes modified cytosine^5^. Cooperative binding of Uhrf1 to hemi-methylated CpG sites and trimethylated H3K9, as well as H3K18/K23 ubiquitination, directs Dnmt1 to sites of ongoing DNA replication in pericentric heterochromatin^6,7^, which shows high levels of 5mC around centromeric regions.

Methylated cytosine (5mC) can successively be oxidized to 5-hydroxymethylcytosine (5hmC), 5-formylcytosine (5fC) and 5-carboxylcytosine (5caC) by the members of the Ten-eleven translocation (Tet) protein family^2,8,9^. All three Tet protein family members, Tet1, Tet2 and Tet3, share the same catalytic activity and high sequence similarities in their C-terminal catalytic domains^10^. Despite this, major differences are observed in their respective expression levels throughout different tissues and developmental stages, and therefore their physiological roles. While Tet3 is predominantly expressed during early embryogenesis and also in post-mitotic neurons, Tet1 and Tet2 are found to be expressed more ubiquitously across different tissues and developmental stages from embryonic stem cells to somatic cells^11^.

A structural and functional feature that separates the three Tet proteins from one another is their N-terminal zinc finger domain. Tet2 lost its zinc finger during evolution through chromosomal inversion and this function is now taken over by the genomically adjacent Idax/CxxC4, which was found to negatively regulate Tet2 activity^12^. Tet1 and Tet3, in contrast, both kept their respective zinc fingers, and the Tet3 CxxC domain was shown to bind caC, thereby regulating neurodegeneration^13^. The Tet1 zinc finger domain, on the other hand, was found to mostly bind non-modified DNA^14^ and was implicated in preventing DNA methylation spreading in euchromatic regions, but it is unclear how this is regulated^15^. While three different Tet3 isoforms have been characterized to date^13^, a N-terminally truncated Tet1 isoform (Tet1s), which lacks the zinc finger domain, was discovered recently and attributed a role in reproduction control and embryogenesis, but also in cancerogenesis^16–18^. In the latter, a strong increase of Tet1s activity at non-CpG islands was observed together with transcriptional activation, while genic regions were mostly targeted by full-length Tet1 via its CxxC domain^17^. However, how Tet1 and its short isoform are differentially regulated remains elusive.

Besides the double-stranded beta helix (DSBH) domain, which harbors the Fe(II) and 2-oxoglutarate cofactor binding sites, the C-terminal catalytic core of all three Tet proteins also comprises the cysteine-rich domain (CRD). Two studies identified monoubiquitination by the CRL4(VprBP) E3-ligase complex of a conserved lysine residue within the CRD of all three Tet proteins to be essential for their catalytic activity, and a lysine to glutamate mutation was shown to abrogate catalytic activity^19,20^.

As the two Tet1 isoforms were shown to target different genomic regions, we hypothesized that they could have different subcellular distributions, which in turn could be regulated during the cell cycle. Hence, we aimed to elucidate the subcellular localization of the Tet proteins and the mechanism regulating it. Furthermore, we investigated the effect of the Tet1 isoforms on 5mC oxidation in euchromatic and heterochromatic regions, and the resulting biological consequences like repetitive DNA element activation and chromatin decompaction. This study shows that the short isoform of Tet1 but not the full-length Tet1 isoform localizes during S phase to sites of ongoing DNA replication in heterochromatin in an Uhrf1- and CRL4(VprBP) dependent manner, by ubiquitination of the conserved lysine residue in the CRD of Tet1s. This results in a significant de novo 5hmC formation, globally, and more so in heterochromatin, including LINE 1 interspersed DNA repeats leading to their activation. In addition, we report Tet1 localization to be prevented by the N-terminal zinc finger domain of full-length Tet1 by a passive mechanism that is based on retention of Tet1 in euchromatin by non-sequence specific chromatin binding. Taken together, we delineate a dual mechanism that regulates the subnuclear localization of Tet1 and its short isoform and consequently their catalytic activity.

## Results

### The short isoform of Tet1 is recruited to heterochromatin during ongoing DNA replication in S-phase and increases 5mC oxidation

**Fig. 1:**
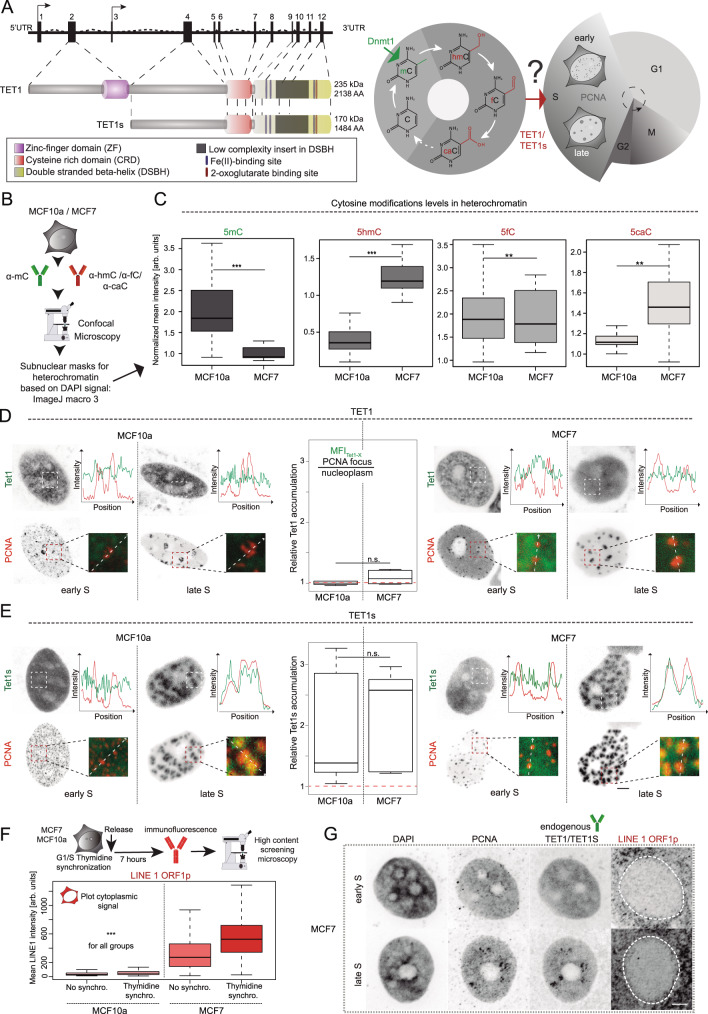
Breast cancer cells show aberrant 5mC oxidation and increased LINE 1 ORF1p level. **A** Gene structure of the human *TET1* locus. Non-coding exons 1 and 3 harbor different transcription start sites, targeted by a different promoter. Translation starts in exon 2 (TET1) and 4 (TET1s). TET1 protein functional domains are indicated. Graphical representation of cytosine modifications and their maintenance during the cell cycle. **B** Fiji-based in situ cytosine modifications analysis procedure. MCF cells were immunostained and imaged using confocal microscopy. Binary nuclear and heterochromatin masks were generated based on DNA signals (DAPI). Mean fluorescence intensities in the respective areas were measured. **C** Boxplots showing normalized mean intensity of cytosine modifications at heterochromatin regions. *n* (5mC) = 23 (MCF10a) - 17 (MCF7), *n* (5hmC) = 19 (MCF10a) - 20 (MCF7), *n* (5fC) = 21 (MCF10a) - 17 (MCF7), *n* (5caC) = 17 (MCF10a) - 16 (MCF7) cells. **D**, **E** Live-cell analysis of Tet1 and Tet1s subnuclear localization: confocal images of MCF10a and MCF7 cells expressing EGFP-Tet1s/EGFP-Tet1 and mRFP-PCNA 8 h post-transfection. Colocalization of Tet1-X with PCNA was examined by line-profile analysis and relative protein accumulation in early and late S-phase. *n* (TET1) = 14 (MCF10a) - 13 (MCF7), *n* (Tet1s) = 16 (MCF10a) - 17 (MCF7) cells. **F** Synchronized MCF cells immunostained against LINE 1 ORF1p. Cytoplasmic fluorescence mean intensity levels were plotted. Representative confocal images for these immunostainings, including endogenous TET1/TET1s, are shown in **G**. For all boxplots, the box represents 50% of the data, starting in the first quartile (25%) and ending in the third (75%). The line inside represents the median. The whiskers represent the upper and lower quartile. Statistical significance was tested with a paired two-samples Wilcoxon test (n.s. not significant, is given for *p*-values ≥ 0.05; one star (*) for *p*-values < 0.05 and ≥ 0.005; two stars (**) is given for values < 0.005 and ≥ 0.0005; three stars (***) is given for values < 0.0005). N-numbers and *p*-values are shown in Supplementary Data 1. Source data are provided as a Source Data file. Scale bars = 5 µm.

A hallmark of many cancers are aberrant DNA cytosine modification levels and, in particular, global hypomethylation concomitantly with local hypermethylation. Hypermethylation is often found in promoters and coding regions of tumor suppressor genes, which are both usually hypomethylated in normal tissues^21^. Interestingly, also the canonical Tet1 promoter was found to become hypermethylated in many cancers^22^, resulting in the use of an alternative promoter as well as an alternative transcription start site. This consequently leads to the expression of a N-terminally truncated, but catalytically active, short isoform Tet1s (Fig. 1A)^17^.

Based on these findings, we first addressed differences in TET1 isoforms and cytosine modification levels between MCF7 human breast adenocarcinoma cells and MCF10a non-transformed human breast epithelial cells. While MCF7 cells expressed high levels of both TET1 isoforms, MCF10a cells showed comparatively low levels. However, both MCF cell lines expressed similar levels of TET2, and very low levels of TET3^17^ (Supplementary Fig. 1A). To address the cytosine modification levels in these cell lines, cells were immunostained with antibodies against 5mC and 5hmC, 5fC or 5caC (Fig. 1B and Supplementary Fig. 1B). Levels of 5hmC, 5fC or 5caC were measured and normalized against the respective normalized 5mC levels to compensate for the epigenetic heterogeneity in cancer cells^23^. As expected, MCF7 cells showed significantly lower levels of 5mC in heterochromatin, compared to MCF10a cells. Normalized 5mC levels in pericentric heterochromatin regions of MCF7 cells were reduced by almost 50% in comparison to MCF10a cells, but the strong loss of 5mC is also underlined by the ratio of 5hmC to 5mC levels in pericentric heterochromatin. The 5hmC/5mC ratio is significantly increased in MCF7 cells compared to MCF10a cells, which is mostly due to the substantial 5mC decrease. In contrast, levels of 5hmC, not so much 5fC, but especially 5caC were elevated in MCF7 cells (Fig. 1C), which is in line with previous findings in breast cancer^24^. Supplementary Fig. 1C shows representative images of cytosine modifications immunostaining in these cell lines. These results were reproduced using a different immunostaining protocol for detecting DNA modifications (Supplementary Fig. 1D).

The levels of the different oxidative 5mC derivatives were previously found to peak at the end of S-phase similar to 5mC^25,26^ and in addition, Tet1 has been implicated as a maintenance DNA demethylase that prevents aberrant methylation spreading^15^. These findings and our just described observations prompted us to investigate the subnuclear localization of Tet1 and its short isoform, particularly during ongoing DNA replication in S-phase. Due to the different expression levels of Tet1 and Tet1s in the two cell types and to avoid fixation artifacts, we made use of live-cell microscopy of fluorescently tagged variants of Tet1 as well as PCNA (proliferating cell nuclear antigen) as a marker for S-phase substages. PCNA is the DNA polymerase processivity clamp and shows distinct subnuclear patterns during the replication of different chromatin domains. While replication of euchromatic domains is characterized by many small, homogeneously distributed subnuclear puncta, larger PCNA clusters are observed during heterochromatin replication^27,28^. We, therefore, compared cells with PCNA patterns that could clearly be assigned to DNA replication of euchromatin or heterochromatin domains, hence to early and late S-phase, respectively. In doing so, we found that neither full-length Tet1 nor Tet1s showed recruitment during euchromatin replication in either cell type (Fig. 1D, E). The accumulation of TET1 or TET1s in replicating heterochromatin was quantified by measuring the respective TET1-X levels and dividing it by the levels in the nucleoplasm. Furthermore, TET1 did not show any noteworthy accumulations in sites of ongoing DNA replication in heterochromatin. Of note, TET1s showed a clear overlap with PCNA at late S-phase sites as verified by line-profile colocalization analyses (Fig. 1E), indicating a recruitment during the replication of 5mC-rich heterochromatin.

### Tet1s oxidation of heterochromatin increases LINE 1 DNA repeat activation

Next, we tested the impact of TET1s recruitment to replicating heterochromatin regions and the consequences of 5hmC increase in these genomic loci. Euchromatin and heterochromatin are differentially populated by interspersed repetitive DNA sequences^29–31^. Alu repetitive DNA elements are among the most abundant SINEs (short interspersed nuclear elements). Alu are located mostly in gene-rich euchromatin, whereas retrotransposon-related long elements (LINEs) are located mostly in heterochromatic regions^32–34^. They are preferentially found at AT-rich and gene poor regions, corresponding to G-bands and DAPI-bright bands of metaphase chromosomes. Here, we focused on the LINE 1 (L1) element, and in particular, one of the products of its transcription and translation, the ORF1 protein (ORF1p)^35,36^. We aimed to determine whether TET1s-mediated 5hmC formation at heterochromatic regions leads to reactivation of LINE 1^37^. For this, we analyzed the levels of TET1s and LINE 1 ORF1p by immunofluorescence using high-content microscopy. Briefly, MCF10a and MCF7 cells were synchronized and fixed at late S phase. Then, cells were immunostained for LINE 1 ORF1p and compared with non-synchronized cells. While in MCF7 cells higher expression of TET1/TET1s corresponded to a significant increase in LINE 1 ORF1p levels, they were very low for MCF10a cells (Fig. 1F). Accordingly, LINE 1 ORF1p formation was increased in MCF7 cells and was even higher in S phase synchronized cells. Nonetheless, MCF10a cells showed lower ORF1p levels independently of the cell cycle stage. Representative images from confocal microscopy are shown in Fig. 1G and Supplementary Fig. 1E, where a cytoplasmic distribution for LINE 1 ORF1p is observed. ORF1 proteins bind to their own RNA in the cytosol to form a ribonucleoprotein particle (RNP), which facilitates the re-import of LINE 1 RNA to the nucleus^36^. For this reason, cytoplasmic levels were used in our analysis. Additionally, representative images showing the nuclear distribution of endogenous TET1/TET1s in MCF7 cells are shown in Fig. 1G. TET1/TET1s accumulation and colocalization with PCNA was visible during late S phase in dense DAPI regions, while MCF10a cells showed very low levels of endogenous TET1/TET1s proteins (Supplementary Fig. 1E). In addition, using immunofluorescence staining and high-content microscopy we verified that the levels of endogenous TET1/TET1s did not change more than 0.24% during the cell cycle (Supplementary Fig. 1F). This indicates that the biological effects of TET1s on 5mC oxidation and LINE 1 activation are not a reflection of a protein level change throughout the cell cycle but are a reflection of its subnuclear recruitment to heterochromatin during late S phase.

### Tet1 heterochromatin association maps to the catalytic domain

To investigate the differences in the subnuclear S-phase dynamics of Tet proteins in more detail, we selected C2C12 mouse myoblasts as model system, as their S-phase behavior and substage replication patterns are clearly distinguishable and have been extensively studied^38,39^. Also importantly, C2C12 cells, in their undifferentiated state, show low levels of all three Tet proteins^40^ as well as Mbd proteins^41^, which we have previously shown to counteract Tet catalytic activity^42–44^.

**Fig. 2:**
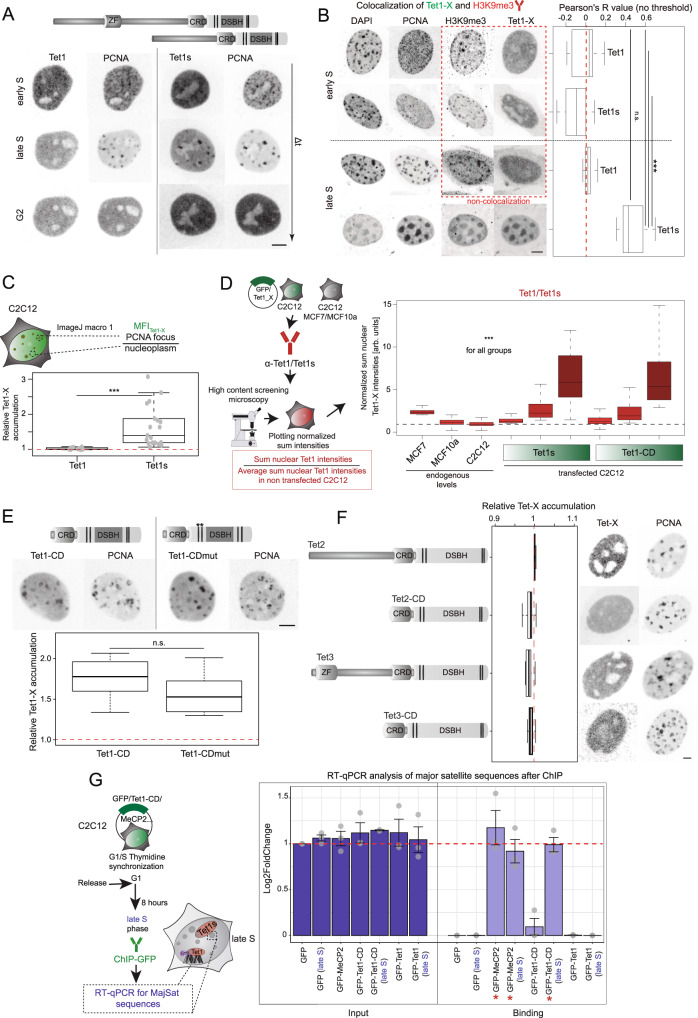
Tet1s localizes at heterochromatin during DNA replication. **A** Live-cell microscopy images of C2C12 cells expressing mRFP-PCNA as DNA replication marker and EGFP-Tet1-X. Cells showing an early S-phase pattern were imaged every 20 min. Time points in early, late S and G2 phases are shown. **B** Immunostaining against PCNA, H3K9me3 (as heterochromatin marker) and Tet1-X. C2C12 cells were transfected, immunostained, and imaged in S-phase. Pearson coefficient values (colocalization) were plotted for early versus late S-phase cells. **C** Tet1-X accumulation analysis in late S-phase cells. Mean fluorescence intensities of three nuclear areas inside and outside of DNA replication sites were measured and averaged. Mean fluorescence intensities of Tet1-X in PCNA foci were divided by the mean fluorescence in nucleoplasmic regions. Boxplot depicts the results of quantification. **D** Immunostaining against Tet1/Tet1s in MCF10a, MCF7 and C2C12 cells. Sum nuclear levels of Tet1/Tet1s and mean nuclear levels of EGFP were measured by wide-field high-content microscopy. Transfected C2C12 cells were grouped by their mean EGFP fluorescence (low, mid, high) (See Supplementary Fig. 2B, C). Boxplot shows Sum nuclear Tet1/Tet1s normalized by the average sum nuclear intensity in non-transfected C2C12. **E** Representative images of transfected C2C12 cells (mRFP-PCNA and EGFP-Tet1-CD/Tet1-CDmut). Boxplot shows Tet1-X relative accumulation. **F** Late S-phase C2C12 cells transfected with mRFP-PCNA and EGFP-Tet2/Tet3 proteins or respective catalytic domains. Boxplots show relative accumulation of Tet-X at replicating heterochromatin. **G** Chromatin immunoprecipitation (ChIP) followed by qPCR for MajSat sequences. C2C12 cells were transfected with Tet1-X and MeCP2 (positive control) and synchronized in G1/late S-phase. Barplots show the average value of amplification levels in input and chromatin binding fractions normalized to the GFP-input (red line). The whiskers represent the standard deviation with a 95% confidence interval. For all boxplots, the box represents 50% of the data, starting in the first quartile (25%) and ending in the third (75%). The line inside represents the median. The whiskers represent the upper and lower quartile. Statistical significance was tested with a paired two-samples Wilcoxon test (n.s., not significant, is given for *p*-values ≥ 0.05; one star (*) for *p*-values < 0.05 and ≥ 0.005; two stars (**) is given for values < 0.005 and ≥ 0.0005; three stars (***) is given for values < 0.0005). N-numbers and *p*-values are shown in Supplementary Data 1. Source data are provided as a Source Data file. Scale bar = 5 μm.

To avoid secondary effects from prolonged Tet overexpression and concomitant 5mC oxidation, cells were subjected to live-cell time lapse microscopy 8 h post-transfection. Live-cell imaging was initially chosen for protein accumulation analysis, as the observed localization of Tet1s was partially lost upon fixation (Supplementary Fig. 2A). Ectopically overexpressing Tet1 or Tet1s together with PCNA as a marker for S-phase progression, we found Tet1s, but not Tet1, to associate with sites of ongoing DNA replication in pericentric heterochromatin during late S-phase analogous to our results in human cells (Fig. 2A). Moreover, the observed recruitment of Tet1s was exclusively found in this substage of S-phase, hence, no accumulation during the replication of euchromatin in early S-phase or in G2 were observed (Fig. 2A, Supplementary Movies 1, 2). The accumulation of Tet1s was also measured using a marker for heterochromatic regions, H3K9me3^45^, together with PCNA. C2C12 cells were transfected with Tet1s or Tet1 and immunostained against H3K9me3 and PCNA. Confocal microscopy Z-stacks were taken of cells in early and late S phase and then analyzed for colocalization between Tet1-X and H3K9me3 using Fiji (Coloc2 plugging). After the analysis, Pearson coefficient values were obtained, with Tet1s showing values around 0.5 during late S phase, values close to 0 (no correlation) during early S phase, and Tet1 showing values around 0 or negative (anti-correlation) in both early or late S phase (Fig. 2B). Additionally, the accumulation of Tet1 or Tet1s in PCNA marked heterochromatin was quantified as described for MCF cells (Fig. 2C). To verify the biological significance of C2C12 cells as a model system, we measured Tet1 levels in MCF7, MCF10a, C2C12 and C2C12 cells overexpressing different Tet1 constructs (Tet1-X). To this end, cells were immunostained against Tet1 and the respective sum nuclear Tet1 intensities normalized to the average sum intensity in non-transfected C2C12 cells (Fig. 2D and Supplementary Fig. 2B). We validated that the anti-Tet1 antibody used reacts with both mouse and human proteins equally (Supplementary Fig. 2E). Transfected C2C12 cells were grouped according to their GFP or mcherry levels and plotted as low, mid and high overexpressing groups (Supplementary Fig. 2B, C). Non-transfected C2C12 cells showed the lowest Tet1 levels, while transfected C2C12 cells from the high overexpressing group showed the highest level. Tet1-X mid-overexpressing cells (group selected for most of the analyses) showed levels similar to MCF7 cells (Fig. 2D and Supplementary Fig. 2D), indicating that the selected ectopic expression levels in C2C12 cells emulate the endogenous Tet1 level in these cells. Our results furthermore imply that C2C12 cells are de facto phenotypically Tet1 negative, while low overexpression levels correspond to the non-tumor human cell line MCF10a.

In previous reports, it was demonstrated that the C-terminal catalytic domains of all three Tet family members are sufficient for their catalytic activity^9^ and that they share high sequence similarities^10^. Hence, besides the full-length proteins, we also investigated the subnuclear localization of the respective catalytic domains (TetX-CD) during the replication of heterochromatin by live-cell microscopy. In doing so, we found that neither Tet2 nor Tet3 nor their respective catalytic domains showed any noteworthy accumulations in late S-phase (Fig. 2F), whereas Tet1-CD showed a strong accumulation at replicating pericentric heterochromatin in late S-phase (Fig. 2E). In order to verify the observed Tet1-CD accumulation by other methods, chromatin immunoprecipitation experiments (ChIP) followed by qPCR of major satellite repeats (MajSat) were performed. GFP-Tet1-CD binding to these genomic regions was immunoprecipitated using the GBP beads. GFP and GFP-MeCP2 (methyl-CpG binding protein 2)^46^ were used as negative and positive controls, respectively, and GFP-input fraction was used for normalization in all samples. qPCR using primers for MajSat showed amplification of these sequences for Tet1-CD pulldown in cells synchronized in late S phase in a similar level to MeCP2, while no amplification was found for GFP and Tet1. Interestingly, MeCP2 binding fraction at late S phase showed slightly lower levels of amplification for MajSat repeats, corresponding to a slight displacement of this protein during replication (Fig. 2G)^47^. Similar levels of amplification were found for the input fraction in all samples.

**Fig. 3:**
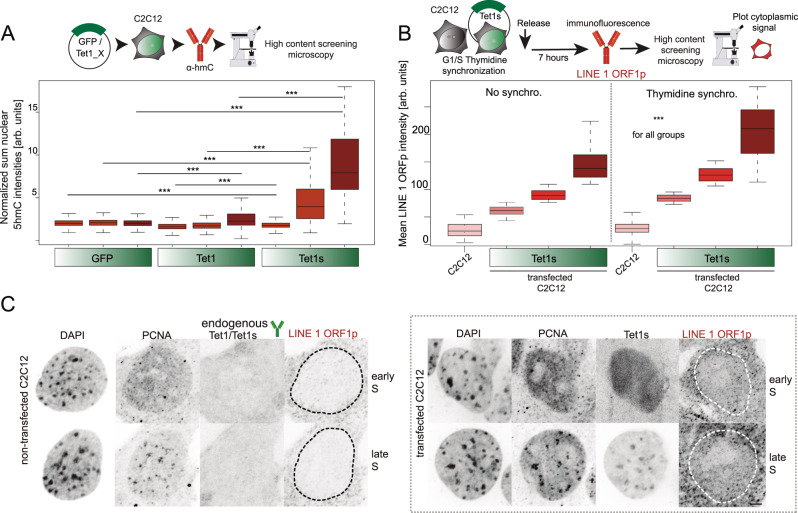
C2C12 cells overexpressing Tet1s show aberrant 5mC oxidation and higher levels of LINE 1 ORF1p. **A** Cells transfected with EGFP-Tet1, EGFP-Tet1s or EGFP alone, were immunostained against 5hmC. Fluorescence intensity levels of overexpressed proteins and 5hmC were measured, sum nuclear 5hmC levels were normalized to the sum nuclear DAPI intensity and grouped as described in Supplementary Fig. 2B, D. **B** C2C12 cells non-transfected and transfected with GFP-Tet1s were synchronized and immunostained against LINE 1 ORF1 protein. Fluorescence intensity levels of the protein were measured and mean cytoplasmic levels were plotted. Representative confocal images for LINE 1 ORF1p and TET1/TET1s immunostainings are shown in **C**. For all boxplots, the box represents 50% of the data, starting in the first quartile (25%) and ending in the third (75%). The line inside represents the median. The whiskers represent the upper and lower quartile. Statistical significance was tested with a paired two-samples Wilcoxon test (n.s. not significant, is given for *p*-values ≥ 0.05; one star (*) for *p*-values < 0.05 and ≥ 0.005; two stars (**) is given for values < 0.005 and ≥ 0.0005; three stars (***) is given for values < 0.0005). *N*-numbers and *p*-values are shown in Supplementary Data 1. Source data are provided as a Source Data file. Scale bar = 5 μm.

Based on the increased levels of 5mC oxidation products in cancer cells that express Tet1s and on our previous findings that Tet1-CD overexpression results in a significant increase of 5mC oxidation^37,43,44^, we addressed the catalytic activity of Tet1s compared to full-length Tet1. For this purpose, we overexpressed fluorescently tagged Tet1 or Tet1s constructs or GFP alone in C2C12 cells and 24 h later immunostained them against 5hmC. While only cells that overexpressed high levels of full-length Tet1 showed a significant global increase of 5hmC compared to the GFP control, already low expression levels of Tet1s resulted in a significant increase compared to the GFP control or Tet1 (Fig. 3A). We furthermore aimed to investigate whether the overexpression of Tet1s in C2C12 cells had similar effects as those observed in MCF7 cells with higher levels of TET1 proteins. For this purpose, we performed immunofluorescence against LINE 1 ORF1p in C2C12 cells comparing Tet1s transfected versus non-transfected cells after synchronization in S phase. Representative images of these immunofluorescence signals are shown in Fig. 3C. Grouping the cells according to their GFP-Tet1s levels as we described before (Supplementary Fig. 2B), we found an increase in the LINE 1 ORFp levels after Tet1s overexpression compared with non-transfected cells. This increase was proportional to the level of Tet1s and overall higher in late S-phase synchronized cells (Fig. 3B).

### The zinc finger domain of Tet1 impedes DNA replication association and prevents aberrant 5mC oxidation

**Fig. 4:**
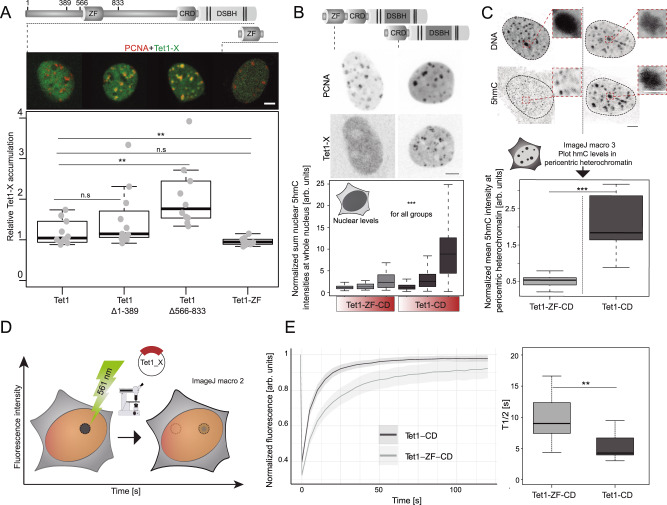
The zinc finger domain of Tet1 impedes S-phase dependent heterochromatin association and prevents aberrant 5mC oxidation. **A** Domain organization of Tet1 with locations of amino acids corresponding to N-terminal deletion mutants. Representative images of C2C12 cells in late S-phase expressing EGFP-tagged Tet1/Tet1∆1-389/Tet1∆566−833/Tet1-ZF domain and mRFP-PCNA. Boxplot shows quantification of relative accumulation. **B** C2C12 cells were transfected with mcherry-Tet1CD/Tet1-CD-ZF and EGFP-PCNA. Representative images of cells in late S-phase are shown. In situ 5hmC levels were analyzed 24 h after transfection by wide-field high-content microscopy. Boxplots show sum nuclear 5hmC levels normalized by averaged levels in non-transfected cells and sum nuclear DAPI. Cells were grouped by their mean mcherry fluorescence intensities into low, mid, and high expressing (see Supplementary Fig. 2B, C). **C** Representative images of C2C12 from 4B. Selected ROIs in pericentric heterochromatic regions were magnified. Fiji-based in situ 5hmC quantification was performed and results are shown in boxplot (5hmC levels in pericentric heterochromatin in dark-gray). **D** FRAP experiments scheme in cells expressing mcherry-tagged Tet1-CD/Tet1-CD-ZF. The mcherry signal was photobleached with a 561 nm laser and recovery of fluorescence was followed by time lapse microscopy. **E** Fluorescence recovery curves and T-half times were calculated using easyFRAP. Line plots show normalized averaged fluorescence recovery values, and error bands show the respective standard deviation. 95% confidence intervals are indicated in the plot. Representative images are shown in Supplementary Fig. 3C. For all boxplots, the box represents 50% of the data, starting in the first quartile (25%) and ending in the third (75%). The line inside represents the median. The whiskers represent the upper and lower quartile. Statistical significance was tested with a paired two-samples Wilcoxon test (n.s. not significant, is given for *p*-values ≥ 0.05; one star (*) for *p*-values < 0.05 and ≥ 0.005; two stars (**) is given for values < 0.005 and ≥ 0.0005; three stars (***) is given for values < 0.0005). N-numbers and *p*-values are shown on Supplementary Data 1. Source data are provided as a Source Data file. Scale bar = 5 μm. White scale bar = 2.5 μm.

One major functional domain that sets apart Tet1 and Tet1s is the N-terminal zinc finger (ZF) domain only present in the full-length protein (Fig. 1A). The ZF-domain has before been implicated in targeting Tet1 to non-methylated chromatin^14^. Besides the ZF-domain one additional regulatory domain in the very N-terminus was recently identified and termed BC (before zinc finger) domain^16^. We, therefore, tested the subnuclear localization of different N-terminal deletion mutants of Tet1 and also its ZF-domain alone during late S-phase. Hence, C2C12 cells were transfected with mRFP-PCNA and constructs encoding GFP-tagged Tet1∆1-389 lacking the BC-domain, Tet1∆566-833 lacking the ZF-domain or Tet1-ZF, the zinc finger domain alone. Cells in late S-phase were imaged live 8 h post-transfection and the relative Tet1 accumulation at PCNA foci of late S-phase cells was quantified as described above. While Tet1 constructs containing the zinc finger domain showed a very homogeneous nuclear pattern and no accumulation, Tet1∆566-833 showed a significantly increased accumulation (Fig. 4A). Based on this finding, we investigated the subnuclear localization of a synthetic construct, we termed Tet1-ZF-CD, composed of the Tet1 zinc finger domain fused to Tet1-CD. In contrast to Tet1-CD, the minimal catalytically active part of Tet1 recruited to replicating heterochromatin (Fig. 2E), Tet1-ZF-CD did not localize to sites of ongoing DNA replication in heterochromatin (Fig. 4B). We continued to investigate differences in the catalytic activity of Tet1-CD and Tet1-ZF-CD. For this purpose, C2C12 were transfected with either of the two constructs and 24 h later, 5hmC levels were detected by immunostaining. Tet1-CD increased 5hmC significantly more than Tet1-ZF-CD, independent of the respective overexpression levels (Fig. 4B, bottom). Analyzing cells that overexpressed either construct, we found the 5hmC staining pattern to be markedly different. While the 5hmC signal in cells transfected with Tet1-CD overlapped with DAPI-dense regions, hence, pericentric heterochromatin, Tet1-ZF-CD overexpressing cells showed only small punctated signals outside of pericentric heterochromatin (Fig. 4C). We, therefore, analyzed the 5hmC levels in heterochromatin, by masking the cells based on their DAPI signal. Again, Tet1-CD transfected cells showed a significant increase of 5hmC compared to Tet1-ZF-CD overexpressing cells (Fig. 4C, bottom). Dotplots for Fig. 4B, C are shown in Supplementary Fig. 3A, B. Based on these findings, we investigated differences in mobility and DNA binding kinetics between Tet1-CD and Tet1-ZF-CD since the zinc finger domain facilitates binding and its deletion results in decreased chromatin loading^16^. To this end, we performed fluorescence recovery after photobleaching (FRAP) experiments in C2C12 transfected with Tet1-CD or Tet1-ZF-CD (Fig. 4D). Eight hours after transfection, FRAP measurements were performed by selecting cells with a homogeneous nuclear distribution of these proteins. Representative images of FRAP experiments are shown in Supplementary Fig. 3C. Compared with freely nuclear distributed Tet1-CD, Tet1-ZF-CD had notably slower recovery kinetics, thus, a decreased mobility indicated by a significantly increased halftime (Fig. 4E).

### S-phase localization of Tet1s is independent of its catalytic activity and substrate

To further test, whether the observed localization of Tet1-CD depends on its catalytic activity, we addressed the localization of a catalytically dead mutant (Tet1-CDmut), which is still able to bind its substrate but cannot oxidize it due to point mutations in its cofactor binding sites (H1652Y, D1654A)^44^. Albeit being catalytically impaired, Tet1-CDmut localized to late-replicating heterochromatin like the catalytically active Tet1-CD (Fig. 2E). Thus, catalytic activity is dispensable for this localization.

To furthermore exclude substrate-dependent localization, we tested whether the observed localization of Tet1 depends on 5mC abundance and how the loss of 5mC can affect the observed accumulation at replicating heterochromatin. For this purpose, we made use of cells that are deficient for the maintenance methyltransferase Dnmt1, which colocalizes with PCNA during DNA replication in pericentric heterochromatin^48^. Mouse embryonic fibroblasts deficient for Dnmt1, were also deficient for p53 (MEF-PM), as primary fibroblasts from Dnmt1 negative and p53 proficient embryos proved nonviable^49^. Cells deficient for Dnmt1, are characterized by global hypomethylation^48,49^, and retain only residual levels of 5mC in major satellite repeats, which is accompanied by decondensed pericentric heterochromatin^50^. This makes them a suitable model to study the effects of 5mC depletion on Tet1s localization during ongoing DNA replication. To this end, we co-transfected MEF-PM and MEF control cells, with mcherry-Tet1s, miRFP-PCNA and EGFP-MaSat, a synthetic polydactyl zinc finger protein^51^ binding to pericentric, major satellite containing, heterochromatin and imaged the cells live approximately 8 h later, to assess the localization of Tet1s during late S-phase. The control cells, and also MEF-PM, showed a clear accumulation of Tet1s at PCNA labeled pericentric heterochromatin (Supplementary Fig. 4A), indicating that the global loss of DNA methylation does not affect the association of Tet1s with sites of ongoing DNA replication in heterochromatin.

### S-phase localization of Tet1s is dependent on Uhrf1 via its DNA binding domain but not its E3-ligase activity

**Fig. 5:**
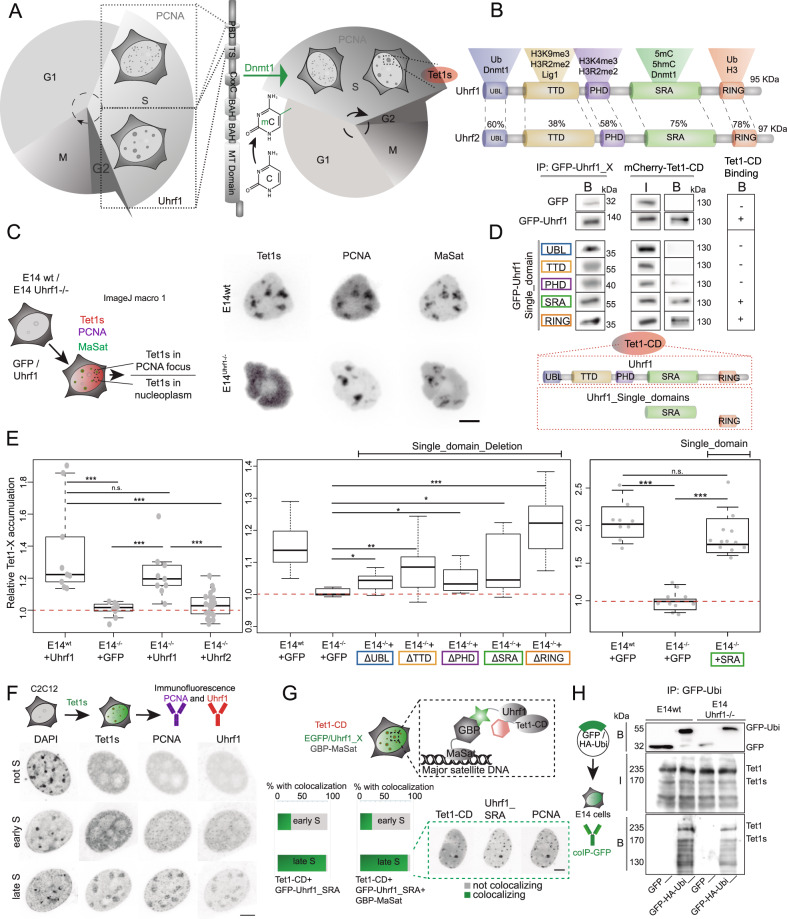
Uhrf1 physically interacts with Tet1 and is required for its S-phase localization. **A** DNA methylation maintenance throughout the cell cycle: Dnmt1 is recruited to sites of ongoing DNA replication by Uhrf1 and PCNA, ensuring faithful inheritance of the DNA methylome. Tet1s is recruited to heterochromatin during late S-phase. **B** Domain organization of Uhrf1 and Uhrf2: ubiquitin-like (UBL) domain, histone modifications binding tandem tudor domain (TTD) and plant homeodomain (PHD), DNA interacting SET and RING associated (SRA) domain and the really interesting new gene (RING) domain. Sequence homology is shown in percentage. **C** Representative images of wild-type E14 mouse embryonic stem cells or Uhrf1-deficient cells (E14 Uhrf1^−/−^) expressing mcherry-Tet1s, miRFP-PCNA and EGFP-MaSat. **D** HEK293-EBNA cells were transfected with EGFP/EGFP-Uhrf1 and mcherry-Tet1CD. Cell extracts were analyzed by immunoprecipitation using a GFP-binding nanobody and western blotting. Cut-outs show the bound GFP-fractions and the input (I) and bound (B) mcherry fractions. **E** Boxplots depict quantification of Tet1s accumulation in E14 wildtype and E14^−/−^ co-transfected with PCNA, Tet1s and either Uhrf1 (left) or different Uhrf1 mutant constructs (mid/right boxplot). Representative images shown in Supplementary Fig. 4B, C. **F** Uhrf1 and PCNA immunostaining in C2C12 cells transfected with EGFP-Tet1s. Representative images for 3 independent experiments are shown. **G** F3H assay in C2C12 transfected with miRFP-PCNA, mcherry-Tet1-CD, EGFP-Uhrf or EGFP, and GBP-MaSat. Percentage of cells with Tet1-CD localized at pericentric heterochromatin. **H** Ubiquitination of Tet1/Tet1s assayed by immunoprecipitation: E14 wildtype and E14^−/−^ were transfected with EGFP or EGFP-HA-tagged ubiquitin, immobilized using GFP-binding nanobody, and analyzed by western blotting (antibodies against GFP, Tet1/Tet1s and Dnmt1). The cut-outs show the bound GFP-fractions and the input and bound Tet1/Tet1s fractions. Two independent experiments were performed. For all boxplots, the box represents 50% of the data, starting in the first quartile (25%) and ending in the third (75%). The line inside represents the median. The whiskers represent the upper and lower quartile. Statistical significance was tested with a paired two-samples Wilcoxon test (n.s. not significant, is given for *p*-values ≥ 0.05; one star (*) for *p*-values < 0.05 and ≥ 0.005; two stars (**) is given for values < 0.005 and ≥ 0.0005; three stars (***) is given for values < 0.0005). N-numbers and p-values are shown in Supplementary Data 1. Source data are provided as a Source Data file. Scale bar = 5 μm.

Next, we addressed whether the loss of the accessory protein Uhrf1, which plays an important role in Dnmt1 recruitment for DNA methylation maintenance (Fig. 5A)^48^, could affect Tet accumulation. The multi-domain protein Uhrf1 (Fig. 5B) is mostly implicated to serve as a facilitating factor for Dnmt1 mediated DNA methylation maintenance. This is achieved by interpreting the combined information of the DNA methylation status and different histone modifications in the vicinity of hemi-methylated CpGs during ongoing DNA replication. This triggers the E3 ubiquitin ligase activity of Uhrf1 towards lysines in the histone H3 tail, which recruits Dnmt1^5,7^. The loss of Uhrf1 is accompanied by severe global hypomethylation, similar to the loss of Dnmt1. As loss of Uhrf1 is eventually lethal during embryonic development and differentiation, the effects of Uhrf1 deficiency were tested in mouse embryonic stem cells (ESC)^48,52^, specifically in E14 mouse embryonic stem cells lacking Uhrf1 (Uhrf1^−/−^). Wild-type and Uhrf1-deficient cells were transfected with mcherry-Tet1s, miRFP-PCNA and EGFP-MaSat and imaged live approximately 10 h post-transfection. While E14 wild-type cells showed a clear colocalization of Tet1s, PCNA and MaSat, no accumulation of Tet1s was observed in Uhrf1-deficient E14 cells, at PCNA marked heterochromatin, i.e. during replication (Fig. 5C). To map interactions of Uhrf1 and Tet1-CD, co-immunoprecipitation experiments were performed. To this end, GFP-tagged wild-type Uhrf1 or five different constructs with single domains of Uhrf1 were co-overexpressed together with mcherry-Tet1-CD in HEK293-EBNA cells. Immunoprecipitation was performed with a GFP-binding nanobody (GBP)^53^ and analyzed by western blotting with antibodies against GFP and RFP. In doing so, we found wild-type Uhrf1 and also the SRA or the RING domain alone to be able to pull down Tet1-CD (Fig. 5D).

To test whether Tet1s localization at heterochromatin could be rescued in Uhrf1-deficient E14 ESCs, the cells were transfected with GFP, GFP-Uhrf1 or GFP-Uhrf2 together with mcherry-Tet1s and miRFP-PCNA (Fig. 5E). Cells with a heterochromatic replication pattern were imaged approximately 14 h after transfection and the accumulation of Tet1s in PCNA foci was scored. For this, three regions inside and outside of PCNA foci were chosen, the respective mcherry-Tet1s levels were measured and the signal in heterochromatin was divided by the nucleoplasmic signal. E14 Uhrf1^−/−^ transfected with GFP as control showed nearly no elevated Tet1s levels at replicating heterochromatin. In contrast, expression of Uhrf1, but not Uhrf2, could rescue the localization of Tet1s and resulted in a significant accumulation in heterochromatin (Fig. 5E-left and Supplementary Fig. 4B). In addition, we tested if any of the single domain deletion mutants of Uhrf1 was able to rescue Tet1s localization. Interestingly, from the two domains that were able to pull down Tet1-CD (SRA and RING domain) the deletion of the RING domain (ΔRING), did not affect the rescue of the Tet1s accumulation at late-replicating heterochromatin, while deletion of the SRA domain (ΔSRA) resulted in levels of accumulation similar to E14 Uhrf1^−/−^ transfected with GFP (Fig. 5E-middle). On the other hand, E14 Uhrf1^−/−^ cells transfected only with the SRA domain showed levels of Tet1s accumulation similar to E14 wild type (Fig. 5E-right and Supplementary Fig. 4C). Hence, the association of the SRA domain of Uhrf1 with heterochromatin is sufficient to target Tet1-CD to these regions. To better characterize the Tet1s-Uhrf1 interaction, we performed immunofluorescence against PCNA and Uhrf1 in C2C12 cells transfected with EGFP-Tet1s. Interestingly, a clear association of Uhrf1 to replicating DNA was found from early to late S-phase, and cells showed a clear colocalization between PCNA, Tet1s and Uhrf1 at replicating heterochromatin. Additionally, both PCNA and Uhrf1 patterns looked similar: Uhrf1 signal matched with replication foci during S-phase, while a homogeneous nuclear distribution was found in non-S-phase cells (Fig. 5F). Next, we made use of a fluorescent three-hybrid assay (F3H) to further analyze the timing of the interaction between Tet1-CD and Uhrf1_SRA during S-phase. In this assay, the major satellite recognizing zinc finger protein described before, fused to a GFP-binding nanobody (GBP-MaSat)^50^ is co-transfected with a GFP-fusion protein as bait and a differently tagged protein as prey. GFP or GFP-fusion proteins are tethered to pericentric heterochromatin and a colocalization with the prey protein is observed in case of protein-protein interactions. If a cell-cycle independent protein-protein interaction is observed, this colocalization persists throughout the different S-phase and non-S-phase stages, as it was reported, for example, for Mbd1^43^. Hence, we co-transfected miRFP-PCNA as a cell cycle S-phase marker together with mcherry-Tet1-CD and GFP-Uhrf1_SRA with or without GBP-MaSat to anchor the GFP at pericentric heterochromatin. GFP-positive cells with S-phase patterns were imaged live 8 h post-transfection. While in cells with a GBP-MaSat mediated targeting resulted in a colocalization of mcherry-Tet1-CD with GFP-Uhrf1_SRA in 20% of scored early S-phase cells, we observed an increase to 95% during late S-phase. Cells without GBP-MaSat showed colocalization only during late S-phase (Fig. 5G). Taken together, this in vivo interaction assay suggests an S-phase substage dependent Tet1-CD/Uhrf1 interaction since a cell-cycle dependent protein-protein interaction is observed with colocalization observed only during late S-phase. Tethering Uhrf1 to pericentric heterochromatin could not initiate a premature Tet1-CD recruitment before late S-phase, which furthermore hinted to a replication dependent recruitment mechanism for Tet1s.

We next investigated the mechanism underlying the requirement of the E3 ubiquitin-protein ligase Uhrf1 for Tet1s recruitment to replicating heterochromatin. Since it was shown that overexpression of Uhrf1 enhances ubiquitination levels of Dnmt1 in vivo^54^ and two studies identified monoubiquitination of a conserved lysine residue within the CRD of all three Tet proteins by the CRL4(VprBP) E3-ligase complex to modulate their catalytic activity and chromatin association^19,20^, we tested Tet1 ubiquitination levels in the E14 wild type cells versus E14 Uhrf1^−/−^. For this purpose, we transfected E14 cells with GFP-HA-ubiquitin or GFP alone as negative control. Immunoprecipitation was performed with GFP-binding nanobody (GBP) and analyzed by western blotting with antibodies against GFP (negative control) and Tet1/Tet1s. We could pull down Tet1 and also Tet1s from wild-type E14 and also E14 Uhrf1^−/−^ cells (Fig. 5H). This shows that, while Tet1s recruitment to replicating heterochromatin is abrogated in E14 Uhrf1^−/−^ cells, ubiquitination of Tet1s still takes place. Similar results were reproduced in HEK293-EBNA cells co-transfected with GFP-HA-ubiquitin and mcherry-Uhrf1 and compared with cells only transfected with GFP-HA-ubiquitin. Overexpression of Uhrf1 was found to increase ubiquitination of Dnmt1 but did not increase ubiquitination of Tet1/Tet1s (Supplementary Fig. 4D). As we found Tet1s recruitment to sites of late DNA replication to be unaffected by DNA hypomethylation but affected by loss of Uhrf1, which showed a clear replication pattern in immunofluorescence (Fig. 5F), we deemed replication association to be more important than heterochromatin binding.

### Tet1s recruitment to heterochromatin is dependent on DNA replication and its CRD but not on its PCNA binding domain

In order to test if replication association was more important than heterochromatin binding, we made use of a system we developed before, based on the decoupling of the replisome via reversible inhibition of eukaryotic DNA polymerases by aphidicolin treatment^55,56^. The polymerase inhibition via aphidicolin leads to the disassembly of proteins involved in DNA replication elongation like PCNA^55,57^. C2C12 cells were transfected with mcherry-Tet1-CD and miRFP-PCNA. As control, Z-stacks of the cells were acquired before adding aphidicolin or DMSO, and after the addition of aphidicolin cells were imaged every 5 min over a period of 30 min (Supplementary Fig. 4E). Already after 5 min of drug treatment, a clear reduction of Tet1-CD accumulation and PCNA dissociation from replicating heterochromatin was observed, and levels of Tet1-CD relative accumulation at the different time points were measured as was explained before. This observation confirmed that Tet1-CD recruitment to heterochromatin during late S-phase occurs in a replication dependent manner.

Many proteins that associate with sites of ongoing DNA replication, do so via the interaction with the clamp loader protein PCNA. Dnmt1, for example, harbors a so-called PCNA binding domain (PBD), a short peptide with a conserved sequence motif that facilitates this interaction. The consensus sequence of classical PBDs is characterized by an initial glutamine (Q), followed by two variable amino acids, a hydrophobic amino acid, like leucine (L) or isoleucine (I), two variable residues and finally two aromatic amino acids, like phenylalanine (F), tryptophan (W), tyrosine (Y) or histidine (H). This consensus sequence is often followed by basic residues like arginine (R) or lysine (K)^58^ (Supplementary Fig. 4F). Interestingly, PCNA was reported to be an interactor of Tet1 and to regulate its dioxygenase activity throughout the cell cycle and, thereby, protect cells from aberrant DNA methylation^59^. To identify a putative PBD in Tet1, and potentially also in Tet2 and Tet3, the respective amino acids sequences were screened for the just described consensus sequence. While there were no hits in the sequences of Tet2 or Tet3, a short peptide that harbors a similar sequence was identified in the CRD of Tet1, we termed Tet1 putative PBD (Tet1-pPBD). However, a GFP-tagged Tet1-pPBD showed no accumulation at sites of ongoing DNA replication (Supplementary Fig. 4F). As Tet1-CD is the minimal catalytically active part of Tet1 that localizes to replicating heterochromatin, we sought to investigate the localization of the Tet1-CD main domains, the CRD and the DSBH. For this, we overexpressed GFP-tagged Tet1-CRD or Tet1-DSBH together with RFP-PCNA in C2C12 cells and quantified their accumulation in heterochromatic PCNA foci as described above. Here, we found a slight enrichment of the CRD but not the DSBH (Supplementary Fig. 5A), while neither domain alone was able to increase 5hmC levels (Supplementary Fig. 5B). This led us to delete the CRD from Tet1s and investigate the S-phase localization of the resulting deletion mutant. The respective fusion protein showed a clear nuclear signal but no S-phase accumulation (Supplementary Fig. 5C). Additionally, no increased 5hmC formation was observed, when overexpressing Tet1s-ΔCRD (Supplementary Fig. 5D). These findings match with structural data of Tet2, showing that the CRD and DSBH together form a compact catalytic core^60^ to facilitate the dioxygenase activity of Tet proteins.

### A conserved lysine in Tet1 is crucial for its S-phase heterochromatin localization and targeted catalytic activity

**Fig. 6:**
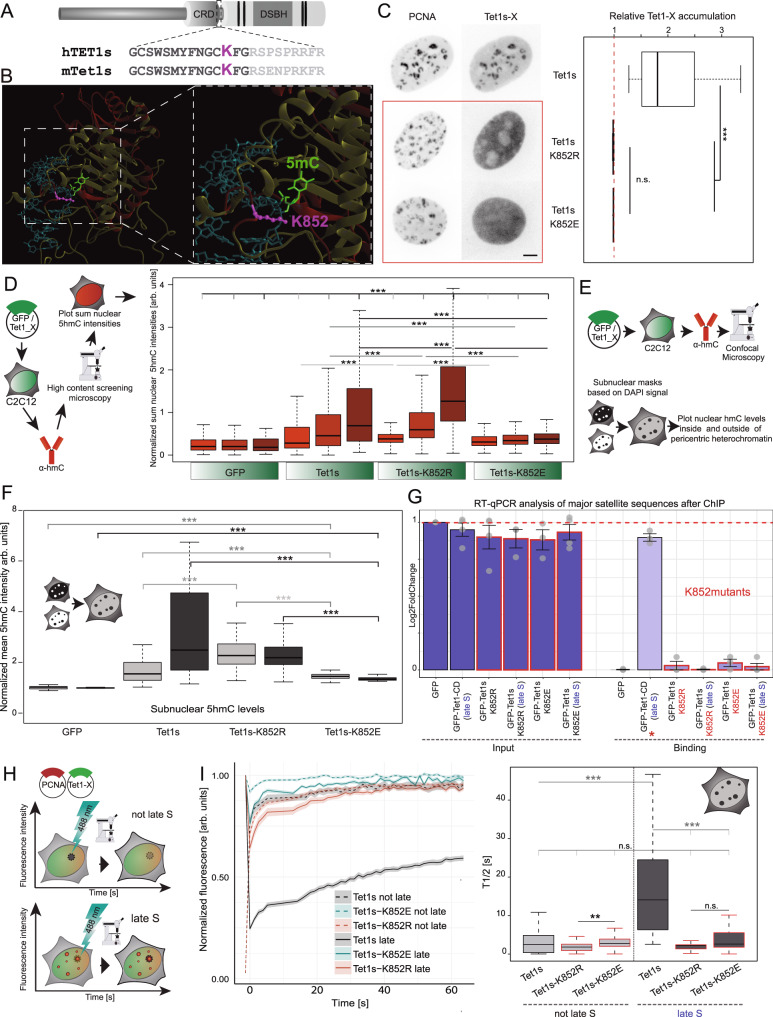
Lysine residue K852 in the CRD domain of Tet1 is crucial for Tet1s S-phase localization and targeted catalytic activity. **A** Localization of the conserved lysine residue K852 in Tet1s and sequence alignment between human and mouse Tet1. **B** Model of mouse Tet1s generated by homology modeling on the refined human Tet2 crystal structure (4NM6). The CRD is shown in red (conserved lysine 852 in pink), and the DSBH in yellow. The bound DNA helix appears in blue with 5mC flipped out of the double helix in green. **C** Representative images of C2C12 cells expressing mRFP-PCNA and EGFP-Tet1s/Tet1s-K852R/Tet1s-K852E. Boxplots showing heterochromatin accumulation of Tet1-X. **D** C2C12 cells from C were immunostained against 5hmC 24 h after transfection. Nuclear 5hmC levels were measured by high-content microscopy. Boxplot shows sum nuclear 5hmC levels normalized to the averaged 5hmC levels of background cells and against sum nuclear DAPI intensity. Cells were grouped according to their mean EGFP fluorescence intensities (see Supplementary Fig. 2B, C). **E**, **F** Fiji-based in situ 5hmC analysis: Binary nucleoplasm and heterochromatin masks were generated and mean fluorescence intensities in the respective areas were measured. Boxplots in **F** show 5hmC levels in pericentric heterochromatin (dark-gray) and the surrounding nucleoplasm (light-gray). Representative images and constitutive heterochromatin relative areas are shown in Supplementary Fig. 5E, F. **G** Chromatin immunoprecipitation experiments followed by qPCR for MajSat sequences. C2C12 cells were transfected with Tet1s-K852R/Tet1s-K852E and Tet1-CD (positive control) and synchronized in G1/late S phase. Barplots show the average value of amplification levels in input and chromatin binding fractions normalized with GFP-input (red line). The error bars represent the standard deviation with a 95% confidence interval. **H** FRAP experiments in transfected C2C12 cells (EGFP-tagged Tet1s/Tet1s-K852R/Tet1s-K852E). The EGFP signal was photobleached with a 488 nm laser. **I** For analysis, fluorescence recovery curves and T-half times were calculated using easyFRAP. Line plots show normalized averaged fluorescence recovery values, and error bands show the respective standard deviation. 95% confidence intervals are indicated in the plot. For all boxplots, the box represents 50% of the data, starting in the first quartile (25%) and ending in the third (75%). The line inside represents the median. The whiskers represent the upper and lower quartile. Statistical significance was tested with a paired two-samples Wilcoxon test (n.s. not significant, is given for *p*-values ≥ 0.05; one star (*) for *p*-values < 0.05 and ≥ 0.005; two stars (**) is given for values < 0.005 and ≥ 0.0005; three stars (***) is given for values < 0.0005). *N*-numbers and *p*-values are shown in Supplementary Data 1. Source data are provided as a Source Data file. Scale bar = 5 µm.

In contrast to the putative but non-functional PBD that is only found in Tet1, all three Tet proteins share a conserved lysine residue in their CRD, which was shown to become monoubiquitinated in a CRL4(VprBP) dependent manner. The lysine itself resides within a short peptide that is conserved between all three Tet protein family members and also between human and mouse Tet (Fig. 6A). This short amino acid stretch was shown to stabilize the DNA around the modified cytosine target, by interacting with the phosphate backbone^60^ (Fig. 6B). Mutations of the respective lysine have been found to result in a decrease or even loss of catalytic activity in all three Tet proteins and monoubiquitination of this lysine was found to be important during oocyte development and for Tet2 chromatin binding, but its biological consequences have mostly been addressed only for Tet2 and Tet3^19,20^. We therefore mutated lysine (K) 852 in Tet1s to glutamate (E) or arginine (R) to invert or keep the respective charge and abrogate a putative ubiquitination. We continued to investigate the DNA replication association capability of the respective constructs and their effect on 5hmC generation. Mutating Tet1s lysine 852 to a glutamate (Tet1s-K852E) or arginine (Tet1s-K852R) destroyed the ability to localize to sites of ongoing DNA replication in heterochromatic regions (Fig. 6C). Interestingly and in contrast to our observations on the localization, global 5hmC levels were even higher in cells that overexpressed Tet1s-K852R compared to wild-type Tet1s. Cells transfected with the glutamate mutant Tet1s-K852E, on the other hand, showed a very minor 5hmC increase (Fig. 6D). This observation is in line with data from structural studies on Tet2, where a glutamate at this position disrupted the stabilizing effect of the peptide stretch on the phosphate backbone of the DNA^60^. To analyze subnuclear 5hmC deposition in more detail, we acquired high magnification Z-stacks and masks were created to threshold the heterochromatin and the surrounding nucleoplasm to subsequently measure respective mean fluorescence intensities (Fig. 6E). While Tet1s transfected cells showed a significant increase of 5hmC mostly in heterochromatin (Fig. 6F and Supplementary Fig. 5E), 5hmC levels were also significantly elevated in the nucleoplasm of Tet1s-K852R transfected cells, explaining the stronger global increase (Fig. 6F). Tet1s-K852E transfection, on the other hand, resulted only in minor 5hmC depositions in heterochromatin or nucleoplasm, compared to Tet1s or Tet1s-K852R. In addition, we found that cells that overexpressed Tet1s showed less compacted pericentric heterochromatin compared with non-transfected cells or cells transfected with Tet1s-K852E or Tet1s-K852R (Supplementary Fig. 5E). To quantify this observation, we divided the DAPI-dense pericentric heterochromatin area by the total nuclear area and found that Tet1s transfected cells exhibit higher relative heterochromatin area compared with Tet1s-K852E and Tet1s-K852R transfected cells. This is accompanied by a decrease in the standard deviation for DAPI intensity values in Tet1s transfected cells due to the decondensation of highly compact heterochromatic regions (Supplementary Fig. 5F). This prompted us to perform a detailed quantitative analysis of changes in chromatin structure. We analyzed the 3D nuclear landscape and the spatial nuclear DNA organization using confocal microscopy, which allowed us to assess different chromatin compaction levels (1 to 7) in individual cell nuclei^61^. Comparing Tet1 versus Tet1s transfected cells in late S-phase, we found differences in the distribution of the sub-compartments that were calculated based on the DAPI intensities. For Tet1s, an increase in the fraction for lower compaction classes was observed, together with a reduction in higher compacted classes, in comparison to Tet1 transfected cells. Overall, this indicates a reduction in the fraction of inactive compartments (compacted core of chromatin cluster), which is in line with previous results showing decondensation of heterochromatic regions (Supplementary Fig. 5F). In summary, mutating the conserved lysine to arginine leads to increased levels of 5hmC globally in contrast to Tet1s. Overexpression of the latter results in increased levels of 5hmC in heterochromatin and accompanied decondensation of these regions, producing significant changes in heterochromatin structure.

To verify the lack of accumulation at replicating heterochromatin for Tet1s-K852E and Tet1s-K852R mutants, we performed chromatin immunoprecipitation followed by qPCR as we described before (Fig. 2G). Accordingly, qPCR amplification of MajSat sequences was found for Tet1-CD during late S, while no amplification occurred for Tet1s-K852E and Tet1s-K852R independently of the cell cycle stage (Fig. 6G). The differences in the recruitment to replicating heterochromatin and ChIP results, prompted us to investigate whether Tet1s-K852E and Tet1s-K852R DNA binding kinetics are affected. To this end, we performed FRAP analysis to measure the mobility of Tet1s and Tet1s lysine mutants. Due to the unique ability of Tet1s to localize at replicating heterochromatin during late S-phase, and to the higher mobility of Tet1-CD found in previous FRAP experiments (Fig. 4E), we decided to perform these analyses making a distinction between not late S (homogeneous nuclear distribution of Tet1s) and late S-phase (when Tet1s is localized at replicating pericentric heterochromatin). To this end, we co-transfected C2C12 cells with GFP-Tet1s, GFP-Tet1s-K852E or Tet1s-K852R together with mRFP-PCNA. Eight hours after transfection, FRAP measurements were performed by bleaching a region of the nucleus during not late S, excluding those areas with PCNA replication foci, and bleaching a heterochromatic region during late S-phase where PCNA and also Tet1s was located (Fig. 6H). Compared to homogeneously distributed Tet1s and its lysine mutants during not late S, heterochromatin accumulated Tet1s in late S-phase showed much slower recovery kinetics and, thus, decreased mobility. In contrast to this, Tet1s-K852E and Tet1s-K852R had similar kinetics independently of their S-phase substage with fast recovery kinetics and a high mobility (Fig. 6I).

### The CRL4(VprBP) complex ubiquitinates Tet1s and is needed for Tet1s S-phase association via Uhrf1 interaction

We next checked the levels of ubiquitination of Tet1s-K852E and Tet1s-K852R compared with Tet1s, and prospective changes in these levels by the overexpression of either VprBP or Uhrf1. VprBP (Vpr-Binding Protein/DDB1 And CUL4 Associated Factor 1) is a serine/threonine kinase^62^ that serves as an adapter protein for DDB1 (DNA damage-binding protein 1) and Cul4A/B (Cullin-4A/B)^63^, binds Tet proteins and, thereby, brings them together with DDB1 and CRL4 E3-ligase complex^19,20^. For that purpose, we co-transfected HEK293-EBNA cells with HA-ubiquitin and GFP-tagged Tet1s, Tet1s-K852E or Tet1s-K852R, and mcherry-Uhrf1 or mcherry-VprBP. Immunoprecipitation was performed with GFP-binding nanobody (GBP) and analyzed by western blotting with antibodies against GFP and HA. As expected, we found Tet1s to be able to pull down HA-ubiquitin, but this ubiquitin pulldown was reduced for Tet1s-K852E and Tet1s-K852R, pointing to a reduction in ubiquitination of the lysine mutants (Supplementary Fig. 6A). By these pulldown experiments, we validated that ubiquitination of Tet1s took place and was negatively affected in Tet1s-K852E and Tet1s-K852R mutants as was the recruitment to replicating heterochromatin. Overexpression of Uhrf1 did not increase ubiquitination for Tet1s (see also Supplementary Fig. 4D) nor of Tet1s-K852E or Tet1s-K852R. Interestingly, overexpression of VprPB increased ubiquitination of Tet1s but not Tet1s-K852E or Tet1s-K852R (Supplementary Fig. 6A). As the pull-downs and ubiquitination assays were performed by ectopically expressing tagged ubiquitin, we tested the ubiquitination of Tet1-CD with endogenous levels of ubiquitin. For this, HEK293-EBNA cells were transfected with GFP-Tet1-CD and GFP-PCNA as a positive control, and treated with MG-132^64^, a potent cell-permeable proteasome inhibitor, followed by co-immunoprecipitation and western blot. Ubiquitinated Tet1-CD and PCNA were detected using an anti-ubiquitin antibody (Supplementary Fig. 6B) validating the results with tagged ubiquitin.

**Fig. 7:**
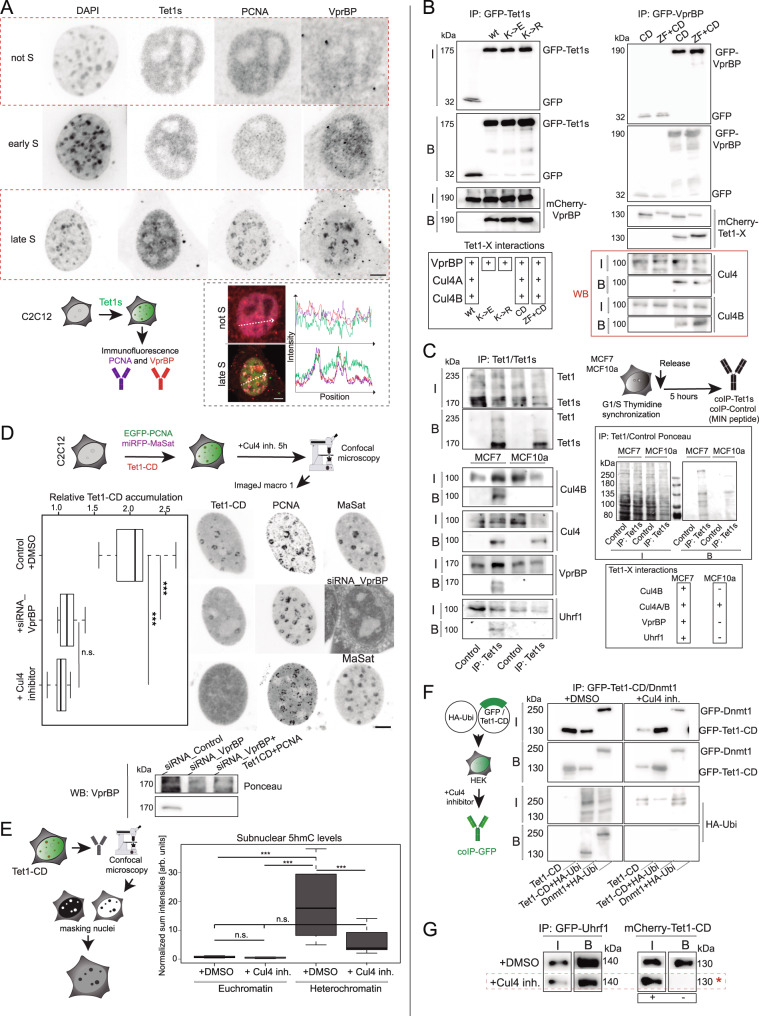
The CRL4(VprBP) complex ubiquitinates Tet1s and this is needed for Tet1s recruitment to late-replicating heterochromatin. **A** VprBP and PCNA immunostaining in C2C12 cells expressing EGFP-Tet1s: representative images of 3 independent experiments and line-profile analysis are shown. **B** HEK293-EBNA cells were transfected with EGFP or EGFP-tagged Tet1-X/VprBP, or mcherry fusions (VprBP/Tet1-X). Cell extracts were analyzed by immunoprecipitation with immobilized GFP-binding nanobody, followed by detection with antibodies against GFP, RFP, Cul4 and Cul4B. The cut-outs show input/bound GFP and input/bound mcherry fractions. **C** Endogenous co-immunoprecipitation: MCF cell extracts were analyzed using immobilized Tet1/Tet1s followed by detection with antibodies against Tet1/Tet1s, Cul4B, Cul4, VprBP and Uhrf1. MIN antigen (attP-peptide) was used as negative control. The cut-outs show the input/bound Tet1/Tet1s fractions. **D** C2C12 cells were transfected with mcherry-Tet1-CD, EGFP-PCNA and miRFP-MaSat. Additionally, cells were transfected with siRNA_VprBP or treated with pevonedistat to indirectly inhibit Cul4/DMSO for 5 h before live-cell imaging. Representative images are shown. Boxplots depict Tet1-CD accumulation at heterochromatin. Western blotting with antibody against VprBP validates the knockdown in C2C12 cells. **E** In situ 5hmC analysis after 24 h treatment with Cul4 inhibitor. Boxplots show the quantification of 5hmC levels in C2C12 cells in euchromatin (light-gray) versus heterochromatin (dark-gray). **F** HEK293-EBNA cells were transfected with EGFP-tagged Tet1-CD/Dnmt1 and HA-ubiquitin, treated with pevonedistat/DMSO for 24 h, and analyzed by immunoprecipitation with immobilized GFP-binding nanobody and detection with antibodies against GFP and HA. The cut-outs show the input/bound GFP-fractions and the input/bound HA-Ubi fractions. **G** HEK293-EBNA cells were transfected with EGFP-Uhrf1 and mcherry-Tet1-CD and treated with pevonedistat/DMSO for 24 h. Cell extracts were analyzed by immunoprecipitation with immobilized GFP-binding nanobody, and detection with antibodies against GFP and RFP. The cut-outs show the input/bound GFP-fractions, and the input/bound mcherry fractions. In **B**, **C**, **G**, two independent experiments were performed. For boxplots, the box represents 50% of the data, starting in the first quartile (25%) and ending in the third (75%). The line inside represents the median. The whiskers represent the upper and lower quartile. Statistical significance was tested with a paired two-samples Wilcoxon test (n.s. not significant, is given for *p*-values ≥ 0.05; one star (*) for *p*-values < 0.05 and ≥ 0.005; two stars (**) is given for values < 0.005 and ≥ 0.0005; three stars (***) is given for values < 0.0005). N-numbers and *p*-values are shown in Supplementary Data 1. Source data are provided as a Source Data file. Scale bar = 5 µm.

Next, we analyzed the cell cycle dependent subnuclear localization of VprBP as the protein involved in the monoubiquitination of this lysine residue in Tet1s. For this, we performed immunofluorescence against PCNA and VprBP in fixed C2C12 cells transfected with EGFP-Tet1s with the PCNA patterns used to discriminate S-phase substages. A clear colocalization with PCNA was found, especially during late S-phase. In addition, we found colocalization of Tet1s, VprBP and PCNA at replicating heterochromatin during late S, while homogeneous cellular distribution of VprBP was found outside S-phase (Fig. 7A). In addition, we co-transfected GFP-Tet1s, mcherry-VprBP and miRFP-PCNA in C2C12 myoblasts and analyzed their cell cycle localization by live-cell time lapse microscopy. Cells in early S-phase were chosen and followed over time while acquiring Z-stack images every 20 min. Although VprBP showed a pancellular distribution, a slight cytoplasmic-to-nuclear translocation was observed from early to late S-phase (Supplementary Fig. 6C). These observations are in line with a previous study that found the chromatin association of VprBP to increase throughout S-phase^63^. Furthermore, line-profile analysis of a late S-phase replication focus showed that the VprBP signal follows the distribution of PCNA and Tet1s (Fig. 7A and Supplementary Fig. 6C). These observations together with the previous immunoprecipitation results (Supplementary Fig. 6A) suggests a model in which CRL4(VprBP) mediated Tet1s ubiquitination is responsible for its replication association in late S-phase.

To test whether Tet1s and its lysine mutants interact with CRL4(VprBP), we performed co-immunoprecipitation of tagged VprBP and Tet1-CD or Tet1-ZF-CD, where the latter clarifies a potential disruption of the interaction due to the zinc finger domain. First, we co-transfected HEK293-EBNA cells with GFP-Tet1s or its lysine mutants and mcherry-VprBP. Immunoprecipitation was performed with GFP-binding nanobody (GBP) and analyzed by western blotting with antibodies against GFP and RFP. Tet1s, Tet1s-K852E and Tet1s-K852R were able to pull down VprBP (Fig. 7B left). Secondly, we co-transfected GFP-VprBP and mcherry-Tet1-CD or mcherry-Tet1-ZF-CD and we performed immunoprecipitation as described above. In all cases, GFP-VprBP (Fig. 7B right) was able to immunoprecipitate Tet1-CD and Tet1-ZF-CD. GFP-VprBP was also able to immunoprecipitate endogenous Cul4, as shown by western blotting with antibodies against Cul4 and Cul4B (Fig. 7B bottom). To further verify Tet1-X interactions with VprBP/Cul4 and Uhrf1 at endogenous levels, we performed immunoprecipitation in MCF7 and MCF10a cells. For this purpose, we immobilized Tet1 proteins using protein G agarose beads preincubated with Tet1 antibody and analyzed cell extracts by western blotting with antibodies against Tet1/Tet1s, VprBP, Cul4, Cul4B and Uhrf1 (Fig. 7C). Endogenous Tet1 proteins were able to precipitate Cul4A/B, VprBP and Uhrf1 in the tumor cell line MCF7, which showed higher levels of Tet1s. These data indicated that Tet1s and Tet1-CD interact with VprBP independently of the mutation on the lysine or the insertion of the zinc finger domain. Immunoprecipitated Tet1 proteins from MCF10a extracts were able to pulldown Cul4 but not VprBP or Uhrf1 (Fig. 7C). The endogenous immunoprecipitation confirmed the previously mentioned interactions in HEK293-EBNA cells and also showed differences in the pull down of VprBP and Uhrf1 between these cell lines.

To further elucidate the role of Cul4A/B in Tet1s recruitment, we made use of the NEDD8-activating enzyme (NAE)-inhibitor pevonedistat to abrogate SUMOylation of Cul4^65^, which in turn prevents ubiquitination of the conserved lysine residue in Tet1s by the CRL4(VprBP) complex. In parallel, we tested the effect of the knockdown of VprBP. We imaged cells live as described before 8 h post-transfection. For C2C12 cells co-transfected with mcherry-Tet1-CD, EGFP-PCNA as a marker for S-phase progression and miRFP-MaSat as a marker for pericentric heterochromatin, we could validate Tet1-CD association with sites of ongoing DNA replication during late S-phase. The accumulation of Tet1-CD was, though, lost in cells that were transfected with siRNA_VprBP or after 5 h of treatment with pevonedistat (Fig. 7D). Moreover, the accumulation of Tet1-CD at PCNA and MaSat marked heterochromatin was quantified as described above and the knockdown of VprBP was verified by western blotting (Fig. 7D). In summary, indirect inhibition of the CRL4(VprBP) complex abrogates the recruitment of Tet1-CD to replicating heterochromatin. Importantly, immunofluorescence staining against 5hmC in Tet1-CD transfected cells after 24 h of treatment with pevonedistat resulted in significantly lower 5hmC levels in heterochromatin but not in euchromatin relative to control treated cells (Fig. 7E). This indicates that the increase on 5hmC levels after Tet1s overexpression was a consequence of Tet1s cell cycle dependent recruitment to heterochromatic regions.

Finally, we tested whether treatment with pevonedistat affects the ubiquitination of Tet1-CD and whether it negatively affects the interaction with Uhrf1, which we showed earlier (Fig. 5) to be an essential player in mediating the Tet1s subnuclear localization. For this purpose, we made use of the ubiquitination assay using extracts from HEK293-EBNA cells co-transfected with GFP-Tet1-CD or GFP-Dnmt1, as control for ubiquitination, and HA-ubiquitin. After 24 h treatment with pevonedistat or DMSO as control, we immunoprecipitated GFP-tagged proteins using the GFP-binding nanobody (GBP) and analyzed the cell extracts and precipitated material by western blotting with antibodies against GFP and the HA-tag. HA-ubiquitin was detected in the pulldown fraction of DMSO-treated cells, but not of cells treated with pevonedistat (Fig. 7F). Last, we co-transfected HEK293-EBNA cells with GFP-Uhrf1 and mcherry-Tet1CD and again treated these cells with pevonedistat or DMSO as control. After treatment with the NAE-inhibitor, GFP-Uhrf1 was not able to pulldown Tet1-CD anymore, compared with control cells (Fig. 7G). This indicates that the protein-protein interaction between Uhrf1 and Tet1-CD depends on Cul4-mediated ubiquitination of Tet1-CD.

### Tet1 and Tet1s differently affect 5mC and 5hmC levels of heterochromatic LINE 1 elements compared with euchromatic Alu elements

**Fig. 8:**
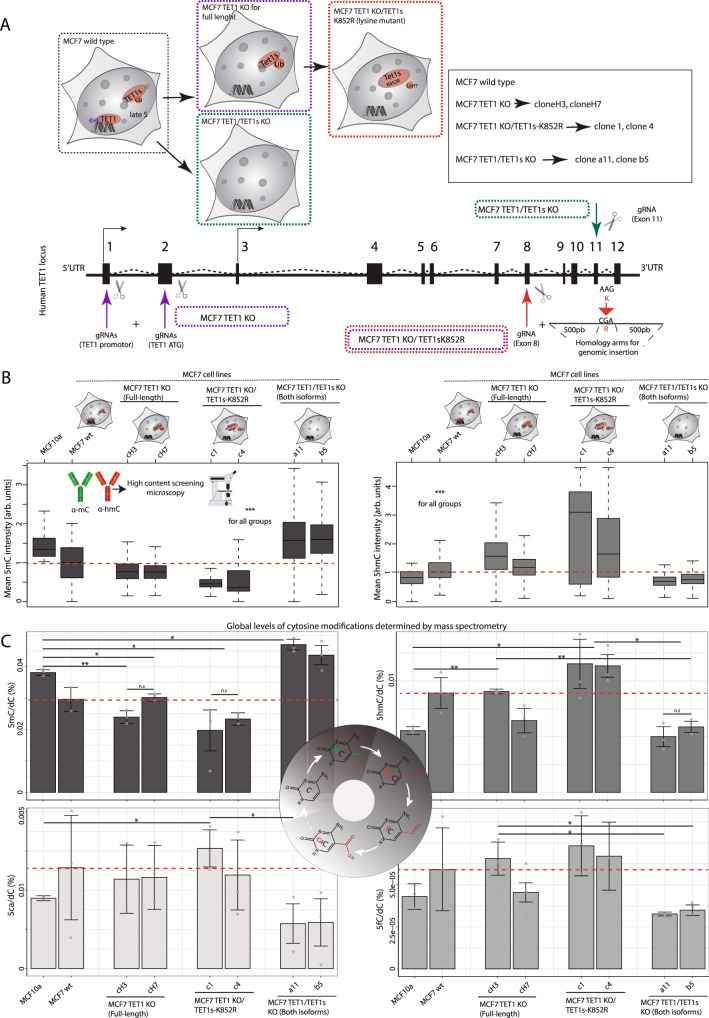
Comparison of global levels of cytosine modifications in MCF10a cell line versus MCF7 and TET1-X mutants. **A** Scheme illustrating the different MCF7 cell lines generated by CRISPR/Cas9 genome editing, showing positions of gRNA targets in the TET1 locus. MCF7 TET1 KO (full isoform KO), MCF7 TET1 KO/TET1s-K852R (full isoform KO and TET1s lysine mutant) and MCF7 TET1/TET1s KO (both isoforms KO) were generated. Two different clones were selected and used as biological replicates. **B** Immunofluorescence analysis of 5mC and 5hmC nuclear levels in MCF10a, MCF7 wild-type and MCF7 TET1-X mutants by high-content microscopy. Levels were compared with 5mC/5hmC shown in **C**. Mean intensity values were normalized to the average for MCF7 wild-type (discontinuous red line). **C** Barplots showing levels of 5mC, 5hmC, 5fC, and 5caC in genomic DNA measured by ultra-high performance liquid chromatography coupled to tandem mass spectrometry (UHPLC-MS/MS). The abundance of genomic cytosine modifications was plotted as the fraction of total modified cytosines, and DNA modification levels are expressed as percentage (%). Average levels in MCF7 wild-type cells are indicated with a discontinuous red line for all cytosine modifications. The error bars represent the standard deviation with a 95% confidence interval. For all boxplots, the box represents 50% of the data, starting in the first quartile (25%) and ending in the third (75%). The line inside represents the median. The whiskers represent the upper and lower quartile. Statistical significance was tested with a paired two-samples Wilcoxon test and One-Way ANOVA for mass spectrometry data (n.s. not significant, is given for *p*-values ≥ 0.05; one star (*) for *p*-values < 0.05 and ≥ 0.005; two stars (**) is given for values < 0.005 and ≥ 0.0005; three stars (***) is given for values < 0.0005). N-numbers and *p*-values are shown in Supplementary Data 1. Source data are provided as a Source Data file.

To relate the effect of the different Tet1 isoforms and mutants on 5mC oxidation with its physiological consequences on transcriptional noise, we generated different MCF7 cell lines using CRISPR/Cas9 genome editing (Fig. 8A). Firstly, we performed the knockout of TET1, but not of TET1s, to show the effects on cytosine modification levels of the short isoform without the interference of TET1 (Supplementary Fig. 7A–C). For the characterization of these cell lines, we performed PCR amplification and sequencing of the exon 1 to confirm the genomic deletion (Supplementary Fig. 7A). Furthermore, we performed measurements of TET1 protein levels by western blot and immunofluorescence followed by high-content microscopy analysis (Supplementary Fig. 7B, C). For immunofluorescence we used different fixation protocols to distinguish between TET1 and TET1s (the latter not fixable with formaldehyde fixation, Supplementary Fig. 2A and Supplementary Fig. 7C right). While with standard formaldehyde treatment only TET1 is fixable, the gradual increase of formaldehyde concentration increases the fixability of TET1s (Supplementary Fig. 7C left)^42^. This allowed us to detect the nuclear localization of both TET1 proteins in the MCF7 TET1 KO (Supplementary Fig. 7C center). Secondly, using the TET1 KO cell line, we generated the TET1s-K852R mutant by point mutation (Supplementary Fig. 6B and Supplementary Fig. 7D). We focused on the K to R mutant since the K to E lacks its catalytic activity and, therefore, physiological significance. We confirmed the insertion of the mutation by amplification of exon 8 by PCR followed by DNA sequencing (Supplementary Fig. 7D). Lastly, we created a full knockout for both TET1 and TET1s, using the strategy and the gRNA described in a previous study^17^. As before, western blot and immunofluorescence analysis for TET1/TET1s levels in these cell lines were performed to confirm the double knockout, in addition to PCR amplification and DNA sequencing of exon 11 (Supplementary Fig. 7E–G).

Using these cell lines, we compared cytosine modification levels of MCF7 wild-type and MCF10a cells. To this end, we used ultra-high performance liquid chromatography coupled to tandem mass spectrometry (UHPLC-MS/MS) to quantitatively assess the levels of 5mC, 5hmC, 5fC, and 5caC in genomic DNA. Two different cell clones were analyzed as biological replicates. We compared these measurements with immunofluorescence results in these newly generated cell lines for 5mC and 5hmC (Fig. 8B, C) and found that they followed a similar trend. Abundance of genomic 5mC, 5hmC, 5fC, and 5caC is plotted as the fraction of total modified cytosines, and DNA modification levels are expressed as percentage (%) (Supplementary Data 1, 2). Cytosine modification levels for MCF10a and MCF7 cells matched previous immunofluorescence results in Fig. 1C. As predicted, MCF7 TET1 KO and MCF7 TET1 KO/TET1s-K852R showed lower or similar 5mC levels compared with MCF7 wild-type, while 5hmC, 5fC and 5caC were all higher. Global DNA methylation (5mC) was increased for MCF7 TET1/TET1s KO cells to levels similar to MCF10a (with low levels of both TET1 proteins, Supplementary Fig. 1A). Along with 5mC increase, the levels of 5hmC, 5fC, and 5caC decreased from MCF7 wild-type to MCF7 TET1/TET1s KO (Fig. 8C).

**Fig. 9:**
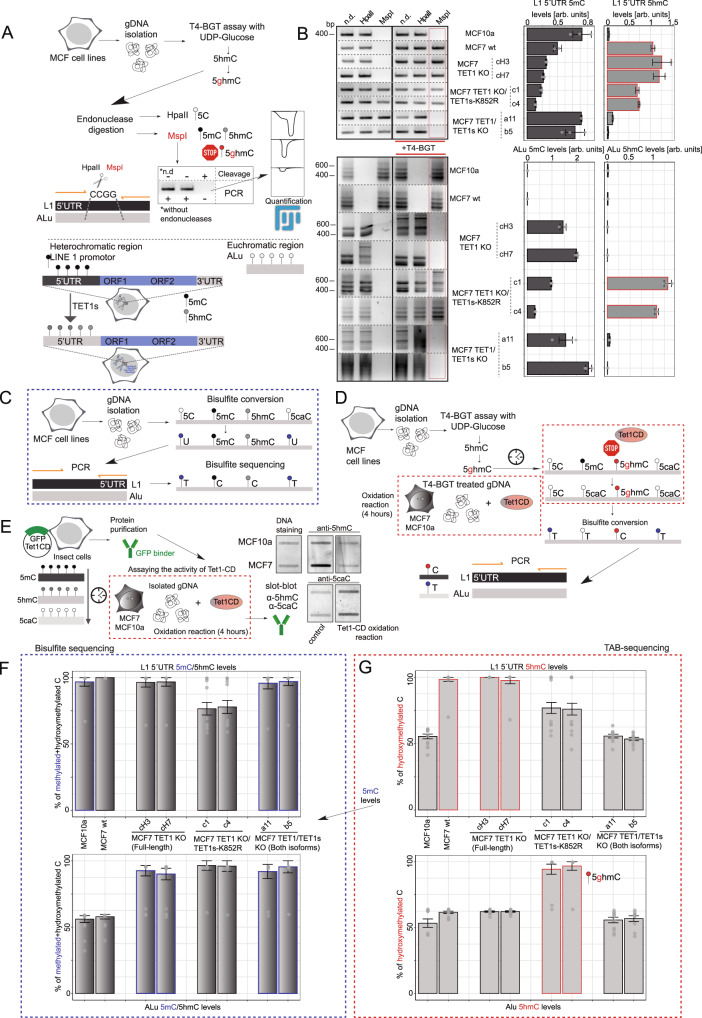
5mC and 5hmC levels at heterochromatin (LINE 1 promoter) and euchromatic loci (Alu). **A** Scheme of GluMS-PCR experiments: DNA glucosylation, MspI and HpaII digestion and PCR based 5hmC/5mC detection. **B** Cut-off of agarose gels showing PCR products (LINE 1 protomer and Alu element) after T4-BGT treatment and endonuclease digestion. Barplots showing densitometry measurements (for PCR bands) quantifying 5mC/5hmC levels. Higher levels of 5hmC are indicated with red edges in the barplot. **C** Bisulfite conversion of genomic DNA followed by PCR for amplification of LINE 1 promoter and Alu element. Unmodified or 5caC is converted to uracil and consequently be read as a T after PCR. 5mC or 5hmC are not converted, and cannot be distinguished by this method. **D** TAB (Tet-assisted bisulfite) sequencing experiments scheme. 5hmC is protected from further oxidation by incubation with T4-BGT and UDP-glucose. 5mC (but not protected 5ghmC) is oxidized to caC by Tet1-CD incubation followed by bisulfite conversion of C and 5caC and PCR. Only 5ghmC will be read as a C after PCR and sequencing. **E** Process of GFP-Tet1-CD protein purification and subsequent oxidation reaction test using gDNA. Slot blotting of DNA before and after oxidation reaction shows levels of 5hmC and 5caC. **F** Barplots showing the percentage of 5mC/5hmC at base resolution level after bisulfite conversion of unmodified cytosines, PCR and sequencing. Bisulfite sequencing experiments were performed for euchromatic versus heterochromatic loci as GluMS-PCR experiments. **G** Barplots showing the percentage of 5hmC at base resolution level after TAB-sequencing analysis. Experiment was performed with gDNA treated as described in **A**, followed by Tet1-CD oxidation reaction, bisulfite conversion and PCR. Barplots showing higher percentage of 5hmC are indicated by red outlines, and those with higher percentage of 5mC in **F** are indicated by blue outlines. For **B**, **F**, **G**, the error bars represent the standard deviation with a 95% confidence interval. Statistical significance was tested with a paired two-samples Wilcoxon test using R-studio. N-numbers and *p*-values are shown in Supplementary Data 1. Source data are provided as a Source Data file.

Next, we analyzed the occurrence of 5mC and 5hmC at selected genomic loci. We compared the 5´ UTR region of the LINE 1 DNA repeat element, as heterochromatic loci as mentioned before (see, e.g., Fig. 1F)^30,34^, versus the Alu DNA element (SINEs) as a repetitive DNA sequence located in euchromatin^32,33^. These interspersed repeat elements exist in half a million to over one million copies throughout the human genome but correlate with different chromatin states. Hence, they are well suited to report on the DNA modification state of euchromatic versus heterochromatic loci throughout the human genome. For this purpose, we performed DNA glucosylation followed by MspI and HpaII restriction enzyme digestion and PCR-based 5hmC and 5mC detection (GluMS-PCR). Genomic DNA was treated with T4-BGT, which adds glucose to 5hmC yielding 5ghmC but not to 5mC. Then, we performed endonuclease treatment with MspI and HpaII, both recognizing “CCGG” but sensitive to different methylation states. HpaII cleaves only unmodified sites, while MspI cleaves 5mC and 5hmC, but not 5ghmC. Finally, we used primers for PCR amplification of these genomic loci. The primers flank the target site of the endonucleases: if the CpG site contains 5hmC a band is detected after glucosylation (and conversion to 5ghmC) and digestion, but not in the non-glucosylated control reaction (scheme in Fig. 9A)^37,43,66^. Results of end-point PCR comparing MCF10a, MCF7 wild-type, MCF7 TET1 KO, MCF7 TET1 KO/TET1s-K852R, and TET1/TET1s KO cells (including biological replicates) are shown in Fig. 9B. Quantification of 5mC and 5hmC levels in heterochromatic and euchromatic genomic regions was performed by image analysis of density (aka intensity) of bands in agarose gels using Fiji^67^ and normalizing to non-digested samples. For LINE 1 element at heterochromatic regions, MCF10a cells showed the highest level of 5mC and levels of 5hmC close to 0 (corresponding to the absence of a band for MspI digestion), and the same was observed for MCF7 TET1/TET1s KO (Fig. 9B). In contrast, MCF7 wild-type cells showed lower levels of 5mC and higher levels of 5hmC, and these differences are increased for MCF7 TET1 KO cells, in line with mass spectrometry and immunofluorescence results (Fig. 8B, C). As expected, MCF7 TET1 KO/TET1s-K852R showed a reduction in 5hmC levels (Fig. 9B) due to the lack of TET1s accumulation on heterochromatic regions (Fig. 6C, G). Nevertheless, they still showed some levels of 5hmC in heterochromatic repeats, which is consistent with their high catalytic activity producing a global increase of 5hmC, 5fC and 5caC (Figs. 6D, F, 8C). We, next, analyzed 5mC and 5hmC levels in an Alu element as an euchromatic region^33,34^. After HpaII treatment for MCF10a and MCF7 wild-type cells, there was no PCR amplification indicating that these DNA sequences are mostly unmodified. For TET1 KO, we found an increase in 5mC that is consistent with the role of TET1 avoiding 5mC spreading into euchromatic regions. Interestingly, 5mC increase after TET1 KO is reduced and likely oxidized to 5hmC in the TET1 KO/TET1s-K852R mutants, which showed a higher catalytic activity and mobility (Fig. 6), as we demonstrated earlier. In line with these results, TET1/TET1s KO cells showed 5mC increase as observed for TET1 KO (Fig. 9B). As these cell lines express the different TET1 versions from the endogenous loci and do not change throughout the cell cycle, an asynchronous population of cells showed consistent data.

In addition to the analysis described above, we performed bisulfite and Tet-assisted bisulfite (TAB) sequencing experiments in these loci in order to investigate the 5mC and 5hmC levels at base resolution level. Bisulfite sequencing itself cannot differentiate 5mC from 5hmC, as both resist deamination during the treatment of DNA with sodium bisulfite (Fig. 9C)^68^. However, we can distinguish between 5mC and 5hmC combining bisulfite sequencing with two additional steps: protection of 5hmC through glucosylation and Tet1-CD mediated oxidation of 5mC to 5caC. After subsequent bisulfite conversion, the protected β-glucosyl-5-hydroxymethylcytosine (5ghmC) is read as C in the sequence, whereas 5caC and C are read as T, enabling single-base resolution sequencing of 5hmC^69^. 5fC can also be oxidized by Tet proteins to 5caC^70^. Thus, only protected 5ghmC will read as C in TAB-sequencing while in bisulfite sequencing both 5mC and 5hmC are read as C (Fig. 9D). Firstly, we verified the activity of purified Tet1-CD used for these experiments by an oxidation reaction with genomic DNA (gDNA) of MCF10a and MCF7 cells. Afterwards, we performed slot blotting of the DNA incubating with antibodies against 5hmC and 5caC. Compared with non-treated gDNA samples, where MCF7 gDNA showed higher levels of 5hmC than MCF10a, after Tet1-CD oxidation reaction, 5hmC levels were reduced while 5caC levels were increased in both cell lines. The same amount of DNA, confirmed by methylene blue staining, was loaded for all samples (Fig. 9E). Bisulfite sequencing analysis of cytosines in the LINE 1 5´UTR promoter (Fig. 9F and Supplementary Fig. 8A) showed strong methylation and hydroxymethylation for all MCF10a and MCF7 cell lines with the exception of the TET1 KO/TET1s-K852R mutants. On the other hand, for the Alu repeats, MCF10a and MCF7 cell lines showed low percentage of methylation and hydroxymethylation, in line with previous results (Fig. 9B). For TAB-sequencing experiments, which analyzed exclusively 5hmC levels in these loci, MCF7 wild-type cells and TET1 KO showed higher levels of hydroxymethylated cytosines for the heterochromatic region selected, compared with MCF10a and MCF7 TET1/TET1s KO. Interestingly, MCF7 TET1 KO/TET1s-K852R cell line showed a reduction of the percent of hydroxymethylation compared with MCF7 and TET1 KO cell lines (Fig. 9G and Supplementary Fig. 8B). However, only MCF7 TET1 KO/TET1s-K852R showed high levels of hydroxymethylated C for the euchromatic region analyzed, the Alu repeat. In conclusion, MCF10a and MCF7 TET1/TET1s KO cells, both with low to no level of TET1 isoforms, showed higher levels of methylated C for the heterochromatic loci. On the other hand, for the euchromatic loci only MCF7 TET1 KO and MCF7 TET1/TET1s KO showed a high percent of methylation, in line with previous results (Fig. 9B).

Altogether, the combination of global genomic DNA and sequence specific eu- versus heterochromatic loci analyses highlights the very different functions of the two TET1 isoforms based on their cell cycle dependent subnuclear targeting.

## Discussion

**Fig. 10:**
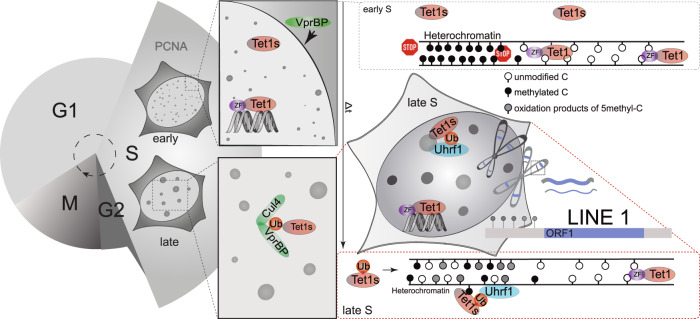
Model of Tet1s and Tet1 regulation during the cell cycle. Tet1 via its zinc finger domain is tethered away from heterochromatin, which prevents spreading of hydroxymethylation to these regions. The short isoform Tet1s lacking this domain gets ubiquitinated by the CRL4(VprBP) complex and the modified protein is bound by Uhrf1 and recruited to late-replicating heterochromatin. This targets Tet1s activity to heterochromatin and results in aberrant oxidation of methylcytosine in heterochromatic regions in both human and mouse cells depending on the level of this isoform. This in turn also results in the reactivation of silenced repetitive DNA elements like LINE 1 or major satellite repeats.

In this study, we describe the recruitment of the Tet1s isoform to heterochromatic regions during ongoing DNA replication, the consequent spreading of 5hmC to heterochromatic regions of the genome with activation of LINE 1 elements and chromatin decondensation, and we elucidate the dual mechanism underlying this spatio-temporally directed Tet1 catalytic activity (Fig. 10).

The subnuclear association of Tet1s at heterochromatin was observed in non-tumorigenic and breast cancer human cell lines, and also in murine cells. This finding points to a conserved molecular mechanism for Tet1s recruitment that is not affected by the malignant transformation. However, cancer cells are in general hypomethylated^71^. In agreement with this, we found that MCF7 breast cancer cells show a significant reduction of 5mC and increased 5hmC levels at heterochromatic regions, compared to non-tumorigenic mammary gland cells, and we showed that MCF7 cells overexpress Tet1s. Non-tumorigenic MCF10a mammary gland cells, on the other hand, expressed only minor levels of Tet1 isoforms. Considering the recruitment and targeted catalytic activity of Tet1s in heterochromatin, the 5mC reduction in MCF7 cells could thus be accounted for by the high Tet1s level in these cells. Nonetheless, some of the changes on global DNA methylation levels could also be attributed to DNA modification fluctuations between individuals, as the MCF7 and MCF10a cells were obtained from two different donors^72^. As about 5% of all cytosines in the human genome are estimated to be methylated, of which a large number is found in a heterochromatic CpG context^73^, the decrease of about 50% in methylation levels we measured, accounts for a considerable number of cytosines that are unlikely to be explained simply by variation between individuals. Comparatively, the minor changes on 5fC levels we measured could be attributed to this modification being an intermediate product of the 5mC oxidation cascade. Additionally, 5fC and 5caC can be excised by the DNA repair enzyme thymine-DNA glycosylase (TDG), followed by replacement with unmodified cytosine^74^. Overall, the observed 5mC reduction, especially in heterochromatin, is supported by our findings in murine cells. In this model, Tet1s overexpression at levels mimicking the ones in MCF7 cells and its targeted catalytic activity to heterochromatin, resulted in a significant 5mC oxidation, and increased 5hmC production.

We expanded the investigation of the cell cycle-dependent nuclear localization of the Tet protein family and found late S-phase recruitment only for Tet1s, but not for Tet1, Tet2 or Tet3. A structural feature that separates these proteins from one another is their zinc finger domain, while they share a conserved C-terminal catalytic domain^60^. Indeed, only constructs lacking the zinc finger domain, not present in Tet1s, were able to show late S-phase accumulation and the zinc finger added to Tet1-CD prevented it. This indicates that the zinc finger is the domain responsible for tethering Tet1 away from heterochromatin. Previous studies have shown that the zinc finger of Tet1 mostly binds non-modified DNA^14^ and is implicated in preventing DNA methylation spreading in euchromatic regions^15^. Additionally, the zinc finger and the BC domains were found to concomitantly increase the DNA binding ability of Tet1 and their deletion was shown to result in decreased chromatin loading^16^. In agreement with that, we found that the zinc finger domain decreases the mobility of the protein by increasing its DNA binding ability, and this diminishes its heterochromatin recruitment and 5hmC spreading into these genomic regions. In the absence of the zinc finger domain in Tet1s isoform, a separate mechanism independent of catalytic activity targets this isoform to heterochromatic regions during late S-phase.

A previous study found Tet1 to interact with PCNA, which was shown to mediate Tet1 activity^59^. The major difference between the putative PBD of Tet1 and the PBD consensus sequence, is a hydrophilic lysine that replaces the central hydrophobic amino acid residue in canonical PBDs^75^. On the one hand, early S-phase cells did not show Tet1s accumulation at replication foci. On the other hand, the putative PBD did not recruit Tet1s to late S-phase replicating heterochromatin. Hence, Tet1s heterochromatin accumulation during late S-phase is likely not dependent on its interaction with PCNA. Nonetheless, aphidicolin treatment, which inhibits DNA polymerase activity, displaced Tet1s from heterochromatin regions concomitant with PCNA dissociation, showing that Tet1s recruitment is replication dependent. It would be interesting to back mutate the putative PBD and analyze the consequences for genomic distribution of DNA modifications. A successful reactivation of the putative PBD, and therefore a restored interaction with PCNA, would hint to an initial function that may have been lost during evolution or replaced by a different mode of recruitment. A PCNA and Uhrf1-dependent mode of recruitment has evolved and been preserved in Dnmt1, which is the enzyme responsible for the maintenance of DNA methylation^7,76^.

Uhrf1 is a ubiquitin ligase, has a multimodal heterochromatin association during S-phase, plays a crucial role in DNA methylation maintenance, contributes to global hypomethylation in the cancer context and targets the DNA de novo methyltransferase Dnmt3A for ubiquitin-dependent proteasomal degradation. Additionally, it was found to be frequently overexpressed in cancer^77^. Remarkably, we found that Uhrf1 physically interacts with Tet1s and is necessary for its recruitment to constitutive heterochromatic regions, which was mediated by the SRA domain of Uhrf1. Interestingly, though, Uhrf1 did not ubiquitinate Tet1s. Altogether, this supports the essential role of Uhrf1 in Tet1s accumulation at replicating heterochromatin, though, independent of Uhrf1 ubiquitination activity.

Fine mapping the domain required for S-phase localization of Tet1s, yielded that the CRD is needed not only for catalytic activity but also for the late S-phase localization, while the DSBH domain is not required. Within the CRD, a conserved lysine was crucial for the S-phase localization of Tet1s as this was abrogated upon mutating the lysine to arginine (Tet1s-K852R) or glutamate (Tet1s-K852E). Interestingly, mutations in the CRD of Tet2 have been found in leukemia patients^78^. In view of the fact that we did not find Tet2 to associate with replicating heterochromatin, we speculate that the effect of such mutations may be rather by affecting its catalytic activity.

Previous studies showed that lysine 852 within the CRD of all Tet proteins is monoubiquitinated by CRL4(VprBP) and modulates Tet catalytic activity and Tet2 chromatin binding^19,20^. Furthermore, the short amino acid stretch harboring the conserved lysine, was shown to stabilize the DNA adjacent to the modified cytosine target, by interacting with the phosphate backbone. Mutations of the conserved lysine residue to a glutamate resulted in loss of Tet2 catalytic activity, indicating its importance for the correct function of Tet proteins^60^. We made similar observations for Tet1s by mutating lysine 852 to a glutamate or an arginine, respectively, and also by inhibiting or down regulating CRL4(VprBP). In this way, Tet1s ubiquitination at lysine 852 is abrogated and its recruitment to replicating heterochromatin abolished. Taken together, this clearly hints to Tet1s being ubiquitinated at lysine 852 by CRL4(VprBP), which in turn regulates Tet1s recruitment to replicating heterochromatin. Both Tet1-CD and Tet1-ZF-CD are able to interact with Cul4 and VprBP, and consequently both could be ubiquitinated, but fusing the zinc finger domain to Tet1-CD is sufficient to prevent its recruitment to replicating heterochromatin by tethering it away from heterochromatin. Incidentally, fusions of *MLL* with *TET1* were reported in leukemia patients^79^. The location of the genomic breakpoints were mapped to *TET1* intron 8, exons 9 and 12. This would retain part of the catalytic domain of TET1 but exclude the CRD domain and, hence, likely, the regulation of TET1 S-phase recruitment reported here will not play a role.

The proteins of the Tet dioxygenase family have been implicated in crucial developmental processes, disease and in different cancer types. Here, we propose a regulation mechanism where Uhrf1 plays an essential role by interacting with Tet1s exclusively during late S-phase, after the ubiquitination of a conserved lysine in the CRD by the CRL4(VprBP) complex. Figure 10 graphically summarizes our model for the multistep recruitment of Tet1s to heterochromatin during late S-phase. We propose that VprBP acts as a substrate recognition component of the E3 ubiquitin-protein ligase complex, in this case, a substrate-specific adapter of DDB1-CUL4, which mediates ubiquitination of Tet1s. Ubiquitination of Tet1s at the conserved lysine residue in the CRD, in turn, allows interaction with Uhrf1 and consequently recruitment to heterochromatin during late S-phase. This is followed by oxidation of the very abundant methylcytosines within these chromatin regions. The zinc finger domain in the full-length Tet1 protein, on the other hand, keeps the enzyme tethered away from heterochromatin. As functional consequences of the recruitment of the Tet1s isoform to heterochromatic regions and the consequent spreading of 5hmC to heterochromatin, decondensation of heterochromatin takes place accompanied by activation of LINE 1. Such long interspersed DNA repeat elements are highly abundant in human cells^30^, are enriched at heterochromatin^34^, known to be activated in cancer cells^80–83^ as well as in early development^84^ and are normally kept silenced by DNA methylation^85^ and methylcytosine binding proteins^37,43^.

The reexpression of LINE 1 and satellite repeats is a commonly observed feature of many epithelial cancer types and also observed in clonal hematopoiesis and AML^86,87^. Moreover, genomic insertions upon LINE 1 reactivation are found to be disruptive to genes, where they are newly inserted, and this can render tumor suppressor genes inactive. However, Tet1s recruitment to highly methylated heterochromatin suggests a mechanism, relying on the targeted catalytic activity of Tet1s and the reactivation of retrotransposable elements. The finding that Tet1s is overexpressed in many cancer types^17^ makes a co-overexpression of Uhrf1 and Tet1s a likely scenario. Speculating on the biological significance of Tet1s targeting, we could propose a model where the loss of DNA methylation in heterochromatin plays a crucial role. Given the proposed role of full-length Tet1 as a tumor suppressor^88,89^, our results suggest a tumor promoting role for Tet1s by drastically changing the epigenetic landscape of a cell. Importantly, the (patho)physiological outcome is not exclusive to cancer cells but is regulated by the abundance of the Tet1 isoforms and their stoichiometry.

## Methods

### Experimental model

We used the following cell lines:

– MCF10a (non-tumorigenic) and MCF7 (cancer), human cell lines.
– MCF7 (TET1 KO), MCF7 (TET1/TET1s KO), MCF7 (TET1 KO/TET1s-K852R).
– C2C12 mouse myoblast.
– Mouse embryonic fibroblast MEF-PM (Dnmt1^−/−^, p53^−/−^) and MEF-P (p53^−/−^).
– Mouse embryonic stem E14 wild-type cells and Uhrf1-deficient cells (Uhrf1^−/−^).
– HEK293-EBNA human cell line.

All the references are given and details are described in Supplementary Table 1.

### Cell culture, transfection and treatments

C2C12 mouse myoblasts^90^ were cultured in Dulbecco’s Modified Eagle’s Medium (DMEM) (Sigma-Aldrich Chemie GmbH, Steinheim, Germany; Cat.No.: D6429) containing 20% FCS. Mouse embryonic fibroblasts (MEF) deficient for Dnmt1 and p53 (MEF-PM, Dnmt1^−/−^, p53^−/−^), deficient only for p53 (MEF-P, p53^−/−^)^49^ were cultured in DMEM containing 15% or 10% FCS, respectively^44^. Female breast epithelial cells MCF10a were cultured in DMEM/F12 (Sigma- Aldrich Chemie GmbH, Steinheim, Germany; Cat.No.: D8900) supplemented with final concentrations of 5% horse serum (Sigma-Aldrich Chemie GmbH, Steinheim, Germany; Cat.No.: H0146), 20 ng/mL EGF (Sigma-Aldrich Chemie GmbH, Steinheim, Germany; Cat.No.: E9644), 0.5 mg/mL hydrocortisone (Sigma-Aldrich Chemie GmbH, Steinheim, Germany; Cat.No.: H0888), 100 ng/mL cholera toxin (Sigma-Aldrich Chemie GmbH, Stein- heim, Germany; Cat.No.: C8052) and 10 μg/mL insulin (Sigma-Aldrich Chemie GmbH, Steinheim, Germany; Cat.No.: I2643), and the breast cancer epithelial cells MCF7 were cultured in DMEM (Sigma-Aldrich Chemie GmbH, Steinheim, Germany; Cat.No.: D6429) containing 10% FCS, as described before^91^. Mouse embryonic E14 wild-type stem cells and therefrom derived Uhrf1-deficient cells (Uhrf1^−/−^)^48^ were cultured under feeder-free, 2i/LIF conditions^37,43^ in culture dishes that were coated with 0.2 % gelatin (Sigma-Aldrich Chemie GmbH, Steinheim, Germany, Cat.No.: G2500). DMEM (Sigma-Aldrich Chemie GmbH, Steinheim, Germany; Cat.No.: D6429) for embryonic stem cell culture contained 16% FCS and was, in addition to L-glutamine and Pen/Strep, supplemented with 1x non-essential amino acids (Sigma-Aldrich Chemie GmbH, Steinheim, Germany; Cat.No.: M7145), 0.1 mM β-mercaptoethanol (Carl Roth, Karlsruhe, Germany, Cat.No.: 4227), 0.1 μM PD 0325901 (Axon Medchem BV, Groningen, The Netherlands, Cat.No.: Axon 1408), 0.3 μM CHiR 99021 (Axon Medchem BV, Groningen, The Netherlands, Cat.No.: Axon 1386) and 1,000 U/ml LIF (Enzo Life Sciences GmbH, Lörrach, Germany, Cat.No.: ALX-201-242). HEK293-EBNA human embryonic kidney cells (Invitrogen; catalog # 620-07, Paisley PA49RF, UK) were cultured in DMEM (Sigma-Aldrich Chemie GmbH, Steinheim, Germany; Cat.No.: D6429) containing 10% FCS. All cell lines were regularly tested for mycoplasma to ensure that they were contamination free.

C2C12, MEF, MCF7 and MCF10a cells were transfected by electroporation with the AMAXA Nucleofector II system (Lonza, Cologne, Germany), using a self-made buffer (5 mM KCl, 15 mM MgCl2, 120 mM Na2HPO4/NaH2PO4 pH 7.2, 50 mM Mannitol)^92^ with default programs B032, A024, P020 or T024, respectively. Mouse embryonic stem cells were transfected with the Neon electroporation system (ThermoFisher scientific) according to the manufacturer’s instructions. HEK293-EBNA cells were transfected with polyethyleneimine (PEI, Sigma-Aldrich) as previously described^93^.

To assess the effects of replication fork stalling on the accumulation on Tet1s or Tet1-CD, C2C12 cells were transfected with mcherry-Tet1s or Tet1-CD and miRFP-PCNA and subjected to live-cell time lapse microscopy at 5 min time intervals. After three time points, 50 µg/mL aphidicolin (Sigma-Aldrich, St Louis, MO, USA, Cat.No.: A0781), which stalls the replication machinery^55^, or DMSO (dimethyl sulfoxide from Sigma-Aldrich, St Louis, MO, USA, Cat.No.: 41639) were added to the cells and imaging was continued for 30 min at the same intervals.

To test the effects of the NEDD8 8 activating enzyme (NAE)-inhibitor pevonedistat that prevents the neddylation-dependent activation of Cul-family ubiquitin ligases^65^ (MLN4924, MedChemExpress Europe, Sollentuna, Sweden, Cat.No.: HY-70062) on the recruitment of Tet1s, C2C12 were transfected with plasmids encoding fluorescently tagged PCNA and Tet1s and 8 h later treated with 10 µM pevonedistat or DMSO and after 5 h of additional incubation subjected to live-cell microscopy.

Dependence of 5hmC levels on Cul4 activity was addressed by treating cells with 3 µM pevonedistat or DMSO for 24 h, followed by fixation and 5hmC immunostaining.

For ubiquitination assays, transfected HEK293-EBNA cells were treated with 3 µM pevonedistat or DMSO for 24 h. When using endogenous ubiquitin, HEK293-EBNA cells were treated with 10 µM of proteasome inhibitor MG-132 (MedChemExpress Europe, Sollentuna, Sweden, Cat. No.: HY-13259) or DMSO for 24 h.

To enrich the protein lysate in cells at the S-phase stage, synchronization of MCF7, MCF10a, and C2C12 cells was performed by double thymidine arrest^94^ (Sigma-Aldrich) to a final concentration of 2 mM. Thymidine is used to synchronize the cells in G1/early S-phase. MCF7 and MCF10a cells were processed for co-immunoprecipitation 5 h after release into normal growth medium. For chromatin immunoprecipitation (ChIP) experiments, C2C12 were processed just after release (in G1/early S-phase) or 7 h after release into normal medium to enrich the sample in cells at the late S-phase.

### CRISPR/Cas9‑mediated genomic engineering of TET1/TET1s and TET1s-K852R mutant generation in MCF7 cells

For the generation of TET1, TET1/TET1s knockouts, and TET1s-K852R point mutation, specific gRNAs (Supplementary Table 4, Supplementary Fig. 7) were cloned into a puromycin-selectable vector expressing both SpCas9 and gRNA (px459: F. Zhang laboratory). CRISPR gRNAs were designed using http://crispr.mit.edu/. MCF7 cells were transfected with Cas9-gRNA vectors and the homologous recombination template (for TET1s-K852R point mutation) using the Neon electroporation system (ThermoFisher scientific) according to the manufacturer’s instructions. Two days after transfection, cells were plated at clonal density in DMEM media supplemented with 2 μg/mL puromycin (Gibco). Selection media was removed after 48 h, replaced with normal ESC media, and colonies were allowed to grow for an additional 4–5 days. Single colonies were transferred into 96-well plates and the plates were duplicated after 2 days. Enrichment for mutated clones was accomplished by amplifying the CRISPR/Cas targeted region via PCR (oligonucleotides in Supplementary Table 4). Cell lysis in 96-well plates and PCR on lysates were performed as previously described^95^. and analyzed on 1.5% agarose gels. PCRs of positive clones were confirmed by Sanger sequencing, and sequence alignment was performed with Clustal Omega (Supplementary Table 7). Clones harboring biallelic mutations were then assessed for loss of TET1 and/or TET1s via Western blot and immunofluorescence analysis against using monoclonal antibodies (rat anti-TET1, Supplementary Table 5) as described before.

### Expression constructs

Mammalian expression plasmids encoding GFP- (pc0653), mRFP-tagged PCNA (pc1054) and miRFP-tagged PCNA (pc3385), have been described in previous works. A GFP-tagged construct encoding the short isoform of Tet1 (pc3901) was generated by amplifying the respective fragment from full-length Tet1 (pc2271) and replacing the full-length Tet1 sequence with the Tet1s sequence by restriction with AsiSI and NotI. Mcherry-tagged Tet1s (pc3905) was generated by replacing the sequence of Uhrf1 (pc1756) with the Tet1s sequence just described using AsiSI and NotI restriction enzymes. GFP- or mcherry-tagged catalytically active and inactive Tet1 catalytic domain (Tet1-CD) constructs (pc2315, pc2547, pc2815, pc2637) as well as mcherry-tagged catalytic domains of Tet2 and Tet3 (pc3338, pc3339) and GFP-tagged Tet1, 2 and 3 full-length (pc2271, pc2272, pc2273) constructs have been described before. The sequences encoding the CRD of GFP-Tet1s (pc3904), as well as the amino acids 1–389 (pc3174) and 566-833 (pc3175) of full-length Tet1 were deleted by overlap-extension PCR^96^ and the amplicon obtained was used to replace the Tet1 coding sequence in EGFP-Tet1 (pc2271). GFP-tagged Tet1-CRD (pc2334) and Tet1-DSBH (pc2335) were generated by amplifying the respective sequences from full-length Tet1 and replacing the full-length Tet1 sequence by AsiSI and NotI restriction. To obtain a GFP-tagged PBD encoding the putative Tet1-PBD (pc3918), oligo cloning was performed and the DNMT1-PBD in a pEGFP-N2 backbone (pc0883) was replaced by XmaI and EcoRI restrictions. A mcherry-tagged Tet1-ZF-CD fusion (pc3956) was constructed by PCR-amplifying the Tet1-ZF domain including a linker sequence from GFP-Tet1-ZF and inserting the amplicon in the mcherry-Tet1-CD backbone by AsiSI restriction. To mutate lysine 852 in Tet1s to glutamate (pc3915) or arginine (pc3914), a sequence- and ligation-independent cloning approach was chosen^97^.

GFP- or mcherry-tagged full-length Uhrf1/Uhrf2 (pc1709, pc1756, pc1976) and Uhrf1 single domain deletions (pc1933, pc1934, pc1935, pc2164, pc2987) or single domains fused to GFP (pc1936, pc1937, pc1938, pc3061, pc3063) were described in previous studies, as well as GFP-tagged DNMT1 construct (pc1099).

Mcherry-tagged VprBP (pc2954) was cloned from murine ESC cDNA by overlap-extension PCR^96^ to replace Uhrf1 (pc1756) using AsiSI and NotI restriction enzyme sites. To obtain an GFP-tagged VprBP (pc2953), the VprBP coding sequence was excised from the vector just described and used to replace the TDG sequence in GFP-TDG (pc2422). For VprBP knockdown, a lentiviral vector encoding a *VprBP* siRNA (5’- CCAGATCGTGTGTTTGTTGAGCTGTCTAA-3’) under a U6 promoter and an in frame GFP under a CMV promoter was obtained from abmgood (pc3922).

Plasmids encoding a hemagglutinin-tagged ubiquitin and a major satellite repeat recognizing polydactyl zinc finger (MaSat) fused to GFP (pc1803) or a GFP-recognizing nanobody (pc2469) were described in previous publications. To create a miRFP-tagged MaSat (pc3944), pmiRFP670-N1 (pc3379)) was used and MaSat-GFP (pc1803) was cut with SacI and AgeI and fused with miRFP670.

Details and references of all plasmids and oligonucleotides used in the cloning are shown in Supplementary Tables 2, 3. Additionally, schemes for the main Tet-X constructs are shown in Supplementary Fig. 9. SerialCloner (Version 2.6.1) was used for plasmids design and all constructs were verified by DNA sequencing.

### Live-cell microscopy and image analysis

Live-cell time lapse, live-cell imaging and fluorescence recovery after photobleaching (FRAP) experiments were performed with a Nikon T*i*-E microscope equipped with an UltraVIEW VoX spinning disk confocal unit (PerkinElmer, UK), controlled by Volocity 6.3 software (PerkinElmer, UK), and equipped with a live-cell chamber (ACU control, Olympus) set at 37 °C with 5% CO_2_ and 60% air humidity. Z-stacks were acquired with a ×60/1.49 NA CFI Apochromat TIRF oil immersion objective (voxel size, 0.12 × 0.12 × 0.3–1 µm; Nikon, Tokyo, Japan) or a 100x/1.49 NA CFI Apochromat TIRF oil immersion objective (voxel size, 0.071 × 0.071 × 0.5–1 µm; Nikon, Tokyo, Japan) and a cooled 14-bit CCD camera (Hamamatsu Photonics K.K., Hamamatsu City, Japan, Cat.No.: C9100-50). Z-stack images were analyzed using Volocity 6.3 (PerkinElmer, UK) and Fiji. Mid Z-planes were assembled onto videos and annotated using Fiji^98^ (https://Fiji.nih.gov/ij/).

#### Protein accumulation at heterochromatin analysis

Heterochromatin accumulation ability of ectopically expressed, fluorescently tagged proteins, during ongoing DNA replication, was assessed by transfecting cells with the respective plasmids and imaging cells live 8–12 h post-transfection. Confocal Z-stacks (voxel size, 0.12 × 0.12 × 0.5 µm) were acquired using the aforementioned Nikon-T*i*-E setup. Z-stacks were analyzed using Fiji with a self-written semi-automated macro routine (macro 1). For this purpose, Z-stack images were converted to maximum Z-projections. After this step, three circular regions with a radius of 4 pixels were chosen in the nucleoplasm of each cell, and correspondingly, three circular same-sized regions in heterochromatin marked by PCNA, were measured. The ratio of the averaged signal intensities of the protein of interest in PCNA marked pericentric heterochromatin and in the nucleoplasm was plotted with RStudio (Version 1.1.447)^99^ as relative accumulation values at replicating heterochromatin.

#### Fluorescence recovery after photobleaching

For FRAP experiments, C2C12 cells were transfected by electroporation 8 h prior to the experiments. FRAP analysis was essentially performed as described before (33). Briefly, spots were chosen in nuclei of late-replicating cells or non-replicating cells and bleached for 600 ms with a 488 nm laser for GFP-tagged constructs (Fig. 5H) or 1 s with a 561 nm laser for mcherry-tagged proteins (Fig. 3D), both set to 100%. In late-replicating cells, replication foci marked by PCNA or regions without PCNA accumulation were bleached. For analysis, raw intensities of the bleached area (ROI1), a non-bleached nuclear area (ROI2), and a background area outside the cell (ROI3) as well as the corresponding time points were calculated using a custom Fiji macro^100^. Quantitative evaluation was performed using Fiji, and fluorescence intensity normalization and curve fitting were performed with the easyFRAP software (https://easyfrap.vmnet.upatras.gr/) as described before^92^ using the double normalization method and the double term equation for the fitting procedure^101^. Briefly, the mean intensity of the bleached region (ROI1) was divided by the mean intensity of the ROI2 and both intensities were corrected for the background levels (ROI3). T-half values were extracted from the mean exponential fitting, and plots were generated with RStudio (Version 1.2.1335).

#### Fluorescent three-hybrid (F3H) assay

To address the effect of targeting potential effector proteins to major satellite repeats on Tet1s/Tet1-CD recruitment to heterochromatin, a previously described fluorescent three-hybrid assay was adapted^102^. To this end, C2C12 cells were transfected with plasmids encoding GBP-MaSat, GFP-tagged Uhrf1 or Uhrf1 single domains or Uhrf1 single domain deletions and mcherry-tagged Tet1-CD (Fig. 4G) and cells without GBP-MaSat where used as control. Cells were imaged live 8–12 h post-transfection and confocal Z-stacks (voxel size, 0.12 × 0.12 × 0.5 µm) were acquired using the aforementioned Nikon-T*i*-E setup. Z-stacks were analyzed and mounted using Fiji. For quantification purposes, the percentage of cells showing colocalization of GFP-tagged Uhrf1-X and mcherry-tagged Tet1-CD in each S-phase stage was calculated with a minimum number of 50 cells per sample.

Details about imaging systems, software and Fiji macros are shown in Supplementary Tables 6, 7.

### Immunofluorescence, microscopy and image analysis

For immunofluorescent staining of modified nucleotides in cells, Tet1 or replication associated proteins, cells were seeded on gelatin-coated glass coverslips and fixed in 3.7% formaldehyde (Sigma-Aldrich Chemie GmbH, Steinheim, Germany, Cat.No.: F8775) in 1x PBS for 10 min. After three washing steps with PBS-T (1x PBS, 0.01% Tween-20), cells were permeabilized with 0.5% Triton X-100 in 1x PBS for 20 min, incubated in ice-cold 88% methanol for 5 min and washed again.

For the staining of modified nucleotides, cells were incubated with 10 mg/mL RNaseA in 1x PBS for 30 min at 37 °C. After three more washing steps, cells were blocked with 1% BSA in 1x PBS at 37 °C for 30 min. The primary antibody solution contained a final concentration of 0.5% BSA, 1x DNaseI reaction buffer (60 mM Tris/HCl pH 8.1, 0.66 mM MgCl_2_, 1 mM β-mercaptoethanol) and 0.1 U/mL DNaseI (Sigma-Aldrich Chemie GmbH, Steinheim, Germany, Cat.No.: D5025). In addition, another protocol for DNA denaturation was performed and compared with the DNaseI treatment: the cells were incubated with 4 N HCl for 15 min at room temperature (RT), rinsed with distilled water, and placed in 100 mM Tris–HCl (pH 8.5) for 10 min. Then, cells were washed with PBS again. The primary antibody mix was incubated at 37 °C for 70 min and afterwards washed three times with PBS-TE (PBS-T + 100 mM EDTA). Cells that were immunostained against Tet1, PCNA and VprBP were blocked in 1% BSA for 30 min after fixation and incubated with primary antibodies diluted in 1% BSA for 60 min at room temperature. Antibodies against 5mC^103^ and 5hmC, 5fC or 5caC (all Active Motif, La Hulpe, Belgium), as well as characteristics of antibodies against Tet1^10^, PCNA and secondary antibodies, as well as dilutions used, are described in Supplementary Table 5. After incubation with the primary antibodies, cells were washed three times with PBS-T. For the detection of the primary antibodies, cells were incubated with fluorescently tagged secondary antibodies diluted in 1% BSA. Alexa Fluor 488-conjugated goat anti-rabbit IgG (H + L) (1:500), Alexa Fluor 488-conjugated goat anti-mouse IgG (H + L) (1:500), Alexa Fluor 594-conjugated goat anti-rabbit IgG (H + L) (1:250; ThermoFisher Scientific, Invitrogen, Carlsbad CA, USA, Cat.No.: R37117), Cy5-conjugated donkey anti-mouse IgG (H + L) (1:250; The Jackson Laboratory, Bar Harbor, ME, USA, Cat.No.: 715-715-150). After an incubation of 45 min at room temperature, cells were washed three times with PBS-T, counterstained with 10 mg/ml DAPI (4’,6-diamidino-2-phenylindole, Sigma-Aldrich Chemie GmbH, Steinheim, Germany, Cat.No.: D9542) and mounted in Mowiol® 4-88 (Sigma-Aldrich Chemie GmbH, Steinheim, Germany, Cat.No.: 81381).

#### In situ quantification of cytosine modification levels

To address the endogenous levels of 5mC, 5hmC, 5fC and 5caC in heterochromatic domains of MCF10a or MCF7 cells, cells were immunostained against 5mC and 5hmC, 5fC or 5caC and counterstained with DAPI (Fig. 1B). Confocal Z-stacks with a Z-step length of 0.5 µm were acquired with the described Nikon T*i*-E spinning disk setup: maximum Z-projections were generated and circular ROIs with a radius of 5 pixels were created around DAPI-dense regions (Supplementary Fig. 1B). To quantify cytosine modifications levels in pericentric heterochromatin and the nucleoplasm, C2C12 cells were transfected with GFP/mcherry-tagged Tet1-variants or GFP (pc0713) alone and immunostained against 5hmC, 5fC or 5caC and counterstained with DAPI. Cells were imaged with the before described confocal spinning disk setup and analyzed with Fiji. For both MCF and C2C12 cells, images were analyzed with a self-written semi-automated macro routine for Fiji (macro 3). In brief, maximum Z-projections were generated and binary nuclear masks were created of the respective 16-bit images, based on the DAPI/5mC signal. For this, images were thresholded with the triangle method and gray values above 3000 were considered for the mask. A second binary mask for C2C12 or MCF heterochromatin was created with the triangle threshold method for all pixels with intensities above 7000. Based on these two masks a third mask for the nucleoplasm was calculated and the respective mean cytosine modification levels were measured and plotted with RStudio (Version 1.1.447).

#### High-content microscopy

Endogenous Tet1/Tet1s, 5mC and 5hmC levels and levels of ectopically expressed GFP- or mcherry-tagged proteins were measured with the Operetta high-content screening system (Perkin Elmer, UK) in wide-field mode, equipped with a Xenon fiber optic light source and a 20x/0.45 NA long working distance or a ×40/0.95 NA objective. For excitation and emission, following filter combinations were used, 360-400 nm and 410-480 nm for DAPI or AMCA, 460-490 nm and 500–550 nm for GFP or Alexa-488 as well as 560–580 nm and 590–640 nm for TexasRed or Alexa-594. Fluorescence intensity levels were quantified with the Harmony software (Version 3.5.1, PerkinElmer, UK) (Supplementary Table 7). For the analysis of cells that were transfected with GFP or mcherry fusion encoding plasmids and stained against 5mC, 5hmC, 5fC, 5caC or Tet1/Tet1s and counterstained with DAPI, cell nuclei were first identified according to their GFP/mcherry fluorescence and evaluated for morphological properties like roundness and size. These parameters as well as the mean and sum nuclear fluorescence intensities of DAPI, GFP/mcherry and Alexa-594/488-labeled antibodies were calculated in cells fitting these morphological criteria. For further analysis of cytosine modifications levels, sum nuclear Alexa-594/488 intensities were divided by the sum nuclear DAPI intensity, to compensate for potential cell cycle-dependent fluctuations and these normalized values were grouped according to the mean GFP/mcherry intensities of the respective cells. GFP levels below 50 arb. units (Arbitrary Units) and mcherry levels below 100 arb. units were considered as background. The Alexa values of cells above the background level were normalized by dividing the respective values by the averaged intensity of cells below the background levels. For further analysis of Tet1/Tet1s levels, sum nuclear Alexa intensities in MCF7, MCF10a and C2C12 cells were divided by the average sum nuclear Alexa intensity in non-transfected C2C12 (showing the lowest levels of Tet proteins). In all immunofluorescence experiments, cells above GFP or mcherry background levels were grouped in different expression level groups according to their mean GFP or mcherry fluorescence levels: low (50-100 AU), mid (100-500 AU), and high (500-1000 AU) for GFP, and low (100-500 AU), mid (500-1000 AU), and high (1000-5000 AU) for mcherry (Supplementary Fig. 2C). The nuclei of MCF10a and MCF7 cells that were simultaneously stained for 5mC and 5hmC, 5fC or 5caC and counterstained with DAPI, were identified by their DAPI staining and further grouped according to morphological criteria. The mean and sum nuclear fluorescence intensities of DAPI, Alexa-488-labeled 5mC and Alexa-594 labeled 5hmC were calculated in cells fitting these criteria. For normalization, the sum nuclear Alexa-488 and Alexa-594 levels of cells that were only stained with the secondary antibodies, were averaged to obtain a background fluorescence value. The sum nuclear fluorescence values of Alexa-488/594 in cells that were incubated with primary and secondary antibody were each normalized to the respective average background fluorescence and further normalized to the DAPI fluorescence intensity. Exported measurement results were further analyzed and plotted with RStudio (Version 1.1.447)^99^.

#### Quantitative analyses of the 3D nuclear landscape

C2C12 cells were transfected with Tet1 or Tet1s, and 12 h after were fixed and immunostained against PCNA. DNA was counterstained with DAPI as described above. Leica SP5 II confocal microscope equipped with an HCX PL APO ×100/1.44 oil objective was used for imaging of Z-stacks and Fiji was used for image processing. RStudio was used for image analysis with Nucim and statistics^61^. We assessed seven different chromatin compaction levels in individual cell nuclei using DAPI as a proxy for local differences in chromatin compaction. The tools are freely available in open-source R packages “nucim” and “bioimagetools”.

Details about imaging systems, software and Fiji macros are shown in Supplementary Tables 6, 7.

### Co-immunoprecipitation and western blotting

Co-immunoprecipitations were essentially performed as described before^104^. In brief, HEK293-EBNA cells growing in 100 mm dishes were PEI-transfected and harvested by centrifugation 48 h later at 90% confluence. The cell pellet was washed with ice-cold 1x PBS and pelleted again. The supernatant was discarded and the pellet resuspended in 200 µL lysis buffer (20 mM Tris-HCl pH 8, 150 mM NaCl, 0.5 mM EDTA, 0.5% NP-40) supplemented with Pepstatin A (1 µM; Sigma-Aldrich, St. Louis, MO, USA), PMSF (10 µM, Sigma-Aldrich, St. Louis, MO, USA) and AEBSF (1 mM, AppliChem, Darmstadt, Germany). Cells were homogenized with a syringe (21 G needles, 20 strokes) and incubated on ice for 30 min with repeated vortexing in between. Lysates were then cleared by centrifugation for 15 min at 13,000*g* and 4 °C. 15% of the lysate was used as input and the rest was incubated with GFP-binder beads produced as described before^53^ on a rotator at 4 °C for 90 min. Afterwards, the beads were washed 3 times with 500 µL washing buffer. Input and bound fraction were boiled at 95 °C in 4x SDS loading buffer (200 mM Tris/HCl pH 6.8, 400 mM DTT, 8% SDS, 0.4% bromophenol blue and 40% glycerol), separated on a 6% SDS-PA (sodium dodecyl sulfate–polyacrylamide) gels and transferred onto nitrocellulose membranes (GE Healthcare, Munich, Germany). Membranes were blocked with 3% low fat milk in 1x PBS for 30 min and subsequently incubated with the primary antibodies diluted in a blocking buffer for 2 h at room temperature. After washing with 1x PBS supplemented with 0.01% Tween-20, the membrane was incubated with the respective secondary antibodies. For the detection of GFP- or RFP-tagged proteins, rat monoclonal anti-GFP (ChromoTek, Planegg-Martinsried, Germany) and rat monoclonal anti-RFP^105^ were used as primary antibodies. HA-tagged ubiquitin used for the ubiquitination assay was detected with the mouse monoclonal antibody anti-HA tag (clone 12CA5) directed against a nonapeptide sequence derived from the influenza hemagglutinin protein^106^. Antibodies details are summarized in Supplementary Table 5.

For western blot and endogenous co-immunoprecipitation, MCF7, MCF10a and C2C12 cells growing in 100 mm dishes were processed for protein extraction and cell lysates were prepared as described above. For endogenous co-immunoprecipitation of Tet1/Tet1s in MCF7 and MCF10a cells were synchronized at late G1/early S, 15% of the cell lysate was used as input and the rest was incubated as with Pierce™ Protein G agarose beads (ThermoFisher Scientific) preincubated with antibodies against Tet1/Tet1s or MIN (attP synthetic peptide) as negative control for immunoprecipitation, for 1 h. For detection of the different proteins, the following primary antibodies were used: rabbit anti-VprBP polyclonal antibody (Proteintech, USA), rabbit anti-Cul4B polyclonal antibody (Sigma-Aldrich), mouse anti-Cul4 (H-11) monoclonal antibody (Santa Cruz Biotechnology) and Tet1/Tet1s were detected with monoclonal rat antibody (clone 4H7)^10^. As secondary antibodies, horseradish peroxidase (HRP) conjugated goat anti-rat IgG (Jackson; West Grove, PA, USA) (1:500), sheep anti-mouse IgG (Amersham Pharmacia Biotech, United Kingdom), and goat anti-rabbit IgG (Sigma-Aldrich, United States) were used (1:5000). To image the membranes the Amersham AI600 Imager was used (GE Healthcare, Chicago, II, USA). Cut-outs of the membranes were made for a better composition of the figures. Details about antibodies and imaging systems are shown in Supplementary Tables 5, 6. Uncropped and unprocessed scans of all of the blots are available in the Source Data file. The full images and replicates are provided with the data sets uploaded to 10.48328/tudatalib-594.3.

### Chromatin immunoprecipitation

C2C12 were transfected with different GFP-tagged constructs and after synchronization were fixed with 1% formaldehyde for 10 min at room temperature. The crosslink was quenched with 125 mM glycine (5 min at room temperature). Nuclei were isolated after mild lysis in hypotonic buffer (10 mM HEPES pH 8, 1.5 mM MgCl2, 60 mM KCl) and 20 strokes in a tight dounce homogenizer. Chromatin was sheared in the sonication buffer (0.5% SDS 10 mM EDTA, 50 mM Tris–HCl pH 8.1). Fragmentation of chromatin was carried out by ultrasound treatment using a Branson 250 Sonifier (4 × 30 sec at 20% power with a 1 min break on ice in between shearings) obtaining a shearing distribution from 1000bp-300bp with most of the DNA concentrated in the 500 bp range. For each transfection, chromatin from one p100 plate at 80% confluency was immunoprecipitated by incubation with GFP-binder beads produced as described before^53^ on a rotator overnight at 4 °C. Afterwards, cell debris were pelleted at 8000*g* and 4 °C for 10 min, and the supernatant was taken as unbound or input fraction. The beads were washed three times and collected by centrifuging at 8000*g* and 4 °C for 5 min. The chromatin collected (ChIP sample) was then reverse-crosslinked in the presence of 200 mM NaCl at 65 °C for at least 5 h, followed by RNase A (50 μg ml^−1^) treatment for 30 min at 37 °C and proteinase K (100 μg ml^−1^) treatment for 3 h at 50 °C. DNA elution was carried out in 1% SDS, 100 mM NaHCO3, in a rotary shaker at room temperature for 30 min. Pure DNA was isolated using the Qiagen PCR purification kit, and was used to perform real-time quantitative polymerase chain reaction of major satellite repeats. The input sample was essentially prepared following the same protocol.

### Genomic DNA preparation

For the preparation of genomic DNA (gDNA), MCF cells, as well as C2C12 mouse myoblast were pelleted (10 min, 200*g*, 4 °C) and incubated overnight at 50 °C in TNES buffer (10 mM Tris; pH 7.5, 400 mM NaCl, 10 mMEDTA, 0.6% SDS) supplemented with 1 mg/ml Proteinase K (Carl Roth, Karlsruhe, Germany)^107^. RNA was removed by the addition of 0.6 mg/ml RNase A (Qiagen, Hilden, Germany) for 30 min at 37 °C. gDNA was extracted by the addition of 6 M NaCl at a final concentration of 1.25 M and vigorous shaking^107^. After centrifugation (15 min, 11,000*g*, RT), gDNA was precipitated from the supernatant by the addition of 100% ice cold ethanol followed by incubation at –20 °C for 1 h and subsequent centrifugation (10 min, 11,000*g*, 4 °C). After a washing step in 70% ethanol, gDNA was air dried and solved in ddH_2_O.

After chromatin immunoprecipitation (ChIP), isolated gDNA from C2C12 cells was fragmented (<2000 bp) by sonication. The concentration of gDNA was measured on a TECAN infinite M200 plate reader (Tecan Group Ltd., Maennedorf, Switzerland).

### Real-time quantitative polymerase chain reaction of major satellite repeats

For C2C12 mouse myoblasts, equal amounts of DNA (0.5 ng) were used for real-time PCR with Platinum SYBR Green qPCR SuperMix-UDG w/ROX (Invitrogen, Paisley PA4 9RF, UK) on a StepOne-Plus Real-Time PCR System (Applied Biosystems, Darmstadt, Germany) according to the manufacturer’s instruction. UDG was inactivated for 2 min at 50 °C and DNA was denatured for 10 min at 95 °C. Cycle parameters were set to 40 cycles of 15 s at 95 °C and 45 s at 60 °C. Specificity of amplification products was confirmed by melting curve analysis. DNA levels were normalized to Gapdh and calculated using the comparative CT method. Primers for quantitative real-time PCR contained the following sequences: Gapdh forward: 5-CCA TACATACAGGTT TCT CCA G-3, Gapdh reverse: 5-CTG GAA AGCTGT GGC GTG ATG G-3,MajSat forward: 5-GGC GAG AAA ACT GAA AAT CAC G-3, MajSat reverse (20): 5-AGG TCC TTC AGT GTG CAT TTC-3^108^.

### UHPLC‑MS/MS analysis of DNA samples

Isolation of genomic DNA was performed according to earlier published work^109^. The amount of DNA was calculated after photometrically determining the DNA concentration (Implen NanoPhotometer, N60, Version NPOS 4.2e build 14900), before being digested to nucleosides by Nucleoside Digestion Mix (M0649S) kit (New England BioLabs Inc.) according to the manufacturer’s instructions. 1 μg of genomic DNA was used, to which we spiked a heavy labeled nucleoside mix. This mix was prepared from heavy labeled nucleosides with the final concentrations of [15N2,D2]-, [D3]-mdC 51.0 pmol, hmdC 7.7 pmol, [15N2]-fdC 0.04557 pmol, [15N2]-cadC 0.04301 pmol and [15N5]-8-oxo-dG 0.109 pmol. A final volume of 50 μL was then incubated at 37 °C for 1.5 h. Before submitting the samples to mass spectrometry, all samples were filtered by using an AcroPrep Advance 96 filter plate 0.2 μm Supor (Pall Life Sciences). The injection volume amounted to 39 μL. Data were processed according to earlier published work^110^. Quantitative UHPLC-MS/MS analysis of the digested DNA samples was performed using an Agilent 1290 UHPLC system equipped with a UV detector and an Agilent 6490 triple quadrupole mass spectrometer. Natural nucleosides were quantified with the stable isotope dilution technique. For the concurrent analysis of all nucleosides in one single analytical run, the source-dependent parameters were as follows: gas temperature 80 °C, gas flow 15 L/min (N2), nebulizer 30 psi, sheath gas heater 275 °C, sheath gas flow 15 L/min (N2), capillary voltage 2,500 V in the positive ion mode, capillary voltage − 2250 V in the negative ion mode and nozzle voltage 500 V. The fragmentor voltage was 380 V/ 250 V. Delta EMV was set to 500 V for the positive mode. Chromatography was performed with a Poroshell 120 SB-C8 column (Agilent, 2.7 μm, 2.1 mm × 150 mm) at 35 °C using a gradient of water and MeCN, each containing 0.0085% (v/v) formic acid, at a flow rate of 0.35 mL/min: 0 → 4 min; 0 → 3.5% (v/v) MeCN; 4 → 6.9 min; 3.5 → 5% MeCN; 6.9 → 7.2 min; 5 → 80% MeCN; 7.2 → 10.5 min; 80% MeCN; 10.5 → 11.3 min; 80 → 0% MeCN; 11.3 → 14 min; 0% MeCN^111^. Concentrations of DNA modifications were calculated using integrated values from ion chromatogram peaks (Supplementary Data 2).

### DNA glucosylation, MspI and HpaII digestion and PCR based 5hmC and 5mC detection (GluMS-PCR)

To detect 5hmC in the different MCF cell lines, gDNA was extracted and measured for concentration and purity as described above. 10 µg of gDNA were treated with or without 0.18 µM of T4 phage b-glucosyltransferase (T4-BGT)^112^ in a final volume of 300 µL supplemented with 1x NEB cut smart buffer (NEB) and 1 mM of UDP-glucose (Sigma-Aldrich) for 18 h at 37 °C. Then 3 µg of glucosylated or mock treated DNA was used for digestion with 100 units of MspI (NEB) at 37 °C for 18 h in a final volume of 50 µL, which was followed by treatment with 20 µg of proteinase K (PK, Carl Roth GmbH) for 30 min at 50 °C. Following proteolysis, PK was inactivated for 10 min at 98 °C. The MspI-resistant fraction was amplified using PCR with primers flanking the MspI site (Supplementary Table 4, primers). After PCR, the relative amounts of 5hmC were analyzed as described previously^66^. To detect 5mC at position 482 in L1 5’UTR before Tet1 transfection, the gDNA was treated with or without T4-BGT as described above. The gDNA was further treated with MspI and HpaII (50 units each, NEB) or mock for 18 h at 37 °C. The PCR reactions were performed as described above, and quantification of PCR products was done by analytic gel densitometry^67^ using Fiji: After electrophoresis, a digital image of the gel was taken and densitometric readings obtained. The total pixel density for each lane was determined by drawing a rectangle around the bands and measuring the area of the intensity peaks. Each measurement was normalized by the non-digested control.

### Bisulfite sequencing and TAB (Tet-assisted bisulfite) sequencing

Bisulfite DNA sequencing analyses were performed as previously described^113,114^. Briefly, genomic DNA from MCF cells was isolated as described before. Next, 0.5 micrograms of genomic DNA were bisulfite converted using an EpiTect Bisulfite kit (Qiagen, Hilden, Germany) following manufacturer’s instructions. To determine the DNA methylation and hydroxymethylation status of the LINE 1 protomor and Alu repeats, we performed PCR sequencing using primers in Supplementary Table 4. To this end, 300–500 ng of converted genomic DNA were used in a 50 ml PCR reaction as follows: 2 min at 95 °C, 35 cycles of 30 s at 94 °C followed by 30 s at 54 °C and 60 s at 72 °C, and a final extension of 10 min at 72 °C. Amplified products were visualized as single bands in agarose gels and cloned in pCR4-TOPO TA Vector optimized for sequencing (ThermoFisher Scientific) and at least 20 individual clones were sequenced for each sample. The unique sequence in each clone was analyzed using QUMA at http://quma.cdb.riken.jp/^115^. Next, the percent of methylated and hydroxymethylated CpG sites was calculated by comparison to a consensus sequence from untreated DNA.

Tet-assisted bisulfite (TAB) sequencing was performed as described in previous studies^44,116^. Briefly, GFP-Tet1-CD was purified using GBP beads from Sf9 insect cells infected with the recombinant baculovirus coding for mouse Tet1-CD with N-terminal GFP-tag (pc2838)^44^. Afterwards, control DNA samples from GluMS-PCR experiments, in which 5hmC was converted to 5ghmC by incubation with T4-BGT, were incubated in an oxidation reaction during 4 hwith recombinant Tet1-CD, at 37 °C in Tet oxidation buffer (10 M Fe(NH4)2(SO4)2.6H2O, 100 mM NaCl, 50 mM HEPES (pH 8), 1.2 mM adenosine triphosphate (ATP), 2.5 mM dithiothreitol (DTT), 1 mM a-ketoglutarate (aKG) and 2 mM L-ascorbic acid). Following Tet1-CD incubation, the reaction was stopped by the addition of 20 g of proteinase K at 50 °C for 2 h. Bisulfite treatment, PCR, cloning, and sequence analysis was performed as described above.

### Slot blotting

The catalytic activity of the purified proteins was verified by performing an oxidation reaction with genomic DNA from MCF7 and MCF10a cells. gDNA samples were denatured at 99 °C for 10 min and placed quickly on ice for 5 min. Denatured gDNA was mixed with ice cold 20× saline–sodium citrate (SSC) buffer at a final concentration of 4.8× SSC and blotted on a nitrocellulose membrane (Bio-Rad Laboratories, Hercules, CA, USA), which was pre-equilibrated in 20× SSC. After air-drying, the membrane was blocked with 3% milk in PBST (PBS containing 0.1% Tween) for 30 min at room temperature (RT), followed by incubation with either mouse rabbit 5mC (1:1000) or rabbit anti 5caC (1:1000, Active Motif, La Hulpe, Belgium) antibodies for 2 h at RT. The membrane was washed 3 times for 10 min with PBST, before it was incubated with secondary antibody anti-rabbit IgG Cy3 (The Jackson Laboratory, Bar Harbor, ME, USA) for 1 h at RT and imaged with the AI600 (GE Healthcare, Chicago, II, USA). The amount of DNA loaded for each sample was verified by staining the membrane with Methylene blue for 5 min (Sigma-Aldrich).

For protein samples, purified mouse and human TET1 was spotted directly onto the nitrocellulose membrane, which were incubated in blocking buffer (5% (w/v) non-fat dry milk in PBS for 1 h at room temperature. Anti-Tet1/Tet1s primary antibody was used undiluted and incubated overnight at 4 °C, followed by three washes in PBS/0.1% Tween-20. Subsequently, membranes were incubated for 1 h at room temperature with secondary antibody anti-rat IgG Cy3 (The Jackson Laboratory, Bar Harbor, ME, USA) and imaged as described above. The amount of protein loaded for each sample was determined with Pierce 660 nm protein assay (Thermo Scientific). Antibodies details are summarized in Supplementary Table 5.

### Homology modeling

A structure homology model of Tet1s was generated using the automated SWISS-MODEL homology modeling server pipeline^117^. To this end, an atomic-resolution model of Tet1s was constructed based on amino acids 579–1980 of Tet1 (NP_001240786.1) using the crystal structure of Tet2^60^ as template. The generated model was visualized with UCSF Chimera (https://www.cgl.ucsf.edu/chimera/). Software is indicated in Supplementary Table 7.

### Statistics and reproducibility

Data visualization and statistical analysis were performed using RStudio (versions V1.2.1335, V1.2.5033 and 1.1.447), https://rstudio.com/). In all figures showing boxplots, the box represents 50% of the data, starting in the first quartile (25%) and ending in the third (75%). The line inside represents the median. The whiskers represent the upper and lower quartile. Outliers are excluded and defined as 1.5 times the interquartile range. Barplots show the average value of the distribution and the whiskers represent the standard deviation with a 95% confidence interval. Bar and line plots show normalized averaged values, and error bars show the respective standard deviation. Line intensity profile plots represent the fluorescence intensities along the distance of the selected arrow segment. For the statistics, independent two-group comparison was made for all conditions with Wilcoxon-Mann-Whitney or One-Way ANOVA tests. Related to this, n.s., not significant, is given for *p*-values > or equal to 0.05; one star (*) is given for *p*-values < 0.05 and > or equal to 0.005; two stars (**) is given for values < 0.005 and > or equal to 0.0005; three stars (***) is given for values < 0.0005; between the top of two boxes subjected to comparison. All statistical values (number (#) of cells (N), mean, median, standard deviation (SD), standard error of the mean (SEM), 95% confidence interval (CI) and *p*-values are summarized in Supplementary Data 1. No statistical method was used to predetermine sample size. Investigators were not blinded during the experiments and when assessing the outcome. All cells analyzed showed the reported behavior of the representative images shown in the respective figures.

### Reporting summary

Further information on research design is available in the Nature Research Reporting Summary linked to this article.

## Supplementary information

Supplementary Information

Description of Additional Supplementary Files

Supplementary data 1

Supplementary data 2

Supplementary Video 1

Supplementary Video 2

Reporting Summary

## Data Availability

The data that support this study are available from the corresponding authors upon reasonable request. All data sets have been deposited and are available at 10.48328/tudatalib-594.3. Source data are provided with this paper.

## Source data

Source data

## Code availability

Code used in this study for assessment of DAPI intensity classes (Supplementary Fig. 5G) is available under https://bioimaginggroup.github.io/nucim/ and was published in ref. 61. All custom written R scripts and ImageJ macros are available (10.48328/tudatalib-594.3).

## References

[1] Baylin SB, Jones PA (2011). A decade of exploring the cancer epigenome—biological and translational implications. Nat. Rev. Cancer.

[2] Ludwig AK, Zhang P, Cardoso MC (2016). Modifiers and readers of DNA modifications and their impact on genome structure, expression, and stability in disease. Front. Genet..

[3] Goll MG, Bestor TH (2005). Eukaryotic cytosine methyltransferases. Annu. Rev. Biochem..

[4] Chuang LS (1997). Human DNA-(cytosine-5) methyltransferase-PCNA complex as a target for p21WAF1. Science.

[5] Hashimoto H, Horton JR, Zhang X, Cheng X (2009). UHRF1, a modular multi-domain protein, regulates replication-coupled crosstalk between DNA methylation and histone modifications. Epigenetics.

[6] Liu X (2013). UHRF1 targets DNMT1 for DNA methylation through cooperative binding of hemi-methylated DNA and methylated H3K9. Nat. Commun..

[7] Qin W (2015). DNA methylation requires a DNMT1 ubiquitin interacting motif (UIM) and histone ubiquitination. Cell Res..

[8] Tahiliani M (2009). Conversion of 5-methylcytosine to 5-hydroxymethylcytosine in mammalian DNA by MLL partner TET1. Science.

[9] Ito S (2011). Tet proteins can convert 5-methylcytosine to 5-formylcytosine and 5-carboxylcytosine. Science.

[10] Bauer C (2015). Phosphorylation of TET proteins is regulated via O-GlcNAcylation by the O-linked N-acetylglucosamine transferase (OGT). J. Biol. Chem..

[11] Santiago M, Antunes C, Guedes M, Sousa N, Marques CJ (2014). TET enzymes and DNA hydroxymethylation in neural development and function - how critical are they?. Genomics.

[12] Ko M (2013). Modulation of TET2 expression and 5-methylcytosine oxidation by the CXXC domain protein IDAX. Nature.

[13] Jin S-G (2016). Tet3 reads 5-carboxylcytosine through Its CXXC domain and is a potential guardian against neurodegeneration. Cell Rep..

[14] Frauer C (2011). Different binding properties and function of CXXC zinc finger domains in Dnmt1 and Tet1. PLoS ONE.

[15] Jin C (2014). TET1 is a maintenance DNA demethylase that prevents methylation spreading in differentiated cells. Nucleic Acids Res..

[16] Zhang W (2016). Isoform switch of TET1 regulates DNA demethylation and mouse development. Mol. Cell.

[17] Good CR (2017). A novel isoform of TET1 that lacks a CXXC domain is overexpressed in cancer. Nucleic Acids Res..

[18] Yosefzon Y (2017). An epigenetic switch repressing Tet1 in gonadotropes activates the reproductive axis. Proc. Natl Acad. Sci. USA.

[19] Yu C (2013). CRL4 complex regulates mammalian oocyte survival and reprogramming by activation of TET proteins. Science.

[20] Nakagawa T (2015). CRL4(VprBP) E3 ligase promotes monoubiquitylation and chromatin binding of TET dioxygenases. Mol. Cell.

[21] Vidal E (2017). A DNA methylation map of human cancer at single base-pair resolution. Oncogene.

[22] Li L (2016). Epigenetic inactivation of the CpG demethylase TET1 as a DNA methylation feedback loop in human cancers. Sci. Rep..

[23] Guo M, Peng Y, Gao A, Du C, Herman JG (2019). Epigenetic heterogeneity in cancer. Biomark. Res..

[24] Eleftheriou M (2015). 5-Carboxylcytosine levels are elevated in human breast cancers and gliomas. Clin. Epigenetics.

[25] Bachman M (2014). 5-Hydroxymethylcytosine is a predominantly stable DNA modification. Nat. Chem..

[26] Bachman M (2015). 5-Formylcytosine can be a stable DNA modification in mammals. Nat. Chem. Biol..

[27] Löb D (2016). 3D replicon distributions arise from stochastic initiation and domino-like DNA replication progression. Nat. Commun..

[28] Chagin VO (2016). 4D Visualization of replication foci in mammalian cells corresponding to individual replicons. Nat. Commun..

[29] Burns KH, Boeke JD (2012). Human transposon tectonics. Cell.

[30] International Human Genome Sequencing Consortium. (2001). Initial sequencing and analysis of the human genome. Nature.

[31] Solovei I, Thanisch K, Feodorova Y (2016). How to rule the nucleus: divide et impera. Curr. Opin. Cell Biol..

[32] Quentin Y (1994). A master sequence related to a free left Alu monomer (FLAM) at the origin of the B1 family in rodent genomes. Nucleic Acids Res..

[33] Natale F (2018). DNA replication and repair kinetics of Alu, LINE-1 and satellite III genomic repetitive elements. Epigenetics Chromatin.

[34] Korenberg JR, Rykowski MC (1988). Human genome organization: Alu, lines, and the molecular structure of metaphase chromosome bands. Cell.

[35] Martin SL, Bushman FD (2001). Nucleic acid chaperone activity of the ORF1 protein from the mouse LINE-1 retrotransposon. Mol. Cell. Biol..

[36] Cost GJ, Feng Q, Jacquier A, Boeke JD (2002). Human L1 element target-primed reverse transcription in vitro. EMBO J..

[37] Zhang P (2017). L1 retrotransposition is activated by Ten-eleven-translocation protein 1 and repressed by methyl-CpG binding proteins. Nucleus.

[38] Casas-Delucchi CS (2011). Histone acetylation controls the inactive X chromosome replication dynamics. Nat. Commun..

[39] Heinz KS (2018). Peripheral re-localization of constitutive heterochromatin advances its replication timing and impairs maintenance of silencing marks. Nucleic Acids Res..

[40] Zhong X (2017). Ten-eleven translocation-2 (Tet2) is involved in myogenic differentiation of skeletal myoblast cells in vitro. Sci. Rep..

[41] Brero A (2005). Methyl CpG-binding proteins induce large-scale chromatin reorganization during terminal differentiation. J. Cell Biol..

[42] Rausch C (2020). Developmental differences in genome replication program and origin activation. Nucleic Acids Res..

[43] Zhang P (2017). Methyl-CpG binding domain protein 1 regulates localization and activity of Tet1 in a CXXC3 domain-dependent manner. Nucleic Acids Res..

[44] Ludwig AK (2017). Binding of MBD proteins to DNA blocks Tet1 function thereby modulating transcriptional noise. Nucleic Acids Res..

[45] Grewal SIS, Jia S (2007). Heterochromatin revisited. Nat. Rev. Genet..

[46] Lewis JD (1992). Purification, sequence, and cellular localization of a novel chromosomal protein that binds to methylated DNA. Cell.

[47] Chagin VO (2019). Processive DNA synthesis is associated with localized decompaction of constitutive heterochromatin at the sites of DNA replication and repair. Nucleus.

[48] Sharif J (2007). The SRA protein Np95 mediates epigenetic inheritance by recruiting Dnmt1 to methylated DNA. Nature.

[49] Lande-Diner L (2007). Role of DNA methylation in stable gene repression. J. Biol. Chem..

[50] Casas-Delucchi CS, Becker A, Bolius JJ, Cardoso MC (2012). Targeted manipulation of heterochromatin rescues MeCP2 Rett mutants and re-establishes higher order chromatin organization. Nucleic Acids Res..

[51] Lindhout BI (2007). Live cell imaging of repetitive DNA sequences via GFP-tagged polydactyl zinc finger proteins. Nucleic Acids Res.

[52] Bostick M (2007). UHRF1 plays a role in maintaining DNA methylation in mammalian cells. Science.

[53] Rothbauer U (2008). A versatile nanotrap for biochemical and functional studies with fluorescent fusion proteins. Mol. Cell. Proteom..

[54] Qin W, Leonhardt H, Spada F (2011). Usp7 and Uhrf1 control ubiquitination and stability of the maintenance DNA methyltransferase Dnmt1. J. Cell. Biochem..

[55] Görisch SM (2008). Uncoupling the replication machinery: replication fork progression in the absence of processive DNA synthesis. Cell Cycle.

[56] Baranovskiy AG (2014). Structural basis for inhibition of DNA replication by aphidicolin. Nucleic Acids Res..

[57] Rausch C (2021). Cytosine base modifications regulate DNA duplex stability and metabolism. Nucleic Acids Res..

[58] Easwaran HP, Leonhardt H, Cardoso MC (2007). Distribution of DNA replication proteins in Drosophila cells. BMC Cell Biol..

[59] Cartron P-F (2013). Identification of TET1 partners that control its DNA-demethylating function. Genes Cancer.

[60] Hu L (2013). Crystal structure of TET2-DNA complex: insight into TET-mediated 5mC oxidation. Cell.

[61] Schmid VJ, Cremer M, Cremer T (2017). Quantitative analyses of the 3D nuclear landscape recorded with super-resolved fluorescence microscopy. Methods.

[62] Kim K (2013). VprBP has intrinsic kinase activity targeting histone H2A and represses gene transcription. Mol. Cell.

[63] McCall CM (2008). Human immunodeficiency virus type 1 Vpr-binding protein VprBP, a WD40 protein associated with the DDB1-CUL4 E3 ubiquitin ligase, is essential for DNA replication and embryonic development. Mol. Cell. Biol..

[64] Kisselev, A. F. Site-specific proteasome inhibitors. *Biomolecules* **12**, 54 (2021). 10.3390/biom12010054 PMC8773591 35053202

[65] Soucy TA (2009). An inhibitor of NEDD8-activating enzyme as a new approach to treat cancer. Nature.

[66] Davis, T. & Vaisvila, R. High sensitivity 5-hydroxymethylcytosine detection in Balb/C brain tissue. *J. Vis. Exp*. 10.3791/2661. (2011). 10.3791/2661 PMC3339836 21307836

[67] Tan HY, Ng TW (2008). Accurate step wedge calibration for densitometry of electrophoresis gels. Opt. Commun..

[68] Jin S-G, Kadam S, Pfeifer GP (2010). Examination of the specificity of DNA methylation profiling techniques towards 5-methylcytosine and 5-hydroxymethylcytosine. Nucleic Acids Res..

[69] Yu M (2012). Base-resolution analysis of 5-hydroxymethylcytosine in the mammalian genome. Cell.

[70] Booth MJ (2012). Quantitative sequencing of 5-methylcytosine and 5-hydroxymethylcytosine at single-base resolution. Science.

[71] Gaudet F (2003). Induction of tumors in mice by genomic hypomethylation. Science.

[72] Palumbo D, Affinito O, Monticelli A, Cocozza S (2018). DNA Methylation variability among individuals is related to CpGs cluster density and evolutionary signatures. BMC Genomics.

[73] Smith ZD, Meissner A (2013). DNA methylation: roles in mammalian development. Nat. Rev. Genet..

[74] Müller U, Bauer C, Siegl M, Rottach A, Leonhardt H (2014). TET-mediated oxidation of methylcytosine causes TDG or NEIL glycosylase dependent gene reactivation. Nucleic Acids Res.

[75] Moldovan G-L, Pfander B, Jentsch S (2007). PCNA, the maestro of the replication fork. Cell.

[76] Leonhardt H, Page AW, Weier HU, Bestor TH (1992). A targeting sequence directs DNA methyltransferase to sites of DNA replication in mammalian nuclei. Cell.

[77] Jia Y (2016). Negative regulation of DNMT3A de novo DNA methylation by frequently overexpressed UHRF family proteins as a mechanism for widespread DNA hypomethylation in cancer. Cell Discov..

[78] Weissmann S (2012). Landscape of TET2 mutations in acute myeloid leukemia. Leukemia.

[79] Lee S-G (2013). Genomic breakpoints and clinical features of MLL-TET1 rearrangement in acute leukemias. Haematologica.

[80] Beck CR, Garcia-Perez JL, Badge RM, Moran JV (2011). LINE-1 elements in structural variation and disease. Annu. Rev. Genomics Hum. Genet..

[81] Hancks DC, Kazazian HH (2016). Roles for retrotransposon insertions in human disease. Mob. DNA.

[82] Lee E (2012). Landscape of somatic retrotransposition in human cancers. Science.

[83] Scott EC (2016). A hot L1 retrotransposon evades somatic repression and initiates human colorectal cancer. Genome Res..

[84] Jachowicz JW (2017). LINE-1 activation after fertilization regulates global chromatin accessibility in the early mouse embryo. Nat. Genet..

[85] Tufarelli C, Cruickshanks HA, Meehan RR (2013). LINE-1 activation and epigenetic silencing of suppressor genes in cancer: Causally related events?. Mob. Genet. Elem..

[86] Ting DT (2011). Aberrant overexpression of satellite repeats in pancreatic and other epithelial cancers. Science.

[87] Scheller M (2021). Hotspot DNMT3A mutations in clonal hematopoiesis and acute myeloid leukemia sensitize cells to azacytidine via viral mimicry response. Nat. Cancer.

[88] Hsu C-H (2012). TET1 suppresses cancer invasion by activating the tissue inhibitors of metalloproteinases. Cell Rep..

[89] Song SJ (2013). MicroRNA-antagonism regulates breast cancer stemness and metastasis via TET-family-dependent chromatin remodeling. Cell.

[90] Yaffe D, Saxel O (1977). Serial passaging and differentiation of myogenic cells isolated from dystrophic mouse muscle. Nature.

[91] Sheng C (2019). PCNA-mediated degradation of p21 coordinates the DNA damage response and cell cycle regulation in individual cells. Cell Rep..

[92] Becker A (2016). Poly(ADP-ribosyl)ation of methyl CpG binding domain protein 2 regulates chromatin structure. J. Biol. Chem..

[93] Agarwal N (2011). MeCP2 Rett mutations affect large scale chromatin organization. Hum. Mol. Genet..

[94] Bostock CJ, Prescott DM, Kirkpatrick JB (1971). An evaluation of the double thymidine block for synchronizing mammalian cells at the G1-S border. Exp. Cell Res..

[95] Mulholland CB (2015). A modular open platform for systematic functional studies under physiological conditions. Nucleic Acids Res..

[96] Ho SN, Hunt HD, Horton RM, Pullen JK, Pease LR (1989). Site-directed mutagenesis by overlap extension using the polymerase chain reaction. Gene.

[97] Jeong J-Y (2012). One-step sequence- and ligation-independent cloning as a rapid and versatile cloning method for functional genomics studies. Appl. Environ. Microbiol..

[98] Rueden CT (2017). ImageJ2: ImageJ for the next generation of scientific image data. BMC Bioinform..

[99] RStudio-Team. *RStudio: Integrated Development for R. RStudio* (PBC, 2020).

[100] Rottach A (2010). The multi-domain protein Np95 connects DNA methylation and histone modification. Nucleic Acids Res..

[101] Koulouras G (2018). EasyFRAP-web: a web-based tool for the analysis of fluorescence recovery after photobleaching data. Nucleic Acids Res..

[102] Herce HD, Deng W, Helma J, Leonhardt H, Cardoso MC (2013). Visualization and targeted disruption of protein interactions in living cells. Nat. Commun..

[103] Weichmann F (2020). Validation strategies for antibodies targeting modified ribonucleotides. RNA.

[104] Becker A (2016). Poly(ADP-ribosyl)ation of methyl CpG binding protein 2 regulates chromatin structure. J. Biol. Chem..

[105] Rottach A, Kremmer E, Nowak D, Leonhardt H, Cardoso MC (2008). Generation and characterization of a rat monoclonal antibody specific for multiple red fluorescent proteins. Hybrid. (Larchmt.).

[106] Wilson IA (1984). The structure of an antigenic determinant in a protein. Cell.

[107] Miller SA, Dykes DD, Polesky HF (1988). A simple salting out procedure for extracting DNA from human nucleated cells. Nucleic Acids Res..

[108] Skene PJ (2010). Neuronal MeCP2 is expressed at near histone-octamer levels and globally alters the chromatin state. Mol. Cell.

[109] Schelter F (2021). 5-hydroxymethyl-, 5-formyl- and 5-carboxydeoxycytidines as oxidative lesions and epigenetic marks. Chem. Eur. J..

[110] Pfaffeneder T (2014). Tet oxidizes thymine to 5-hydroxymethyluracil in mouse embryonic stem cell DNA. Nat. Chem. Biol..

[111] Wagner M (2015). Age-dependent levels of 5-methyl-, 5-hydroxymethyl-, and 5-formylcytosine in human and mouse brain tissues. Angew. Chem. Int. Ed..

[112] Szwagierczak A, Bultmann S, Schmidt CS, Spada F, Leonhardt H (2010). Sensitive enzymatic quantification of 5-hydroxymethylcytosine in genomic DNA. Nucleic Acids Res..

[113] Wissing S (2012). Reprogramming somatic cells into iPS cells activates LINE-1 retroelement mobility. Hum. Mol. Genet..

[114] Coufal NG (2009). L1 retrotransposition in human neural progenitor cells. Nature.

[115] Kumaki Y, Oda M, Okano M (2008). QUMA: quantification tool for methylation analysis. Nucleic Acids Res..

[116] Yu M, Han D, Hon GC, He C (2018). Tet-assisted bisulfite sequencing (TAB-seq). Methods Mol. Biol..

[117] Bienert S (2017). The SWISS-MODEL Repository-new features and functionality. Nucleic Acids Res..

