# Author Correction: Isoform-specific and ubiquitination dependent recruitment of Tet1 to replicating heterochromatin modulates methylcytosine oxidation

**DOI:** 10.1038/s41467-023-36067-1

**Published:** 2023-01-23

**Authors:** María Arroyo, Florian D. Hastert, Andreas Zhadan, Florian Schelter, Susanne Zimbelmann, Cathia Rausch, Anne K. Ludwig, Thomas Carell, M. Cristina Cardoso

**Affiliations:** 1 Cell Biology and Epigenetics, Department of Biology, Technical University of Darmstadt, Schnittspahnstr. 10, 64287 Darmstadt, Germany; 2 Section AIDS and newly emerging pathogens, Paul Ehrlich Institute, Paul-Ehrlich-Str. 51-59, 63225 Langen, Germany; 3 Department of Chemistry, Ludwig Maximilians University, Butenandstr. 5-13, 81377 Munich, Germany; 4 Present Address: Luxembourg Centre for Systems Biomedicine, University of Luxembourg, 6, avenue du Swing, L-4367 Belvaux, Luxembourg; 5 Present Address: Department of Medicine, Hematology, Oncology and Rheumatology, University Hospital Heidelberg, Im Neuenheimer Feld 410, 69120 Heidelberg, Germany

**Keywords:** Cellular imaging, Nuclear organization, Ubiquitylation, DNA methylation

Correction to: *Nature Communications* 10.1038/s41467-022-32799-8, published online 02 September 2022

In the original article the y-axes for Figure 8b had been interchanged; the figure should have appeared as shown below.


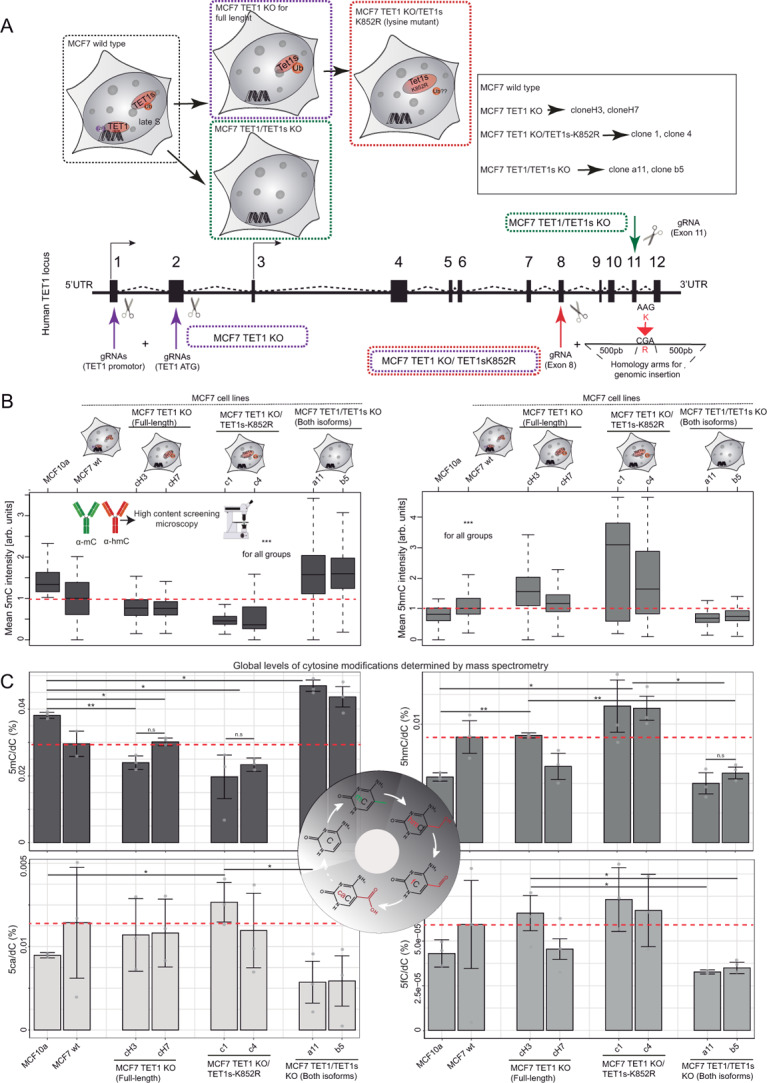


This has now been corrected in both the PDF and HTML versions of the Article.

