# Supplementary material for: Isoform-specific and ubiquitination dependent recruitment of Tet1 to replicating heterochromatin modulates methylcytosine oxidation
Source: Nat Commun. 2022 Sep 2;13:5173. doi: 10.1038/s41467-022-32799-8 (PMC9440122; doi:10.1038/s41467-022-32799-8)
Supplement: Supplementary file 1 — Supplementary Information [file 41467_2022_32799_MOESM1_ESM.pdf]

Supplementary information

Isoform-specific and ubiquitination dependent recruitment of Tet1 to replicating heterochromatin modulates methylcytosine oxidation

María Arroyo[1], Florian D. Hastert[1,2,*], Andreas Zhadan[1], Florian Schelter[3], Susanne Zimbelmann[1], Cathia Rausch[1], Anne K. Ludwig[1], Thomas Carell[3], M. Cristina Cardoso[1,*]

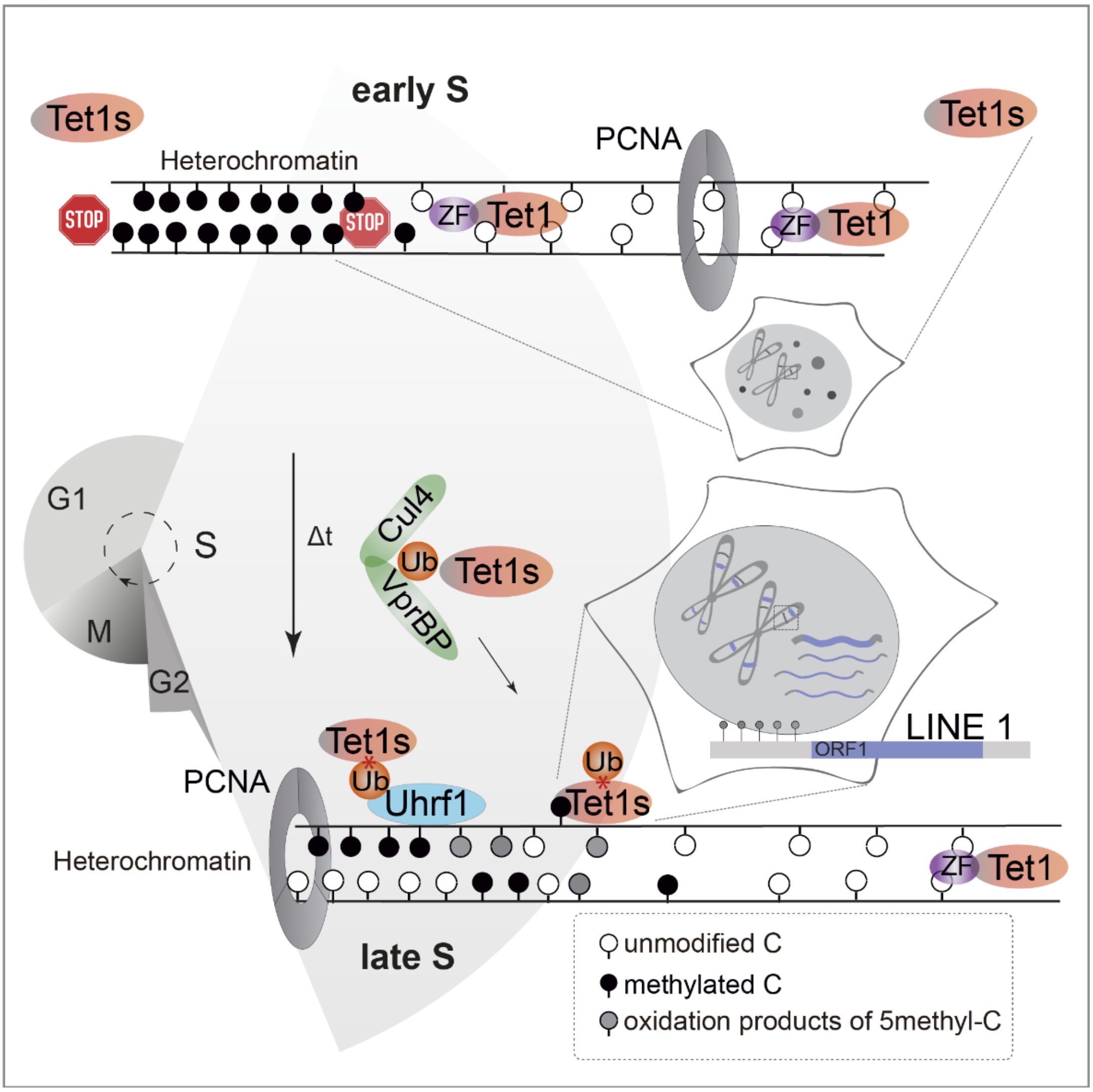

Supplementary graphical abstract

Supplementary figures and legends:

Supplementary Fig. 1 (Related to Figure 1). MCF7 breast cancer cells overexpress TET1 proteins and show aberrant cytosine modification levels.

Supplementary Fig. 2 (Related to Figure 2). Comparison of endogenous and ectopic Tet1/Tet1s protein levels.

Supplementary Fig. 3 (Related to Figure 4). Effect of Tet1 zinc finger domain on Tet1 binding kinetics.

Supplementary Fig. 4 (Related to Figure 5). Tet1s association with replicating heterochromatin is dependent on Uhrf1 and DNA replication but not 5mC nor PCNA.

Supplementary Fig. 5 (Related to Figure 6). The CRD of Tet1s is required for its catalytic activity and S-phase heterochromatin localization that coincides with chromatin decompaction.

Supplementary Fig. 6 (Related to Figure 7). Effect of VprBP and Cul4 in Tet1s cell cycle localization, ubiquitination and interactions.

Supplementary Fig. 7 (Related to Figure 8). Characterization of MCF7 CRISPR/Cas9 edited cell lines.

Supplementary Fig. 8 (Related to Figure 9). Bisulfite and TAB sequencing of L1 promoter versus Alu repeat regions.

Supplementary Fig. 9. Schematic summary of the different Tet constructs used in this study.

Supplementary tables:

Supplementary Table 1: Cell lines
Supplementary Table 2: Plasmids
Supplementary Table 3: Oligonucleotides used for cloning
Supplementary Table 4: Primers
Supplementary Table 5: Antibodies
Supplementary Table 6: Imaging Systems
Supplementary Table 7: Software and macros

Supplementary References

Supplementary figures and legends

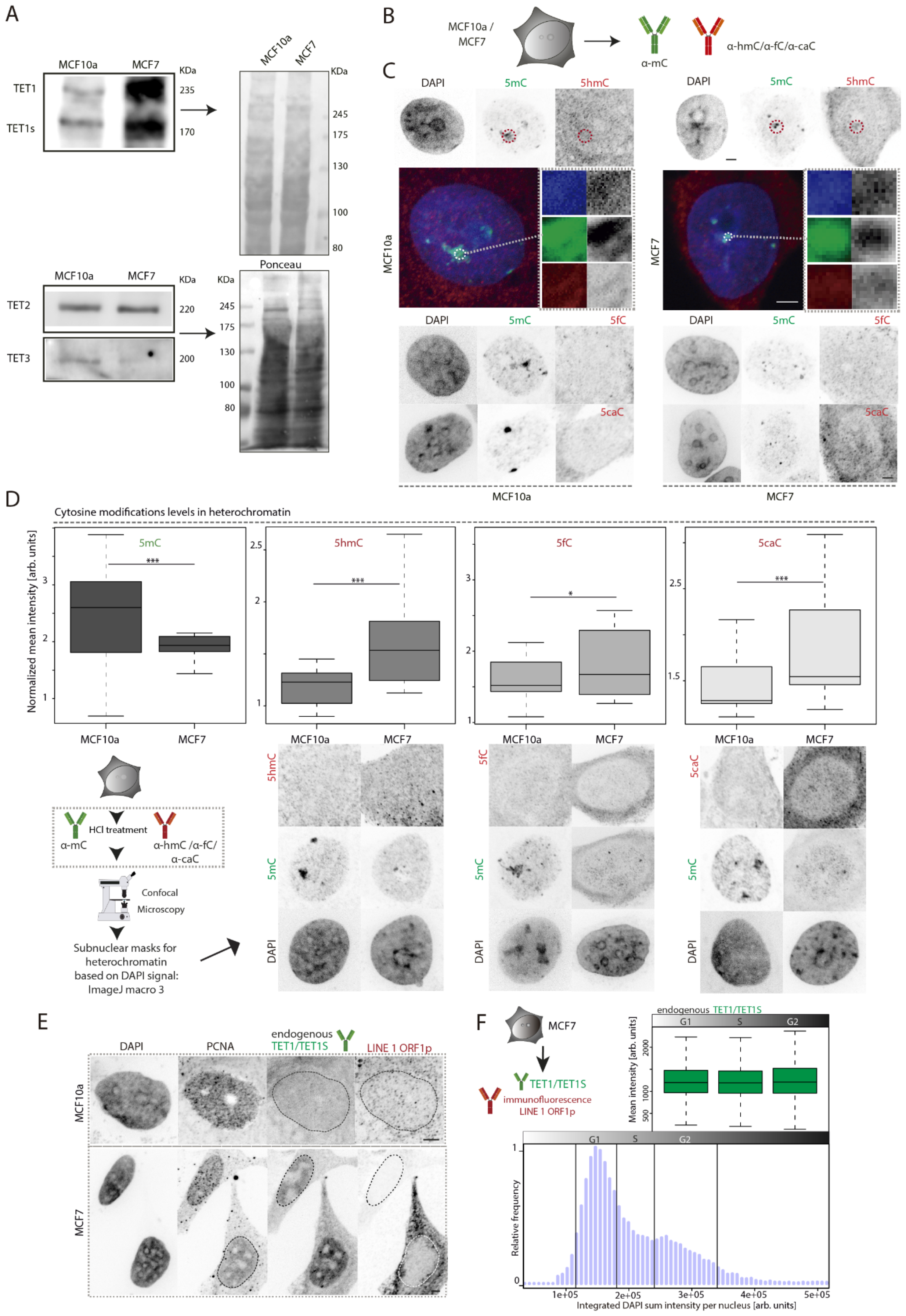

Supplementary Fig. 1 (Related to Figure 1). **MCF7 breast cancer cells overexpress TET1 proteins and show aberrant cytosine modification levels.**

(A) Analysis by western blotting of TET1/TET1s, TET2 and TET3 levels in MCF10a and MCF7 cells. The cut-outs show both TET1 isoforms and Ponceau staining shows the total amount of protein. Two independent experiments were performed.

(B) Scheme of the experimental procedure for C: MCF10a and MCF7 cells were immunostained against cytosine modifications and DNA counterstained with DAPI.

(C) Representative confocal mid-Z-sections of DAPI stained nuclei of MCF10a and MCF7 cells showing levels of 5mC and 5hmC/5fC/5caC in heterochromatin-rich regions. Confocal Z-stacks were acquired and analyzed with Fiji. Selected ROIs in heterochromatic regions were magnified in all channels and are shown.

(D) Boxplots showing the normalized mean intensity of cytosine modifications at heterochromatin regions after immunostaining with HCl treatment. The experimental scheme and representative confocal images are shown. n = 25 (MCF10a) and n = 31 (MCF7) cells.

(E) Representative confocal images of MCF10a and MCF7 cells immunostained against LINE 1 ORF1p and endogenous TET1/TET1s.

(F) Boxplot showing endogenous levels of TET1/TET1s analyzed by immunofluorescence and their distribution during different cell cycle stages. Cells were classified in G1, S or G2 using the level of DAPI sum intensity.

For all boxplots, the box represents 50% of the data, starting in the first quartile (25%) and ending in the third (75%). The line inside represents the median. The whiskers represent the upper and lower quartile. Statistical significance was tested with a paired two-samples Wilcoxon test using R-studio (n.s., not significant, is given for p-values ≥ 0.05; one star (*) for p-values < 0.05 and ≥ 0.005; two stars (**) is given for values < 0.005 and ≥ 0.0005; three stars (***) is given for values < 0.0005). Source data are provided as a Source Data file. Scale bars = 5 μm.

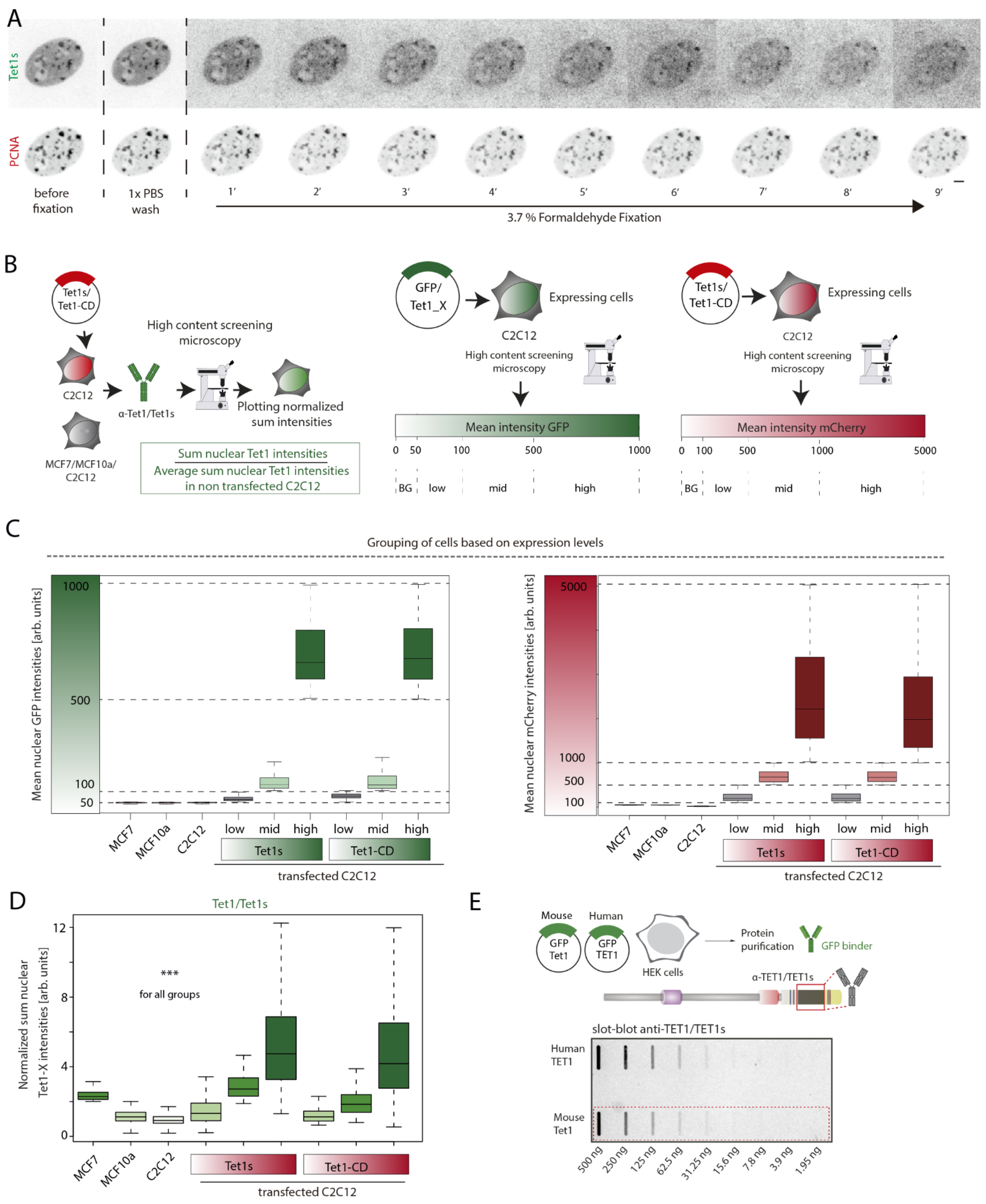

Supplementary Fig. 2 (Related to Figure 2). Comparison of endogenous and ectopic Tet1/Tet1s protein levels.
(**A**) Representative images of time-lapse confocal microscopy analysis of Tet1s before and after (1-9 minutes) formaldehyde fixation of C2C12 cell expressing mRFP-PCNA and EGFP-Tet1s. Cells in late S-phase showing Tet1s heterochromatin accumulation are shown.
(B) Workflow of the experimental procedure for the analysis of Tet1/Tet1s immunostaining, and scheme illustrating the classification of C2C12 transfected cells based on the mean fluorescence intensity of the EGFP or mcherry fusion tags. Fluorescence intensities are in arbitrary units (AU) and imaging conditions were kept constant throughout the experiments.
(C) Boxplot showing the different grouping of the cells according to their respective mean EGFP fluorescence (low: 50-100 AU, mid: 100-500 AU, high: 500-1000 AU) or mean mcherry fluorescence (low: 100-500 AU, mid: 500-1000

AU, high: 1000-5000 AU). Cells with fluorescence intensity below these values were considered as background non-transfected cells. Left panel n = 48488 (MCF7), 16037 (MCF10a), 28766 (C2C12), 16786 (Tet1s), 61066 (Tet1-CD) cells. Right panel n = 16321 (MCF7), 25855 (MCF10a), 28766 (C2C12), 1287 (Tet1s), 4961 (Tet1-CD) cells.
(D) Immunostaining against Tet1/Tet1s in MCF10a, MCF7 and C2C12 cells. To compare endogenous Tet1/Tet1s with levels after ectopic overexpression, we transfected C2C12 with mcherry-Tet1s and mcherry-Tet1-CD, immunostained against Tet1/Tet1s and counterstained the DNA with DAPI. Sum nuclear levels of Tet1/Tet1s and mean nuclear levels of mcherry were measured with a wide-field high content microscopy system, and transfected cells were grouped according to their respective mean mcherry fluorescence. Boxplot shows sum nuclear Tet1/Tet1s levels in the different cell lines, normalized to the average sum nuclear intensity of non-transfected C2C12, as this cell line exhibited the lowest Tet1/Tet1s levels.
(E) Comparison of the TET1/TET1s antibody binding to the human and mouse protein by slot blot. TET1 protein functional domains are indicated and the region used as an epitope for antibody generation is marked. GFP-tagged human and mouse TET1 were purified from HEK293 cells using GFP binder, and serial dilutions were blotted as indicated on a nitrocellulose membrane and detected with a Tet1-specific monoclonal antibody,
For all boxplots, the box represents 50% of the data, starting in the first quartile (25%) and ending in the third (75%). The line inside represents the median. The whiskers represent the upper and lower quartile. N-numbers and p-values are shown in Supplementary Data 1. Source data are provided as a Source Data file.

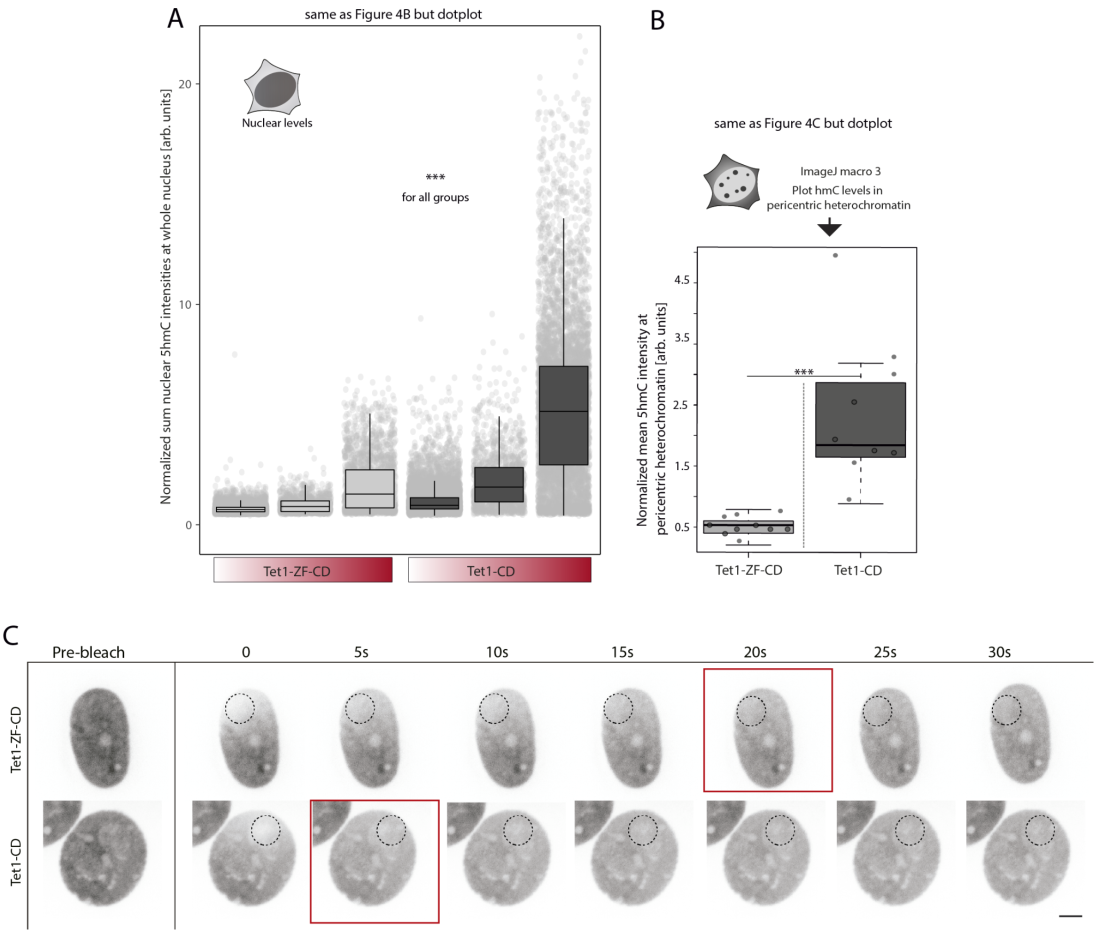

Supplementary Fig. 3 (Related to Figure 4). Effect of Tet1 zinc finger domain on Tet1 binding kinetics.
(A) and (B) Bloxplots combined with dotplots for data shown in Figures 4B and C.
(C) Representative confocal microscopy images of FRAP experiments. Time-lapse microscopy of C2C12 cells expressing mCherry-Tet1-CD or mCherry-Tet1-ZF-CD. Shown is one pre-bleach image and time points every 5 seconds after bleaching. Bleaching regions are indicated by dotted circles. Red frames depict the approximated timing of fluorescence recovery.
For all boxplots, the box represents 50% of the data, starting in the first quartile (25%) and ending in the third (75%). The line inside represents the median. The whiskers represent the upper and lower quartile. Statistical significance was tested with a paired two-samples Wilcoxon test using R-studio (n.s., not significant, is given for p-values ≥ 0.05; one star (*) for p-values < 0.05 and ≥ 0.005; two stars (**) is given for values < 0.005 and ≥ 0.0005; three stars (***) is given for values < 0.0005). N-numbers and p-values are shown in Supplementary Data 1. Source data are provided as a Source Data file. Back scale bars = 5 µm.

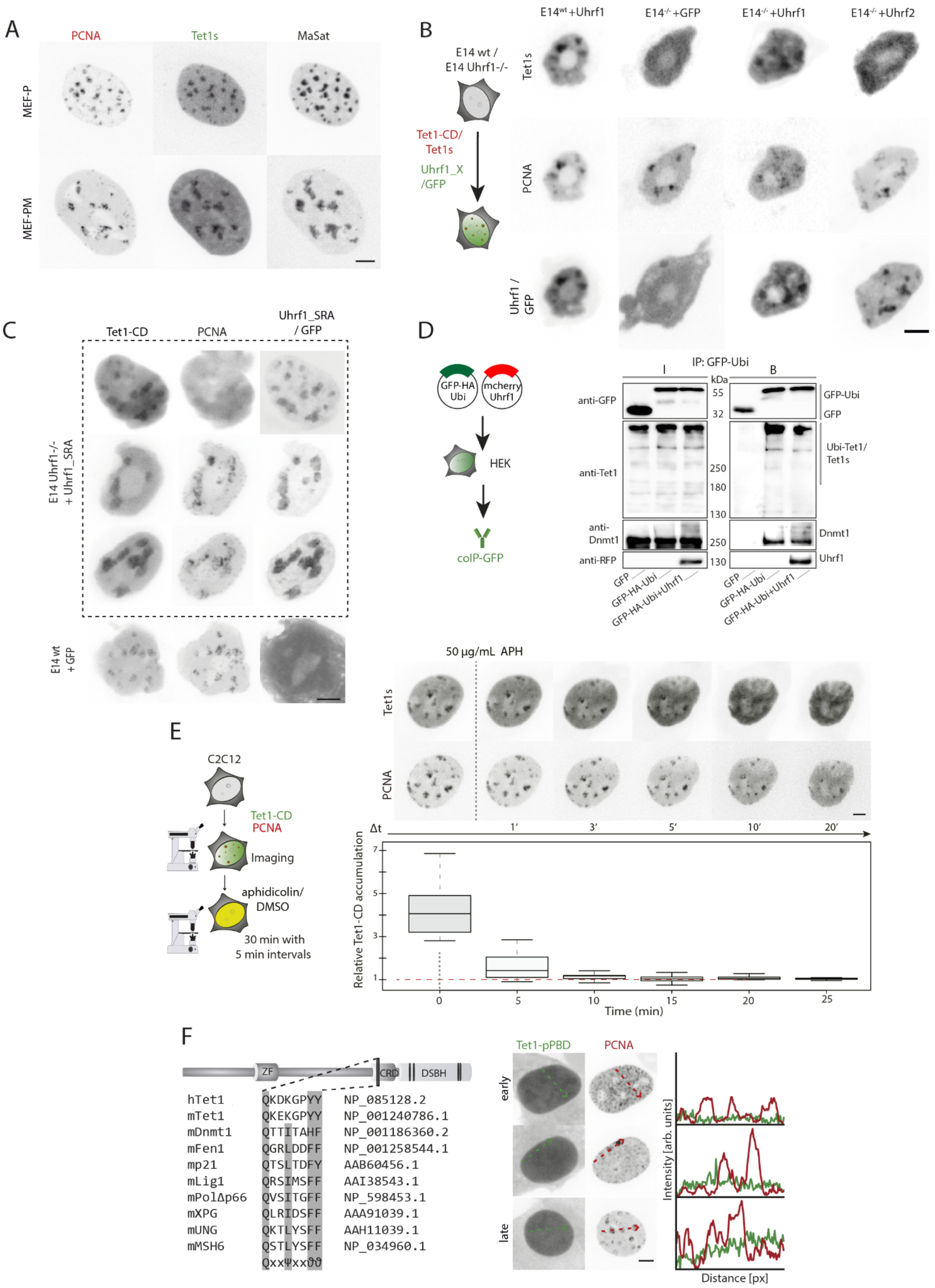
A
PCNA
Tet1s
MaSat
MEF-P
MEF-PM
B
E14 wt /
E14 Uhrf1-/-
Tet1-CD/
Tet1s
Uhrf1_X
/GFP
E14wt +Uhrf1
E14-/- +GFP
E14-/- +Uhrf1
E14-/- +Uhrf2
Tet1s
PCNA
Uhrf1 /
GFP
C
Tet1-CD
PCNA
Uhrf1_SRA
/ GFP
E14 Uhrf1-/-
+ Uhrf1_SRA
E14 wt
+ GFP
D
GFP-HA
Ubi
mcherry
Uhrf1
HEK
coIP-GFP
IP: GFP-Ubi
I
B
kDa
55
32
250
180
130
250
130
anti-GFP
anti-Tet1
anti-
Dnmt1
anti-RFP
GFP-Ubi
GFP
Ubi-Tet1/
Tet1s
Dnmt1
Uhrf1
GFP
GFP-HA-Ubi
GFP-HA-Ubi+Uhrf1
E
C2C12
Tet1-CD
PCNA
Imaging
aphidicolin/
DMSO
30 min with
5 min intervals
50 µg/mL APH
Tet1s
PCNA
Δt
1′
3′
5′
10′
20′
Relative Tet1-CD accumulation
Time (min)
0
5
10
15
20
25
1
3
5
7
F
ZF
CRD
DSBH
hTet1 QKDKGPYY NP_085128.2
mTet1 QKEKGPYY NP_001240786.1
mDnmt1 QTTITAHF NP_001186360.2
mFen1 QGRLDDFF NP_001258544.1
mp21 QTSLTDFY AAB60456.1
mLig1 QRSIMSFF AAI38543.1
mPolΔp66 QVSITGFF NP_598453.1
mXPG QLRIDSFF AAA91039.1
mUNG QKTLYSFF AAH11039.1
mMSH6 QSTLYSFF NP_034960.1
QxxΨxxϑϑ
Tet1-pPBD
PCNA
early
late
Intensity [arb. units]
Distance [px]

Supplementary Fig. 4 (Related to Figure 5). Tet1s association with replicating heterochromatin is dependent on Uhrf1 and DNA replication but not 5mC nor PCNA.

(A) Representative confocal mid-Z-plane sections of mouse embryonic fibroblasts deficient for p53 (MEF-P) or p53 and Dnmt1 (MEF-PM) transiently expressing mcherry-Tet1s, miRFP-PCNA and EGFP-MaSat.

(B) Representative confocal mid-Z-plane sections of E14 and E14 Uhrf1-/- transfected with fluorescently tagged PCNA, Tet1s and either Uhrf1, Uhrf2 or EGFP.

(C) E14 and E14 Uhrf1-/- transfected with fluorescently tagged PCNA, Tet1-CD and either EGFP or Uhrf1_SRA domain. Representative images for 3 independent experiments are shown.

(D) HEK293-EBNA cells were transfected with EGFP or EGFP-HA-tagged ubiquitin, alone or co-transfected with mcherry-Uhrf1. Cell extracts were analyzed by immunoprecipitation using an immobilized antibody against EGFP followed by western blotting with antibodies against GFP, Tet1/Tet1s and Dnmt1. I depicts input and B depicts bound fraction. Two independent experiments were performed.

(E) C2C12 cells were transfected with mRFP-PCNA and EGFP-Tet1-CD. 8 hours after transfection, aphidicolin was added to the medium. Time lapse microscopy was performed before and after aphidicolin addition. Representative confocal mid-Z-plane sections are shown, and the boxplot depicts the quantification of Tet1-CD accumulation at late replicating heterochromatin in the different time points before and after aphidicolin addition.

(F) Schematic representation of Tet1 with the localization of the putative PCNA-binding domain within the cysteine-rich domain (CRD). The amino acid sequence of human and mouse Tet1 was aligned to known PBDs from different proteins associated with DNA replication and repair and their respective accession numbers are given. Exemplary confocal mid-Z-sections of C2C12 cells in different S-phase substages co-transfected with the EGFP-tagged pPBD of Tet1 and mRFP-tagged PCNA. Fluorescence intensity profiles of the lines are shown next to the respective images. Distance is indicated in pixels (px).

For all boxplots, the box represents 50% of the data, starting in the first quartile (25%) and ending in the third (75%). The line inside represents the median. The whiskers represent the upper and lower quartile. Statistical significance was tested with a paired two-samples Wilcoxon test using R-studio (n.s., not significant, is given for p-values ≥ 0.05; one star (*) for p-values < 0.05 and ≥ 0.005; two stars (**) is given for values < 0.005 and ≥ 0.0005; three stars (***) is given for values < 0.0005). N-numbers and p-values are shown in Supplementary Data 1. Source data are provided as a Source Data file. Scale bars = 5 µm.

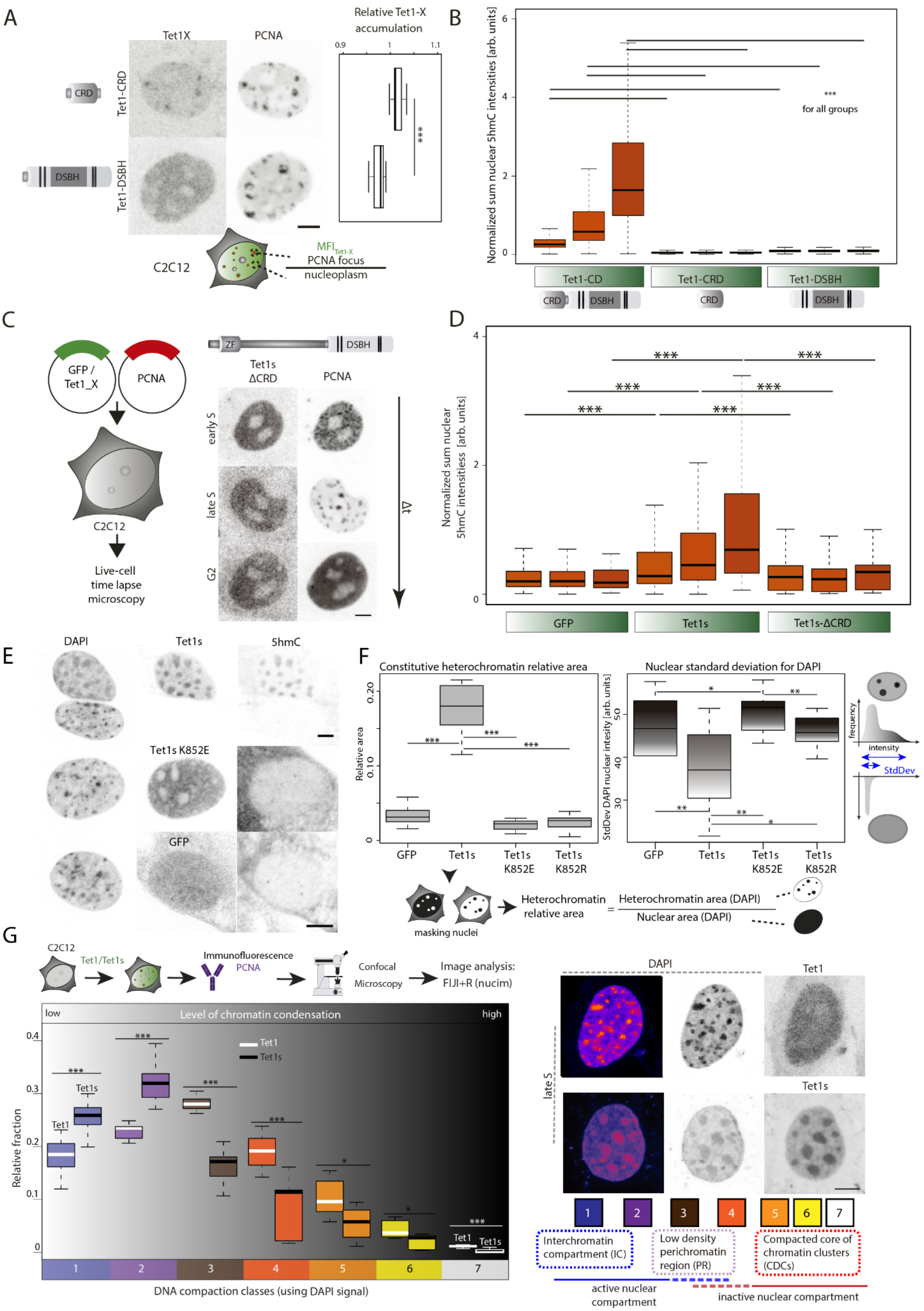

A
Tet1X
PCNA
Relative Tet1-X accumulation
Tet1-CRD
Tet1-DSBH
CRD
DSBH
C2C12
MFI
PCNA focus
nucleoplasm
B
Normalized sum nuclear 5hmC intensities [arb. units]
*** for all groups
Tet1-CD
Tet1-CRD
Tet1-DSBH
C
GFP / Tet1_X
PCNA
C2C12
Live-cell time lapse microscopy
ZF
DSBH
Tet1s ΔCRD
early S
late S
G2
Δt
D
Normalized sum nuclear 5hmC intensitiess [arb. units]
GFP
Tet1s
Tet1s-ΔCRD
E
DAPI
Tet1s
5hmC
Tet1s K852E
GFP
F
Constitutive heterochromatin relative area
Relative area
Nuclear standard deviation for DAPI
StdDev DAPI nuclear intesity [arb. units]
GFP
Tet1s
Tet1s K852E
Tet1s K852R
frequency
intensity
StdDev
masking nuclei
Heterochromatin relative area = Heterochromatin area (DAPI) / Nuclear area (DAPI)
G
C2C12
Tet1/Tet1s
Immunofluorescence
PCNA
Confocal Microscopy
Image analysis: FIJI+R (nucim)
low
Level of chromatin condensation
high
Tet1
Tet1s
Relative fraction
DNA compaction classes (using DAPI signal)
DAPI
Tet1
Tet1s
late S
Interchromatin compartment (IC)
Low density perichromatin region (PR)
Compacted core of chromatin clusters (CDCs)
active nuclear compartment
inactive nuclear compartment

Supplementary Fig. 5 (Related to Figure 6). The CRD of Tet1s is required for its catalytic activity and S-phase heterochromatin localization that coincides with chromatin decompaction.
(A) Representative confocal mid-Z-sections of C2C12 cells expressing fluorescently tagged PCNA and Tet1-CRD or Tet1-DSBH. Schematic of the accumulation quantification of Tet1-CRD or Tet1-DSBH in PCNA marked replicating heterochromatin. The corresponding boxplot summarizes the quantification results.
(B) Respective boxplots of C2C12 cells transfected with EGFP tagged Tet1-CD, Tet1-CRD or Tet1-DSBH, and 24 hours later stained against 5hmC. Sum nuclear 5hmC were normalized to the 5hmC levels in the lowest expressing cells and the sum nuclear DNA (DAPI) intensity and grouped according to their mean nuclear EGFP fluorescence intensity.
(**C**) Representative confocal mid-Z-section images of a C2C12 cell that was co-transfected with EGFP-Tet1sΔCRD and mRFP-PCNA and subjected to live-cell time-lapse microscopy.
(D) Respective boxplots of cells transfected with EGFP, EGFP-Tet1s or EGFP-Tet1sΔCRD and 24 hours later stained against 5hmC. Cells were grouped and sum nuclear 5hmC were normalized as described on B.
(E) Representative confocal mid-Z-plane sections of C2C12 cells transfected with EGFP-tagged Tet1s or Tet1s-K852E and subjected to immunostaining against 5hmC. DAPI channel shows heterochromatin spots, with Tet1s but not Tet1s K852E accumulation and the corresponding increase in 5hmC.
(F) Boxplots showing relative heterochromatin area and values for the standard deviation of the DAPI signal for the same cells.
(G) Analysis of chromatin structure in C2C12 transfected with Tet1 or Tet1s and imaged using confocal microscopy during late S phase. Boxplot shows chromatin compaction classification, together with representative images and a summary of the different compaction classes.
For all boxplots, the box represents 50% of the data, starting in the first quartile (25%) and ending in the third (75%). The line inside represents the median. The whiskers represent the upper and lower quartile. Statistical significance was tested with a paired two-samples Wilcoxon test using R-studio (n.s., not significant, is given for *p*-values ≥ 0.05; one star (*) for *p*-values < 0.05 and ≥ 0.005; two stars (**) is given for values < 0.005 and ≥ 0.0005; three stars (***) is given for values < 0.0005). N-numbers and p-values are shown in Supplementary Data 1. Source data are provided as a Source Data file. Scale bar = 5 μm.

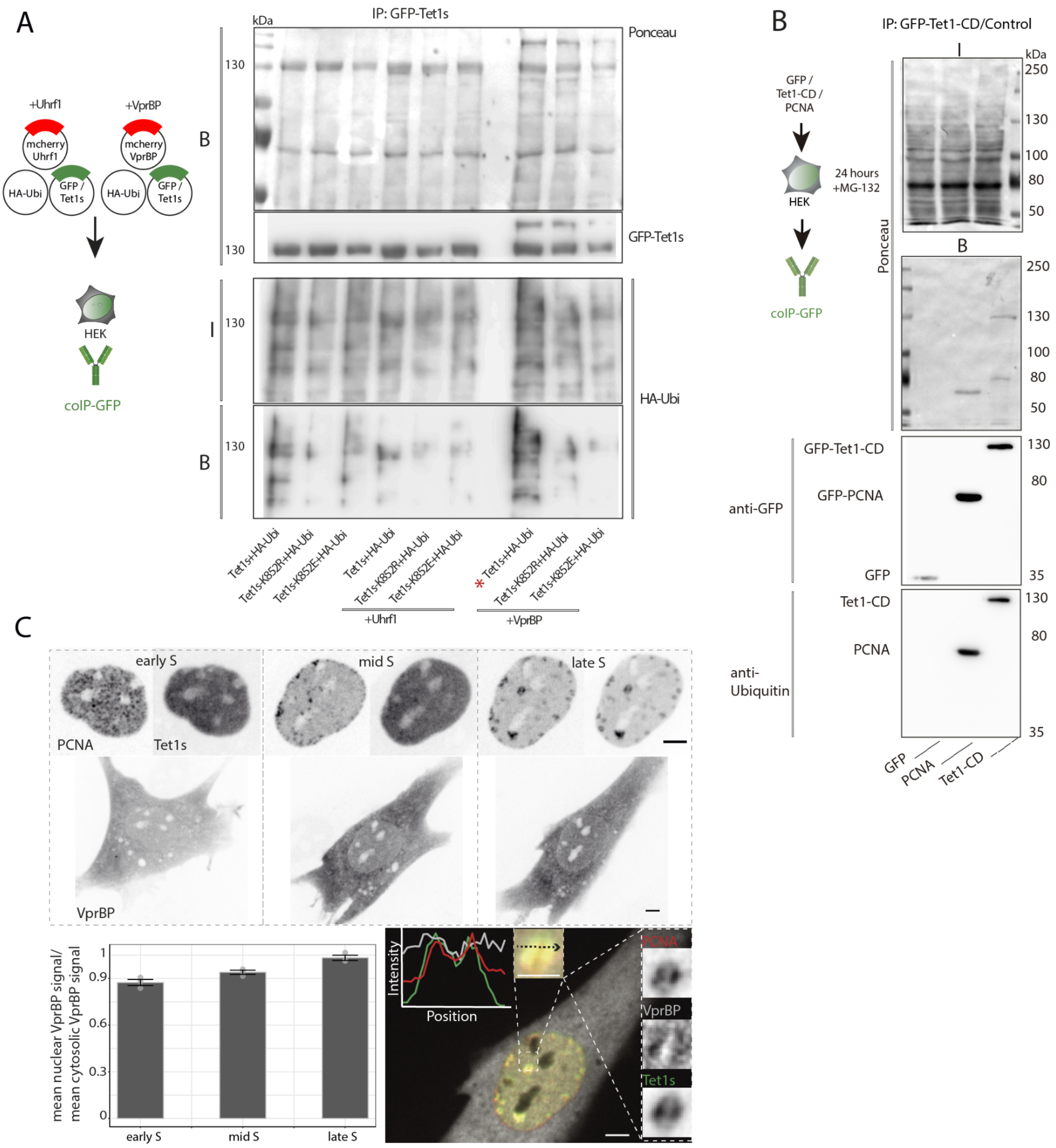

Supplementary Fig. 6 (Related to Figure 7). Effect of VprBP and Cul4 in Tet1s cell cycle localization, ubiquitination and interactions.

(A) EGFP-tagged Tet1s and HA-Ubiquitin, with and without Uhrf1 or VprBP, were ectopically overexpressed in HEK293-EBNA cells. Cell extracts were analyzed by immunoprecipitation using an immobilized GFP-binding nanobody followed by western blotting with antibodies against GFP and HA. The cut-outs show the bound GFP fractions and the input and bound HA-Ubi fractions. One experiment was performed.

(B) EGFP-tagged Tet1-CD and PCNA were ectopically overexpressed in HEK293-EBNA cells. Cell extracts were analyzed by immunoprecipitation using an immobilized GFP-binding nanobody followed by western blotting with antibodies against GFP and ubiquitin. The cut-outs show the bound GFP fractions and the input and bound ubiquitin fractions. Two independent experiments were performed.

(C) Representative mid-Z-plane images of C2C12 cells that were transfected with EGFP-Tet1s, miRFP-PCNA and mRFP-VprBP and subjected to live-cell time-lapse microscopy. Snapshots of the respective channels during early, mid and late S-phase are shown (top). Bar graphs represent the ratio of the mean nuclear to mean cytoplasmic fluorescence intensity of mRFP-VprBP (n=3). The error bars represent the standard deviation with a 95% confidence interval. Pseudocoloured, detailed examination of the late S-phase cell is shown. A heterochromatin spot marked by PCNA and Tet1s was examined in detail by line profile analysis. Scale bars = 5 µm, except scale bar in blow-up for line-profile = 2 µm.

Source data are provided as a Source Data file.

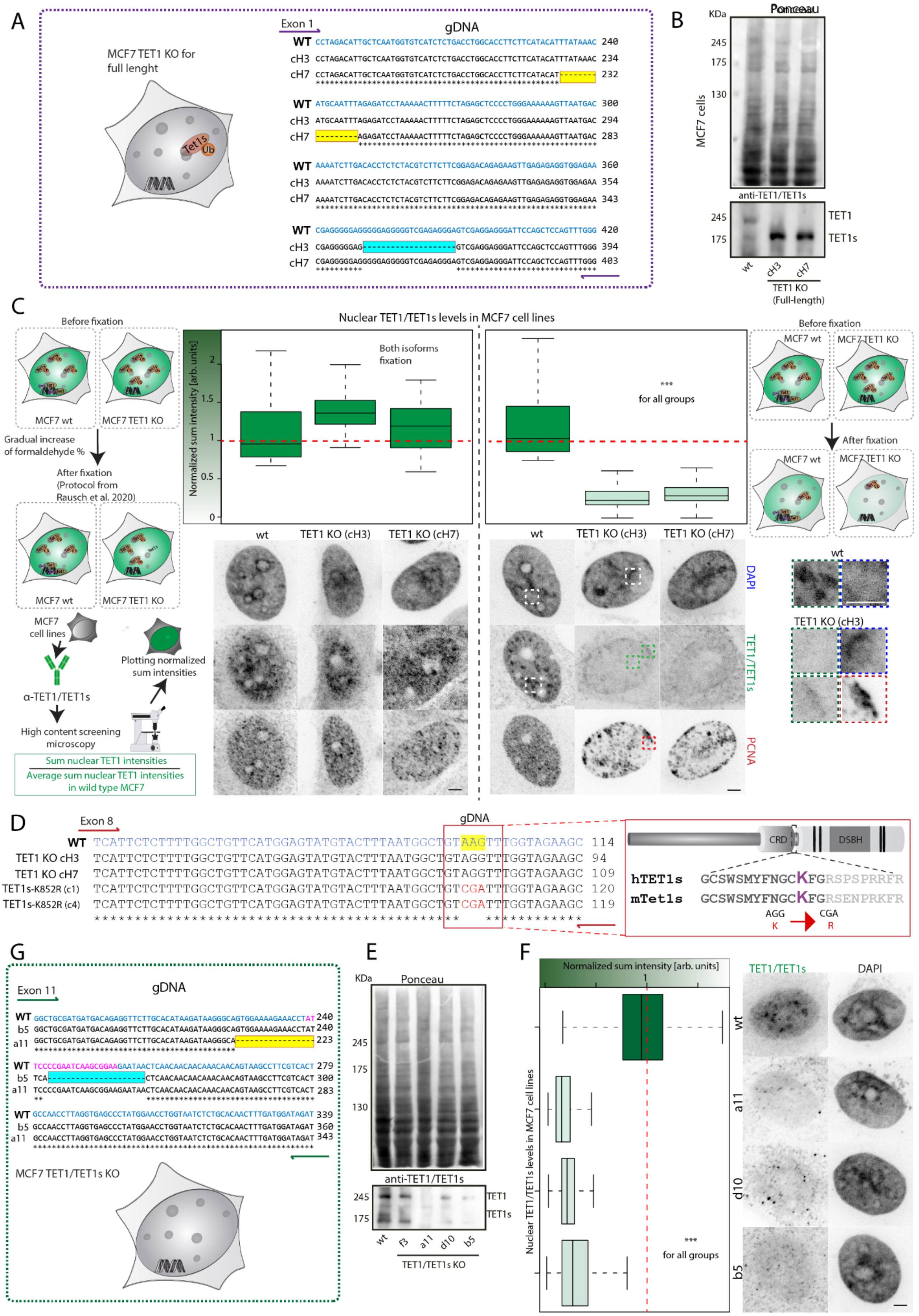
A
MCF7 TET1 KO for full lenght
Tet1s
Ub
Exon 1
gDNA
WT
cH3
cH7
B
KDa
Ponceau
245
175
130
MCF7 cells
anti-TET1/TET1s
TET1
TET1s
wt
cH3
cH7
TET1 KO (Full-length)
C
Before fixation
MCF7 wt
MCF7 TET1 KO
Gradual increase of formaldehyde %
After fixation (Protocol from Rausch et al. 2020)
MCF7 cell lines
α-TET1/TET1s
High content screening microscopy
Plotting normalized sum intensities
Sum nuclear TET1 intensities
Average sum nuclear TET1 intensities in wild type MCF7
Nuclear TET1/TET1s levels in MCF7 cell lines
Normalized sum intensity [arb. units]
Both isoforms fixation
***
for all groups
wt
TET1 KO (cH3)
TET1 KO (cH7)
DAPI
TET1/TET1s
PCNA
TET1 KO (cH3)
D
Exon 8
gDNA
WT
TET1 KO cH3
TET1 KO cH7
TET1s-K852R (c1)
TET1s-K852R (c4)
CRD
DSBH
hTET1s
mTet1s
AGG
CGA
K
R
G
Exon 11
gDNA
WT
b5
a11
MCF7 TET1/TET1s KO
E
KDa
Ponceau
245
175
130
anti-TET1/TET1s
TET1
TET1s
wt
f3
a11
d10
b5
TET1/TET1s KO
F
Normalized sum intensity [arb. units]
Nuclear TET1/TET1s levels in MCF7 cell lines
***
for all groups
TET1/TET1s
DAPI
wt
a11
d10
b5

Supplementary Fig. 7 (Related to Figure 8). Characterization of MCF7 CRISPR/Cas9 edited cell lines.
(A) Genomic PCR and DNA sequencing of MCF7 TET1 KO cell clones. Sequences from single clones are aligned to the wild-type sequence (WT) in blue. Genomic deletions are highlighted in yellow and cyan.
(B) Analysis by western blotting of TET1/TET1s levels for MCF7 TET1 KO clones compared with MCF7 wild type. The cut-outs show both TET1 isoforms and Ponceau staining shows the total amount of protein.
(C) Immunofluorescence analysis of endogenous TET1/TET1s levels for the cell clones in (A) and (B). Schemes show the different fixation protocols used and boxplots show the sum intensity of TET1 proteins measured by high content microscopy and normalized to the average level of MCF7 wild type cells. Below, are representative confocal images of the respective immunofluorescence stainings. Magnifications show in detail the distribution of DAPI, PCNA and TET1/TET1s in clone cH3.
(D) Genomic PCR and DNA sequencing of MCF7 TET1 KO/TET1s-K852R cell clones confirm the mutation K to R. Single clones cells are aligned to the wild-type sequence (WT) and clones for the TET1 KO (cH3 and cH7). AAG encoding for lysine (K) is highlighted in yellow.
(E) Genomic PCR and DNA sequencing of MCF7 TET1/TET1s (both isoforms) KO cell clones. Single clone cells are aligned to the wild-type sequence (WT) in blue. Deletions are highlighted in yellow and cyan. The target sequence of the gRNA is highlighted in pink.
(F) Analysis of TET1/TET1s levels by western blotting for MCF7 TET1/TET1s KO clones (both isoforms) compared with MCF7 wild type. The cut-outs show the levels of both TET1 isoforms and the Ponceau staining shows the total amount of protein.
(G) Immunofluorescence analysis of endogenous TET1/TET1s levels for the cell clones in (E). Boxplots show the sum intensity of TET1 proteins measured by high content microscopy normalized to the average level in MCF7 wild type. Representative confocal images of the immunofluorescence stainings for the different clones. Clones a11 and b5 were selected for further experiments.
For all boxplots, the box represents 50% of the data, starting in the first quartile (25%) and ending in the third (75%). The line inside represents the median. The whiskers represent the upper and lower quartile.Statistical significance was tested with a paired two-samples Wilcoxon test using R-studio (n.s., not significant, is given for p-values ≥ 0.05; one star (*) for p-values < 0.05 and ≥ 0.005; two stars (**) is given for values < 0.005 and ≥ 0.0005; three stars (***) is given for values < 0.0005). Source data are provided as a Source Data file. Scale bars = 5 μm, except scale bar in magnified regions = 2 μm.

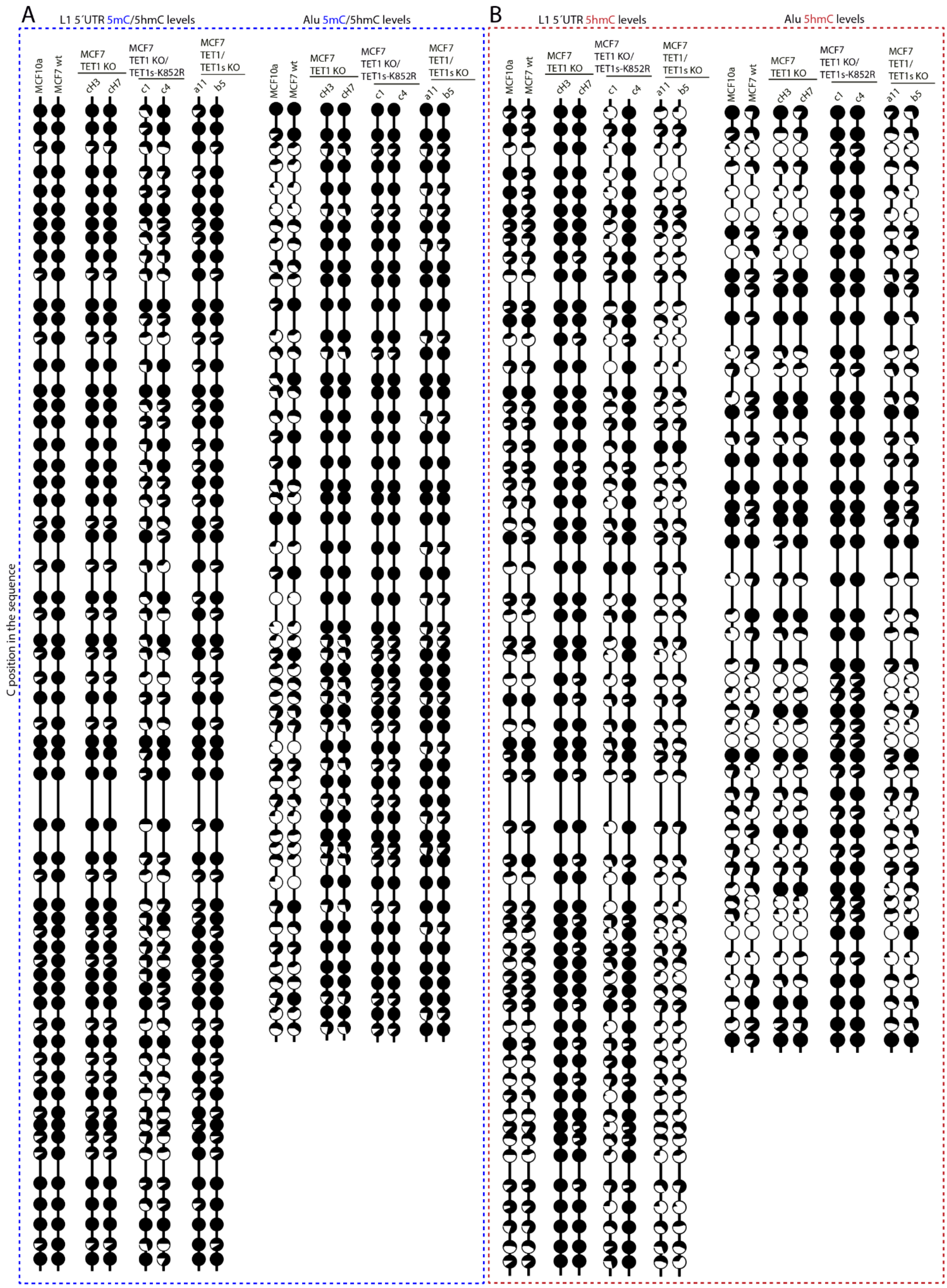

Supplementary Fig. 8 (Related to Figure 9). Bisulfite and TAB sequencing of L1 promoter versus Alu repeat regions. Comparison of MCF10a, MCF7 wild type and TET1 mutant cell lines. The methylation status percentage of sequences is presented: Each line depicts a different cell line. Circles indicate pie charts for the percent methylation status at the respective position, calculated from multiple clones. Black indicates methylation/hydroxymethylation

results from bisulfite sequencing experiments **(A)** or hydroxymethylation from TAB sequencing experiments **(B)**, and white indicates no cytosine modification in the respective position.

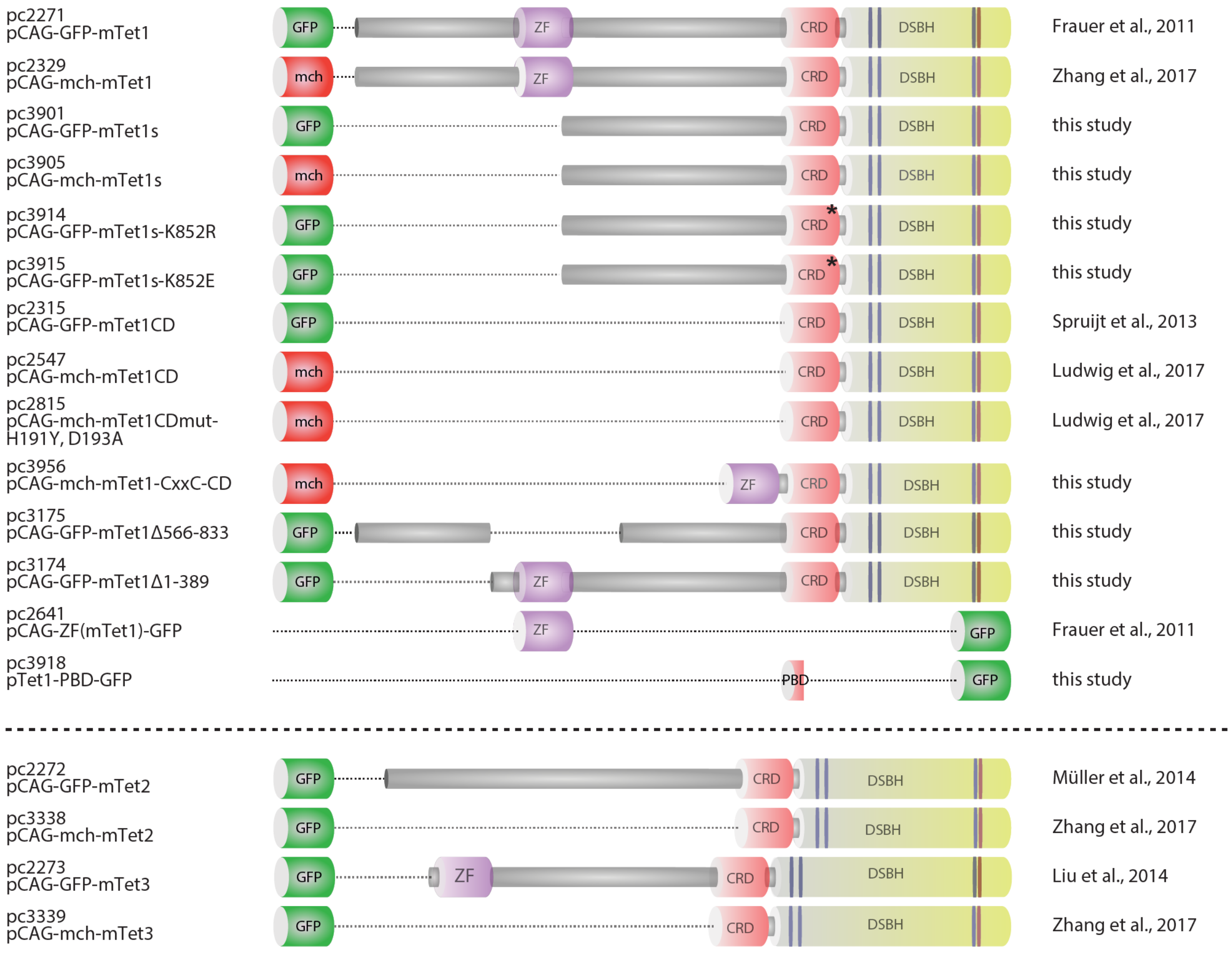

Supplementary Fig. 9. Schematic summary of the different Tet constructs used in this study. Main domains are indicated as in Figure 1A.

Supplementary tables

Supplementary Table 1: Cell lines

Name	Species	Type	Genotype	Sex	RRID*	Reference
C2C12	*Mus musculus*	myoblast	wildtype	female	CVCL_0188	[1]
HEK293-EBNA	*Homo sapiens*	embryonic kidney	wildtype	female	CVCL_6974	Invitrogen; Paisley, UK
MCF7	*Homo sapiens*	breast adenocarcinoma	wildtype	female	CVCL_0031	[2]
MCF7 TET1 KO cH3	*Homo sapiens*	breast adenocarcinoma	TET1 KO	female		This study
MCF7 TET1 KO cH7	*Homo sapiens*	breast adenocarcinoma	TET1 KO	female		This study
MCF7 TET1 KO/K852R c1	*Homo sapiens*	breast adenocarcinoma	TET1 KO/TET1s-K852R	female		This study
MCF7 TET1 KO/K852R c4	*Homo sapiens*	breast adenocarcinoma	TET1 KO/TET1s-K852R	female		This study
MCF7 TET1/TET1s KO a11	*Homo sapiens*	breast adenocarcinoma	TET1/TET1s KO	female		This study
MCF7 TET1/TET1s KO b5	*Homo sapiens*	breast adenocarcinoma	TET1/TET1s KO	female		This study
MCF10a	*Homo sapiens*	mammary gland	wildtype	female	CVCL_0598	[3]
MEF P	*Mus musculus*	embryonic fibroblast	$p53^{-/-}$	-	Children of CVCL_4240	[4]
MEF PM	*Mus musculus*	embryonic fibroblast	$p53^{-/-}$, $Dnmt1^{-/-}$	-	Children of CVCL_4240	[4]
E14-Uhrf1^{wt}	*Mus musculus*	embryonic stem cell	wildtype	male	CVCL_9108	[5]
E14-Uhrf1$^{-/-}$	*Mus musculus*	embryonic stem cell	$Uhrf1^{-/-}$	male	Children of CVCL_9108	[5]

* Research Resource Identifiers (https://www.rrids.org/)

Supplementary Table 2: Plasmids

Name	pc number*	Fluorophore	Gene species	Promoter	Reference
pEGFP-N1	0713	GFP	*Aequorea victoria*	CMV	Clontech
pmiRFP670-N1	3379	miRFP670	Synthetic	CMV	6,7
pmcherry	2387	mcherry	*Discosoma sp.*	CMV	8
pGFP-hPCNA	0653	GFP	*Homo sapiens*	CMV	9
pRFP-hPCNA	1054	RFP	*Homo sapiens*	CMV	7
pmiRFP-hPCNA	3385	miRFP	*Homo sapiens*	CMV	10
pEGFP-hDNMT1	1099	GFP	*Homo sapiens*	CMV	11,12
pMaSat-GFP	1803	GFP	-	CMV	13
pMaSat-miRFP670	3944	miRFP	-	CMV	This study
pGBP-MaSat	2469	-	-	CMV	14
pHA-Ubiquitin	1634	-	*S. cerevisiae*	CMV	15
pGFP-mUhrf1	1709	GFP	*Mus musculus*	CAG	16
pmcherry-mUhrf1	1756	mcherry	*Mus musculus*	CAG	15
pGFP-mUhrf1-ΔSRA	1933	GFP	*Mus musculus*	CAG	17
pGFP-mUhrf1-ΔPHD	1934	GFP	*Mus musculus*	CAG	16
pGFP-mUhrf1-ΔTTD	1935	GFP	*Mus musculus*	CAG	16
pGFP-(mUhrf1)-TTD	1936	GFP	*Mus musculus*	CAG	16
pGFP-(mUhrf1)-PHD	1937	GFP	*Mus musculus*	CAG	16
pGFP-(mUhrf1)-SRA	1938	GFP	*Mus musculus*	CAG	16
pmUhrf1-ΔUbl-GFP	2164	GFP	*Mus musculus*	CAG	18
pmUhrf1-ΔRing-GFP	2987	GFP	*Mus musculus*	CAG	18
p(mUhrf1)-Ubl-GFP	3061	GFP	*Mus musculus*	CAG	19
p(mUhrf1)-RING-GFP	3063	GFP	*Mus musculus*	CAG	18
pGFP-mUhrf2	1976	GFP	*Mus musculus*	CAG	17,19
pmch-mTet1-CD	2547	mcherry	*Mus musculus*	CAG	20
pmch-mTet1-CDmut	2815	mcherry	*Mus musculus*	CAG	20
pmcherry-mTet2-CD	3338	mcherry	*Mus musculus*	CAG	21,22
pmcherry-mTet3-CD	3339	mcherry	*Mus musculus*	CAG	20
pGFP-mTet1	2271	GFP	*Mus musculus*	CAG	23
pGFP-mTet2	2272	GFP	*Mus musculus*	CAG	24
pGFP-mTet3	2273	GFP	*Mus musculus*	CAG	25
pGFP-mTet1-Cys	2332	GFP	*Mus musculus*	CAG	This study
pGFP-mTet1-DSBH	2335	GFP	*Mus musculus*	CAG	This study
pGFP-mVprBP	2953	GFP	*Mus musculus*	CAG	This study
pGFP-mTDG	2422	GFP	*Mus musculus*	CAG	26
pmcherry-VprBP	2954	mcherry	*Mus musculus*	CAG	This study
piLenti-siRNA-VprBP-GFP	3922	GFP	-	CMV	abmgood, Richmond, C, Canada Cat.No.: 501630940495
pGFP-mTet1s	3901	GFP	*Mus musculus*	CAG	This study
pGFP-mTet1-delCys	3903	GFP	*Mus musculus*	CAG	This study
pGFP-mTet1s-delCys	3904	GFP	*Mus musculus*	CAG	This study
pmcherry-mTet1s	3905	mcherry	*Mus musculus*	CAG	This study
pGFP-mTet1s_K852R	3914	GFP	*Mus musculus*	CAG	This study
pGFP-mTet1s_K852E	3915	GFP	*Mus musculus*	CAG	This study
pGFP-mTet1-CD_K171R	3916	GFP	*Mus musculus*	CAG	This study
pGFP-mTet1-CD_K171E	3917	GFP	*Mus musculus*	CAG	This study
pmTet1-PBD_GFP	3918	GFP	*Mus musculus*	CMV	This study
pmch-mTet1-ZF-CD	3956	mcherry	*Mus musculus*	CAG	This study
pSpCas9(BB)-2A-Puro (PX459)	3926	-	-	CMV	27
pFBDMeGmTet1CD	2838	GFP	*Baculoviridae*	Polyhedrin	20

*pc number: plasmid collection number

Supplementary Table 3: Oligonucleotides used for cloning

ID	Oligonucleotide sequence (5' → 3')	Restriction Enzyme*
Tet1s_fwd	ATAAGCAGCGATCGCATGGACTGCAGTAGAC	AsiSI
Tet1_rev	ATAAGCAGCGGCCGCTTAGACCCAACGATTG	NotI
Tet1-ZF_fwd	ATAAGCAGCGATCGCTCTACGCCGC	AsiSI
Tet1-ZF_rev	ATAAGCAGCGATCGCAAGAGCCTCCT	AsiSI
Tet1-CRD_fwd	AAAAGCGATCGCATGGAAGCTGCACCCTGTGACTG	AsiSI
Tet1-CRD_rev	GAATGCGGCCGCTTACCCAAACTTACAGCC	NotI
Tet1-DSBH_fwd	AAGCGATCGCCCAAGGAAATTTAGGCTAC	AsiSI
Tet1-DSBH_rev	AAAAGCGGCCGCTTAGAGGGAACGATTG	NotI
Tet1-PBD_fwd	AATTCCATGAAACGGCGGCAAAAAGAAAAAGGCCCATATTATCACAAACGCCGGCTGC	EcoRI
Tet1-PBD_rev	CCGGGCAGCCGGCGTTTGTGATAATATGGGCCTTTTTCTTTTTGCCGCCGTTTCATGG	XmaI
Tet1Fl-ΔCys-outer_fwd	CTCGAGGCGATCGCATGTCTCGGTCC	AsiSI
Tet1s-ΔCys-outer_fwd	ATAAGCAGCGATCGCATGGACTGCAGTAGAC	AsiSI
Tet1-ΔCys-inner_fwd	TAGCTGGATTGAAGGAACAGAGGAGTGAAAACCCCAGAAA	-
Tet1-ΔCys-inner_rev	TTTCTGGGGTTTTCACTCCTCTGTTCCTTCAATCCAGCTA	-
Tet1-ΔCys-outer_rev	CGTTAACTGCGGCCGCTTAGACCCAAC	NotI
Tet1-Δ1-389-outer_fwd	AAAAGCGATCGCATGGCTATTACTATGCTAAACCAA	AsiSI
Tet1-Δ1-389-inner_rev	TGCAGCCTTCCTGTTCCTTCAATCCAGCTATCAGGTT	-
Tet1-Δ1-389-inner_fwd	ATAGCTGGATTGAAGGAACAGGAAGCTGCACCCTGT	-
Tet1-Δ1-389-outer_rev	GAATTCGTTAACTGCGGCCGC	NotI
Tet1-Δ566-833-outer_fwd	AAAAGCGATCGCATGGCTATTACTATGCTAAACCAA	AsiSI
Tet1-Δ566-833-inner_fwd	AGGTCGACTCCCAGCCACCATTGGCGGCGTAGAATT	-
Tet1-Δ566-833-inner_rev	ACGCCGCCAATGGTGGCTGGGAGTCGACCTCACCCT	-
Tet1-Δ566-833-outer_rev	GAATTCGTTAACTGCGGCCGC	NotI
Tet1s-K852X/-outer_fwd	GATCTCGAGGCGATCGCATGGAC	AsiSI
Tet1s-K852X/-outer_rev	CGTTAACTGCGGCCGCTTAGAC	NotI
Tet1s-K852E-inner_fwd	CAACGGCTGT*GAG*TTTGGGAGGAG	-
Tet1s-K852E-inner_rev	CTCCTCCCAAA*CTC*ACAGCCGTTG	-
Tet1s-K852R-inner_fwd	CAACGGCTGT*CGG*TTTGGGAGGAG	-
Tet1s-K852R-inner_rev	CTCCTCCCAAA*CCG*ACAGCCGTTG	-
miRFP670_fwd	ATCCACCGGTCGCCACCATGGTA	AgeI
miRFP670_rev	AAATGTACAGGCTCTCAAGCGCGGTGAT	BsrGI
VprBP_fwd	AAAAGCGATCGCATGACTACAGTAGTGGTAC	AsiSI
VprBP-inner_fwd	GTCTCTCTTCCCCGGACCCCTCG	-
VprBP-inner_rev	CGAGGGGTCCGGGGAAGAGAGAC	-
VprBP_rev	AAAAGCGGCCGCATCACTCATTCAGAGATAAG	NotI

*Restriction Enzyme Site (underlined and bold in oligonucleotide sequence), Point mutation sites are bold and italic

Supplementary Table 4: Primers and homologus recombination template

ID	Oligonucleotide sequence (5' → 3')	Purpose	Species	Reference
**TET1 promotor-1sense**	**CACCGTCTCTCGCTCAACTGTGCA**	**gRNA for CRISPR/Cas9 MCF7 TET1-/-**	***Homo sapiens***	**This study**
**TET1 promotor-1antisense**	**AAACTGCACAGTTGAGCGAGAGAC**	**gRNA for CRISPR/Cas9 MCF7 TET1-/-**	***Homo sapiens***	**This study**
**TET1 promotor-2sense**	**CACCGGGGAGACACTGCTGCTCCG**	**gRNA for CRISPR/Cas9 MCF7 TET1-/-**	***Homo sapiens***	**This study**
**TET1 promotor-2antisense**	**AAACCGGAGCAGCAGTGTCTCCCC**	**gRNA for CRISPR/Cas9 MCF7 TET1-/-**	***Homo sapiens***	**This study**
**TET1-ATG-1sense**	**CACCGCCTTCCAGATTAGTCAGGA**	**gRNA for CRISPR/Cas9 MCF7 TET1-/-**	***Homo sapiens***	**This study**
**TET1-ATG-1antisense**	**AAACTCCTGACTAATCTGGAAGGC**	**gRNA for CRISPR/Cas9 MCF7 TET1-/-**	***Homo sapiens***	**This study**
**TET1-ATG-2sense**	**CACCGGCGCAGGAAACAGAGTCAT**	**gRNA for CRISPR/Cas9 MCF7 TET1-/-**	***Homo sapiens***	**This study**
**TET1-ATG-2antisense**	**AAACATGACTCTGTTTCCTGCGCC**	**gRNA for CRISPR/Cas9 MCF7 TET1-/-**	***Homo sapiens***	**This study**
**TET1-K852R-sense**	**CACCGATCAATTCTAAATCTTCTG**	**gRNA for CRISPR/Cas9 MCF7 TET1-/-, TET1s-K852R**	***Homo sapiens***	**This study**
**TET1-K852R-antisense**	**AAACCAGAAGATTTAGAATTGATC**	**gRNA for CRISPR/Cas9 MCF7 TET1-/-, TET1s-K852R**	***Homo sapiens***	**This study**
**TET1-exon11-sense**	**CACCGTTCCGCTTGATTCGGGGAAT**	**gRNA for CRISPR/Cas9 MCF7 TET1/TET1s-/-**	***Homo sapiens***	[28]
**TET1-exon11-antisense**	**AAACATTCCCCGAATCAAGCGGAAC**	**gRNA for CRISPR/Cas9 MCF7 TET1/TET1s-/-**	***Homo sapiens***	[28]
**TET1-K852R HR* template**	**See row below**	**Homologous recombination template for CRISPR/Cas9 MCF7 TET1s-K852R**	***Homo sapiens***	**This study**
gctaGACCCCATCTCAAAAAAAAAAAAAAAAAAAAAAAAACACCAAAAAAAAAAAAAAGAAAAACCCAGGTTCTTTATTTCCCTATCTCAGACATCTCCAAATCAAACAATACTGTGAGTATTTTAGATCTGTATAGTTTATTCCCACTTATTTGGATTTGCAATCATGACCAAATATTTCTGCTAAAAATTTGGTTATAATAAAGTATAGTTAACCCTGGTCCCCTAGTCAGATTTCTTGAAAAAACAAAAGTTAGCTTTTTATTGTTAACATTAAACTATTTTAAATAAGTAATATAATAGATAAGTGTTATAACCGGTTCTTTAGGCATCCATCCTTCTAAAGCTATAGAAACCTGAAATGTTCTCTCTGAGGTATTGTAATGCTTCATCAATTCACTCTCTTGAATTACAATCTTACAGTCGTACCTGTACATGTCAAGGAATTGATCCAGAGACTTGTGGAGCTTCATTCTCTTTTGGCTGTTCATGGAGTATGTACTTTAATGGCTGTCGATTTGGTAGAAGCCCAAGCCCCAGAAGATTTAGAATTGATCCAAGCTCTCCCTTACATGTAAGTGTCCTTCTTTATTCAAATAATTTATTTTTGAATTACACATTGAATATGTAAGTGCATTCCTTTAACATTTTTTGAAACACTATAAAAACTAAATATTGAAAAGTAGTAATCAAATAGTAAAATTATATTTCTATTATATAAGTAAATAAATTAATTAAGACTCTAAATAAATTAGGTAAAGACTTTAGCTATCTCCCCAAATTCCAATTGTCCCTGGCTTCCTCCTCCTGCTCTACTACCCCACCCCAGCAAAATCATCATTAAAAGAACCTAGAATTTTATACTCAGCCAAAATAGGAAAAACCCATTTGCCAAAATTTCAAAGACTCAGAAAGTTTACTACCCACAGACCTACTATGAAAGAATTACTAAAGGACGTAATCCAGAAAGAAGATATATGAAACCGGAACGAATCAGTCCGGGACACGAGGCCATGGTGAGCAAAg				
**exon1_fw**	**CTTAGCTCTTCCTGCCCTTTC**	**PCR for screening, sequencing (TET1-/-)**	***Homo sapiens***	**This study**
**exon1_rev**	**TCGGGCAAACTTTCCAACT**	**PCR for screening, sequencing (TET1-/-)**	***Homo sapiens***	**This study**
**exon8_fw**	**CTGAAATGTTCTCTCTGAGGTATTG**	**PCR for screening, sequencing (K852R)**	***Homo sapiens***	**This study**
**exon8_rev**	**CATGTAAGGGAGAGCTTGGA**	**PCR for screening, sequencing (K852R)**	***Homo sapiens***	**This study**
**exon11_fw**	**gctctttaggttctgcctagc**	**PCR for screening, sequencing (TET1/TET1s -/-)**	***Homo sapiens***	[28]
**exon11_rev**	**ctccaaatatacccaagtgcag**	**PCR for screening (TET1/TET1s -/-)**	***Homo sapiens***	[28]
**Gapdh_fw**	**CCAACATACAGGTTTCTCCAG**	**ChIP + qPCR**	***Mus musculus***	[29]
**Gapdh_rev**	**CTGGAAAGCTGTGGCGTGATGG**	**ChIP + qPCR**	***Mus musculus***	[29]
**MajSat _fw**	**GGCGAGAAAACTGAAAATCACG**	**ChIP + qPCR**	***Mus musculus***	[29]
**MajSat_rev**	**AGGTCCTTCAGTGTGCATTTC**	**ChIP + qPCR**	***Mus musculus***	[29]
**ALu fw**	**GTCAGGAGATCGAGACCATCCC**	**GluMs-PCR/Bisulfite and TAB sequencing**	***Homo sapiens***	[30]
**ALu rev**	**TCCTGCCTCAGCCTCCCAAG**	**GluMs-PCR/Bisulfite and TAB sequencing**	***Homo sapiens***	[30]
**L1 5´UTR fw (309)**	**ATCCCACACCTGGCTCAGAGGG**	**GluMs-PCR/Bisulfite and TAB sequencing**	***Homo sapiens***	[21,22]
**L1 5´UTR rev (310)**	**GTCAGGGGTCAGGGACCCACTT**	**GluMs-PCR/Bisulfite and TAB sequencing**	***Homo sapiens***	[21,22]

*Homologous recombination template

Supplementary Table 5: Antibodies

Reactivity	Host*	Dilution	Application**	Cat.No. / Clone / ID	Provider / Reference
[redacted]					
α-5mC	Mouse (mAb)	1:250	IF	Clone 32E2	Hybridoma supernatant [31]
α-5hmC	Rabbit (pAb)	1:250	IF	39769	Active Motif, La Hulpe, Belgium
α-5fC	Rabbit (pAb)	1:100	IF	61223	Active Motif, La Hulpe, Belgium
α-5caC	Rabbit (pAb)	1:100	IF	61225	Active Motif, La Hulpe, Belgium
α-PCNA	Mouse (mAb)	1:100	IF	M0879	Agilent Technologies, Inc. / DAKO, Santa Clara, USA
α-TET1	Rat (mAb)	1:10 / 1:2	IF / WB / coIP	Clone 5D8 Clone 4H7	Hybridoma supernatant [24]
α-TET2	Rat (mAb)	1:10 / 1:2	WB	Clone 9F7	Hybridoma supernatant [24]
α-TET3	Rat (mAb)	1:10 / 1:2	WB	Clone 11B6	Hybridoma supernatant [24]
α-MIN	Rat (mAb)	1:200	WB	Clone 1E1	[32]
α-GFP	Rat (mAb)	1:1000	WB	Clone 3H9	Chromotek, Planegg-Martinsried, Germany
GFP binder	nanobody	1 mg/mL	coIP		[33]
α-RFP	Rat (mAb)	1:200	WB	Clone 5F8	[34]
α-LINE-1 ORF1p	Rabbit (mAb)	1:200	IF	ab230966	abcam
α-Cul4	Mouse (mAb)	1:500	WB	Clone H-11, sc-377188	Santa Cruz Biotechnology
α-Cul4B	Rabbit (pAb)	1:1000	WB	HPA058979	Sigma-Aldrich, St Louis, MO, USA
α-VprBP	Rabbit (pAb)	1:1000/1:200	WB / IF	11612-1-AP	ProteinTech, Rosemont, USA
α-Uhrf1	Rabbit (pAb)	1:500/1:200	WB/IF	PA5-29884	Invitrogen, Thermo Fisher Scientific, Waltham, MA, USA
α-hemagglutinin	Mouse (mAb)	1:200	WB	Clone 12CA5	Hybridoma supernatant [35]
α-ubiquitin	Mouse (mAb)	1:500	WB	ST1200	Millipore
α-H3K9me3	Rabbit (mAb)	1:300	IF	39161	Active Motif,La Hulpe, Belgium
[redacted]					
α-rabbit IgG (H+L) Alexa Fluor 488-conjugated	Goat (pAb)	1:500	IF	A-11008	Thermo Fisher Scientific, Waltham, MA, USA
α-mouse IgG (H+L) Alexa Fluor 488-conjugated	Goat (pAb)	1:500	IF	A-11001	Thermo Fisher Scientific, Waltham, MA, USA
α-rabbit IgG (H+L) Alexa Fluor 594-conjugated	Goat (pAb)	1:250	IF	R37117	Thermo Fisher Scientific, Waltham, MA, USA
α-mouse IgG (H+L) Cy5-conjugated	Donkey (pAb)	1:250	IF	715-715-150	The Jackson Laboratory, Bar Harbor, ME, USA
α-rabbit IgG (H+L) Cy3-conjugated	Donkey (pAb)	1:5000	Slot blotting	138876 711-165-152	The Jackson Laboratory, Bar Harbor, ME, USA
α-rat IgG (H+L) Cy3-conjugated	Donkey (pAb)	1:5000	Slot blotting	138876 711-165-153	The Jackson Laboratory, Bar Harbor, ME, USA
α-mouse IgG (H+L) HRP***-conjugated	Sheep (pAb)	1:5000	WB	NA931	GE Healthcare, Chicago, Il, USA
α-rat IgG (H+L) HRP***-conjugated	Goat (pAb)	1:5000	WB	A9037	Sigma-Aldrich, St Louis, MO, USA
α-rabbit IgG (H+L) HRP***-conjugated	Goat (pAb)	1:5000	WB	A0545	Sigma-Aldrich, St Louis, MO, USA

*mAb: monoclonal; pAb: polyclonal; **IF: immunofluorescence, WB: western blot; ***HRP: horseradish peroxidase

Supplementary Table 6: Imaging Systems

Device	Light Sources	Filters (ex & em [nm])*	Objectives/ Lenses	Detection system	Application
**Ultra-View VoX spinning disc on an Inverted Nikon Ti-E microscope; PerkinElmer Life Sciences, UK**	Solid state Diode lasers (405 nm, 488 nm, 561 nm, 640 nm)	405/488/568/640 ** 405: 415–475 488: 505–549 561: 580–650 640: 664–754	Oil immersion 60x Plan-Apochromat (NA 1.49) Oil immersion 100x Plan-Apochromat (NA 1.49)	cooled 14-bit Hamamatsu ® C9100-50 EMCCD	time lapse microscopy & confocal Z-stack imaging
**Leica SP5 II Confocal point scanner**	405 nm Diode Laser 50 mW 488 nm Argon ion laser 458 nm ~5mW 476 nm ~5mW 488 nm ~20mW 496 nm ~5mW 514 nm ~20mW 561 nm DPSS 50 mW 633nm HeNe gas laser 20 mW	DAPI: ex. 420/30 em. 465/20<br><br>FITC: ex. 495/15 em. 530/30<br><br>Rhod: ex. 570/20 em. 640/40<br><br>FITC/Rhod combi filter	HC PL APO 10x / 0.4 CS<br><br>HCX PL APO 40x / 1.3 oil CS<br><br>HCX PL APO 63x / 1.4-0.6 oil lambda blue<br><br>HCX PL APO 100x / 1.44 oil Corr CS	spectral from 400-800 selectivity 0.6-2 nm galvano scanner: up to 1400 Hz resonance scanner: up to 8000 Hz, up to 250 frames per second with 512x512	Time-lapse microscopy & confocal Z-stack imaging<br><br>FRAPs
**Operetta high content screening microscope; PerkinElmer Life Sciences, UK**	Xenon fiber optic Light source, 300 W, 360 – 640 nm continuous spectrum	405: 360-400 & 410–480 488: 460-490 & 500–550 561: 560-580 & 590–640	20x or 40x air (0.45 NA and 0.95 NA) long WD***	14-bit Jenoptik CMOS	high content screening microscopy
**Amersham AI600 Imager; GE Healthcare, Chicago, Il, USA**	UV transillumination light: 312 nm	EtBr: 312 & 585-625	Large aperture f/0.85 FUJINON™	16-bit Peltier cooled Fujifilm Super CCD	EtBr stained gel & HRP stained blot imaging

***ex: excitation & em: emission, ** dichroic specification, *** WD: working distance**

Supplementary Table 7: Software and macros				
**Name**	**Version**	**Website**	**Company/ University**	**Application**
**SerialCloner**	2.6.1	http://serialbasics.free.fr/	Franck Perez (SerialBasics) - 2004-2013	*In silico* cloning
**Volocity**	6.3		PerkinElmer, USA	
**ImageJ**<br>- **Macro 1**<br>- **Macro 2**<br>- **Macro 3**	1.53c	https://imagej.nih.gov/ij/	Wayne Rasband, National Institutes of Health, USA	Image processing and image analysis<br>- Protein accumulation (macro 1)<br>- FRAPs (macro 2)<br>- Quantification of cytosine modifications (macro 3)
**RStudio**	1.1.447 - 1.2.5033	https://rstudio.com/	RStudio	Statistical analysis and plotting
**Nucim**		https://bioimaginggroup.github.io/nucim		Quantitative analyses of the 3D nuclear landscape
**easyFRAPs**	© 2021 easyFRAP -web	https://easyfrap.vmnet.upatras.gr/	Cell Cycle Laboratory, University of Patras, Greece	Analysis of Fluorescence Recovery After Photobleaching (FRAP) data
**Harmony**	3.5.1	https://www.perkinelmer.com/product/harmony-4-8-office-hh17000001	PerkinElmer, USA	High content microscopy imaging and analysis
**SWISS-MODEL homology modelling**		https://swissmodel.expasy.org/	Universität Basel, Biozentrum	Generation of the structural homology model of Tet1s
**UCSF Chimera**		https://www.cgl.ucsf.edu/chimera/	Resource for Biocomputing, Visualization, and Informatics (RBVI) at the University of California	Visualization of Tet1s model
**Clustal Omega**		https://www.ebi.ac.uk/Tools/msa/clustalo/	EMBL-EBI in Hinxton, Cambridge	DNA and protein sequence alignment
**QUMA**		http://quma.cdb.riken.jp/	Quantification tool for methylation analysis, RIKEN Kobe Institute	Bisulfite sequencing analysis
**Adobe Illustrator CS6**		https://www.adobe.com/products/illustrator.html	Adobe	Figure generation

Supplementary References

1. Yaffe, D. & Saxel, O. Serial passaging and differentiation of myogenic cells isolated from dystrophic mouse muscle. *Nature* **270**, 725–727 (1977).
2. Soule, H. D., Vazguez, J., Long, A., Albert, S. & Brennan, M. A human cell line from a pleural effusion derived from a breast carcinoma. *J Natl Cancer Inst* **51**, 1409–1416 (1973).
3. Soule, H. D. *et al.* Isolation and characterization of a spontaneously immortalized human breast epithelial cell line, MCF-10. *Cancer Res.* **50**, 6075–6086 (1990).
4. Lande-Diner, L. *et al.* Role of DNA methylation in stable gene repression. *J. Biol. Chem.* **282**, 12194–12200 (2007).
5. Sharif, J. *et al.* The SRA protein Np95 mediates epigenetic inheritance by recruiting Dnmt1

to methylated DNA. *Nature* **450**, 908–912 (2007).

6. Shcherbakova, D. M. *et al.* Bright monomeric near-infrared fluorescent proteins as tags and biosensors for multiscale imaging. *Nat. Commun.* **7**, 12405 (2016).
7. Sporbert, A., Domaing, P., Leonhardt, H. & Cardoso, M. C. PCNA acts as a stationary loading platform for transiently interacting Okazaki fragment maturation proteins. *Nucleic Acids Res.* **33**, 3521–3528 (2005).
8. Becker, A. *et al.* Poly(ADP-ribosyl)ation of Methyl CpG Binding Domain Protein 2 Regulates Chromatin Structure. *J. Biol. Chem.* **291**, 4873–4881 (2016).
9. Leonhardt, H. *et al.* Dynamics of DNA replication factories in living cells. *J. Cell Biol.* **149**, 271–280 (2000).
10. Rausch, C. *et al.* Cytosine base modifications regulate DNA duplex stability and metabolism. *Nucleic Acids Res.* **49**, 12870–12894 (2021).
11. Easwaran, H. P., Schermelleh, L., Leonhardt, H. & Cardoso, M. C. Replication-independent chromatin loading of Dnmt1 during G2 and M phases. *EMBO Rep.* **5**, 1181–1186 (2004).
12. Schermelleh, L. *et al.* Trapped in action: direct visualization of DNA methyltransferase activity in living cells. *Nat. Methods* **2**, 751–756 (2005).
13. Lindhout, B. I. *et al.* Live cell imaging of repetitive DNA sequences via GFP-tagged polydactyl zinc finger proteins. *Nucleic Acids Res.* **35**, e107 (2007).
14. Casas-Delucchi, C. S. *et al.* Histone acetylation controls the inactive X chromosome replication dynamics. *Nat. Commun.* **2**, 222 (2011).
15. Qin, W., Leonhardt, H. & Spada, F. Usp7 and Uhrf1 control ubiquitination and stability of the maintenance DNA methyltransferase Dnmt1. *J. Cell. Biochem.* **112**, 439–444 (2011).
16. Rottach, A. *et al.* The multi-domain protein Np95 connects DNA methylation and histone modification. *Nucleic Acids Res.* **38**, 1796–1804 (2010).
17. Pichler, G. *et al.* Cooperative DNA and histone binding by Uhrf2 links the two major repressive epigenetic pathways. *J. Cell. Biochem.* **112**, 2585–2593 (2011).
18. De Vos, M. *et al.* Poly(ADP-ribose) polymerase 1 (PARP1) associates with E3 ubiquitin-protein ligase UHRF1 and modulates UHRF1 biological functions. *J. Biol. Chem.*

289, 16223–16238 (2014).

19. Qin, W. *et al.* DNA methylation requires a DNMT1 ubiquitin interacting motif (UIM) and histone ubiquitination. *Cell Res.* **25**, 911–929 (2015).
20. Ludwig, A. K. *et al.* Binding of MBD proteins to DNA blocks Tet1 function thereby modulating transcriptional noise. *Nucleic Acids Res.* **45**, 2438–2457 (2017).
21. Zhang, P. *et al.* L1 retrotransposition is activated by Ten-eleven-translocation protein 1 and repressed by methyl-CpG binding proteins. *Nucleus* **8**, 548–562 (2017).
22. Zhang, P. *et al.* Methyl-CpG binding domain protein 1 regulates localization and activity of Tet1 in a CXXC3 domain-dependent manner. *Nucleic Acids Res.* **45**, 7118–7136 (2017).
23. Frauer, C. *et al.* Different binding properties and function of CXXC zinc finger domains in Dnmt1 and Tet1. *PLoS ONE* **6**, e16627 (2011).
24. Bauer, C. *et al.* Phosphorylation of TET proteins is regulated via O-GlcNAcylation by the O-linked N-acetylglucosamine transferase (OGT). *J. Biol. Chem.* **290**, 4801–4812 (2015).
25. Liu, X. *et al.* UHRF1 targets DNMT1 for DNA methylation through cooperative binding of hemi-methylated DNA and methylated H3K9. *Nat. Commun.* **4**, 1563 (2013).
26. Müller, U., Bauer, C., Siegl, M., Rottach, A. & Leonhardt, H. TET-mediated oxidation of methylcytosine causes TDG or NEIL glycosylase dependent gene reactivation. *Nucleic Acids Res.* **42**, 8592–8604 (2014).
27. Cong, L. *et al.* Multiplex genome engineering using CRISPR/Cas systems. *Science* **339**, 819–823 (2013).
28. Good, C. R. *et al.* A novel isoform of TET1 that lacks a CXXC domain is overexpressed in cancer. *Nucleic Acids Res.* **45**, 8269–8281 (2017).
29. Skene, P. J. *et al.* Neuronal MeCP2 is expressed at near histone-octamer levels and globally alters the chromatin state. *Mol. Cell* **37**, 457–468 (2010).
30. Nicklas, J. A. & Buel, E. Development of an Alu-based, real-time PCR method for quantitation of human DNA in forensic samples. *J. Forensic Sci.* **48**, 936–944 (2003).
31. Weichmann, F. *et al.* Validation strategies for antibodies targeting modified ribonucleotides. *RNA* **26**, 1489–1506 (2020).

32. Mulholland, C. B. *et al.* A modular open platform for systematic functional studies under physiological conditions. *Nucleic Acids Res.* **43**, e112 (2015).
33. Rothbauer, U. *et al.* A versatile nanotrap for biochemical and functional studies with fluorescent fusion proteins. *Mol. Cell. Proteomics* **7**, 282–289 (2008).
34. Rottach, A., Kremmer, E., Nowak, D., Leonhardt, H. & Cardoso, M. C. Generation and characterization of a rat monoclonal antibody specific for multiple red fluorescent proteins. *Hybridoma (Larchmt)* **27**, 337–343 (2008).
35. Wilson, I. A. *et al.* The structure of an antigenic determinant in a protein. *Cell* **37**, 767–778 (1984).
