# Supplementary material for: Isoform-specific and ubiquitination dependent recruitment of Tet1 to replicating heterochromatin modulates methylcytosine oxidation
Source: Nat Commun. 2022 Sep 2;13:5173. doi: 10.1038/s41467-022-32799-8 (PMC9440122; doi:10.1038/s41467-022-32799-8)
Supplement: Supplementary file 2 — Description of Additional Supplementary Files [file 41467_2022_32799_MOESM2_ESM.pdf]

Description of additional supplementary files

Supplementary Movie 1. Time-lapse of C2C12 cells co-transfected with mRFP-PCNA and GFPTet1s imaged live 8 hours post-transfection in 20 minute time intervals. Scale bar = 5 µm.

Supplementary Movie 2. Time-lapse of C2C12 cells co-transfected with mRFP-PCNA and GFPTet1 imaged live 8 hours post transfection in 20 minute time intervals. Scale bar = 5 µm.

Supplementary Data 1. Plot statistics for main and supplementary figures.
n: number of cells of all replicates (if not stated otherwise); StDev: standard deviation; 95% CI: 95
%
confidence interval; p-value: calculated as stated in figure legends and material and methods
section. n.s., not significant, is given for p-values > or equal to 0.05; one star (*) is given for p-valu
es
< 0.05 and > or equal to 0.005; two stars (**) is given for values < 0.005 and > or equal to 0.0005;
three stars (***) is given for values < 0.0005. P-values from independent two-group comparison a
re
shown in the table followed by the numbers assigned to each group in brackets. For example, in
Figure S5D, 0.0002057 is the p-value obtained after the comparison (1-2), 1 GFP and 2 Tet1s.

Supplementary Data 2. Mass spectrometry quantification of DNA modifications in genomic DNA.
Summary of the experimental conditions, the data and analysis is provided in the individual
spreadsheets. Concentrations of DNA modifications were calculated using integrated values from i
on
chromatogram peaks.
