# Supplementary material for: Isoform-specific and ubiquitination dependent recruitment of Tet1 to replicating heterochromatin modulates methylcytosine oxidation
Source: Nat Commun. 2022 Sep 2;13:5173. doi: 10.1038/s41467-022-32799-8 (PMC9440122; doi:10.1038/s41467-022-32799-8)
Supplement: Supplementary file 7 — Reporting Summary [file 41467_2022_32799_MOESM7_ESM.pdf]

nature portfolio

Corresponding author(s): M. Cristina Cardoso

Last updated by author(s): Aug 7, 2022

Reporting Summary

Nature Portfolio wishes to improve the reproducibility of the work that we publish. This form provides structure for consistency and transparency in reporting. For further information on Nature Portfolio policies, see our Editorial Policies and the Editorial Policy Checklist.

Statistics

For all statistical analyses, confirm that the following items are present in the figure legend, table legend, main text, or Methods section.

n/a	Confirmed	
☐	☒	The exact sample size (*n*) for each experimental group/condition, given as a discrete number and unit of measurement
☐	☒	A statement on whether measurements were taken from distinct samples or whether the same sample was measured repeatedly
☐	☒	The statistical test(s) used AND whether they are one- or two-sided<br>*Only common tests should be described solely by name; describe more complex techniques in the Methods section.*
☒	☐	A description of all covariates tested
☒	☐	A description of any assumptions or corrections, such as tests of normality and adjustment for multiple comparisons
☐	☒	A full description of the statistical parameters including central tendency (e.g. means) or other basic estimates (e.g. regression coefficient) AND variation (e.g. standard deviation) or associated estimates of uncertainty (e.g. confidence intervals)
☐	☒	For null hypothesis testing, the test statistic (e.g. *F*, *t*, *r*) with confidence intervals, effect sizes, degrees of freedom and *P* value noted<br>*Give P values as exact values whenever suitable.*
☒	☐	For Bayesian analysis, information on the choice of priors and Markov chain Monte Carlo settings
☒	☐	For hierarchical and complex designs, identification of the appropriate level for tests and full reporting of outcomes
☐	☒	Estimates of effect sizes (e.g. Cohen's *d*, Pearson's *r*), indicating how they were calculated

Our web collection on statistics for biologists contains articles on many of the points above.

Software and code

Policy information about availability of computer code

Data collection	Immunofluorescence and time lapse microscopy data were acquired using the Ultra-View VoX spinning disc microscopy system (Perkin Elmer) powered by the Volocity software (Improvision) version 6.3.<br>High content screening microscopy data were acquired using the Operetta high content screening microscope powered by the Harmony software (PerkinElmer).<br>Gel and blot images were acquired using Amersham Imager 600 imager (GE Healthcare Life Science).
Data analysis	High content screening microscopy image analysis was performed with the Harmony Software (Perkin Elmer) version 3.5.1 and plotted using custom written R scripts (RStudio, versions 1.1.447, V1.2.1335, and V1.2.5033).<br>Gels, blots and immunofluorescence images were analyzed and processed using ImageJ (https://imagej.nih.gov/ij/) version 1.53c.<br>Fluorescence Recovery after Photobleaching experiments were analyzed with EasyFRAPs web (https://easyfrap.vmnet.upatras.gr/)<br>Data was analyzed and plotted with RStudio, versions 1.1.447, V1.2.1335, and V1.2.5033.<br>Images were analyzed using customs written ImageJ macros (https://imagej.nih.gov/ij/) version 1.53c.<br>All custom written R scripts and ImageJ macros are available at https://tudatalib.ulb.tu-darmstadt.de/handle/tudatalib/2873.3, "00_ImageJ_macros.zip" file. Code used in this study for assessment of DAPI intensity classes (Supplementary Fig. 5G) is available under https://bioimaginggroup.github.io/nucim/<br>All figures were generated using Adobe Illustrator CS6.

For manuscripts utilizing custom algorithms or software that are central to the research but not yet described in published literature, software must be made available to editors and reviewers. We strongly encourage code deposition in a community repository (e.g. GitHub). See the Nature Portfolio guidelines for submitting code & software for further information.

Data

Policy information about availability of data

All manuscripts must include a data availability statement. This statement should provide the following information, where applicable:

- Accession codes, unique identifiers, or web links for publicly available datasets
- A description of any restrictions on data availability
- For clinical datasets or third party data, please ensure that the statement adheres to our policy

Raw image files for all figures and all the datasets used in the study are available at https://doi.org/10.48328/tudatalib-594.3

Field-specific reporting

Please select the one below that is the best fit for your research. If you are not sure, read the appropriate sections before making your selection.

☒ Life sciences ☐ Behavioural & social sciences ☐ Ecological, evolutionary & environmental sciences

For a reference copy of the document with all sections, see nature.com/documents/nr-reporting-summary-flat.pdf

Life sciences study design

All studies must disclose on these points even when the disclosure is negative.

Sample size	No statistical methods were used to predetermine sample sizes. Variable sample sizes were chosen depending on the addressed questions and microscopic/evaluation approaches and on the basis of the published literature. Sample size for all datasets are given in Supplementary Data 1.
Data exclusions	For high content microscopy, cells at the border of the images and cells not fitting the set roundness and size parameters were excluded. More details about the exclusion settings are given in the Materials and Methods section.
Replication	All experiments were carried out in independent replicates. The exact number of replicates is given in the Supplementary Data 1. Experiments for microscopic studies were performed at least two times for each condition and confirmed reproducibility of results. For co-immunoprecipitation and western blotting, at least two independent experiments were performed. Each replication was successful.
Randomization	A cell culture with thousands to millions of cells it by itself randomized (e.g. for cell cycle state or overexpression of Tet1s/Tet1-CD). In our case, control and transfected cells used for an experiment were derived from a common cell culture that was split into identical cell culture devices and analyzed in the same way.
Blinding	Since experiments and data analysis were performed by the same investigators, that precluded the possibility of blinding. In addition, the outcome of the experiments was very clear making blinding superfluous.

Reporting for specific materials, systems and methods

We require information from authors about some types of materials, experimental systems and methods used in many studies. Here, indicate whether each material, system or method listed is relevant to your study. If you are not sure if a list item applies to your research, read the appropriate section before selecting a response.

Materials & experimental systems

n/a	Involved in the study
☐	☒ Antibodies
☐	☒ Eukaryotic cell lines
☒	☐ Palaeontology and archaeology
☒	☐ Animals and other organisms
☒	☐ Human research participants
☒	☐ Clinical data
☒	☐ Dual use research of concern

Methods

n/a	Involved in the study
☒	☐ ChIP-seq
☒	☐ Flow cytometry
☒	☐ MRI-based neuroimaging

Antibodies

Antibodies used	Information about antibodies given in this study comprises: host, antibody, dilution (application), clone (if applicable), catalog number, company and is listed in the supplementary tables section (Table 5).<br>- α-5mC, Mouse (mAb), Clone 32E2, Hybridoma supernatant, provided by Helmholtz Zentrum München.<br>- α-5hmC,Rabbit (pAb), Cat.No. 39769, Active Motif, La Hulpe, Belgium.<br>- α-5fC, Rabbit (pAb), Cat.No. 61223, Active Motif, La Hulpe, Belgium.

- α-5caC, Rabbit (pAb), Cat.No. 61225, Active Motif, La Hulpe, Belgium.
- α-PCNA , Mouse (mAb), Cat.No. M0879 Agilent Technologies, Inc. / DAKO, Santa Clara, USA.
- α-TET1 Rat (mAb), Clone 5D8 and Clone 4H7, Hybridoma supernatant, provided by Helmholtz Zentrum München.
- α-TET2 Rat (mAb), Clone 9F7, Hybridoma supernatant, provided by Helmholtz Zentrum München.
- α-TET3 Rat (mAb), Clone 11B6, Hybridoma supernatant, provided by Helmholtz Zentrum München.
- α-MIN Rat (mAb), Clone 1E1, provided by Helmholtz Zentrum München.
- α-GFP Rat (mAb), Clone 3H9, Chromotek, Planegg-Martinsried, Germany.
- GFP binder nanobody (generated and produced by our laboratory in collaboration with Leonhardt laboratory Ludwig Maximilians University, Munich).
- α-RFP Rat (mAb), Clone 5F8, provided by Helmholtz Zentrum München.
- α-LINE-1 ORF1p, Rabbit (mAb), ab230966, abcam.
- α-Cul4, Mouse (mAb), Clone H-11, sc-377188, Santa Cruz Biotechnology.
- α-Cul4B, Rabbit (pAb), Cat.No. HPA058979, Sigma-Aldrich, St Louis, MO, USA.
- α-VprBP, Rabbit (pAb), Cat.No. 11612-1-AP, ProteinTech, Rosemont, USA.
- α-Uhrf1, Rabbit (pAb), Cat.No. PA5-29884, Invitrogen, Thermo Fisher Scientific, Waltham, MA, USA.
- α-hemagglutinin, Mouse (mAb) , Clone 12CA5, Hybridoma supernatant, ECK).
- α-ubiquitin, Mouse (mAb), Cat.No. ST1200, Millipore, provided by BAbCO Berkeley antibody company, Richmond, California, USA.
- α-H3K9me3, Rabbit (mAb), Cat.No. 39161 Active Motif,La Hulpe, Belgium.

Secondary antibodies:
- α-rabbit IgG (H+L) Alexa Fluor 488-conjugated Goat (pAb) Cat.No. A-11008 Thermo Fisher Scientific, Waltham, MA, USA.
- α-mouse IgG (H+L) Alexa Fluor 488-conjugated Goat (pAb) Cat.No. A-11001 Thermo Fisher Scientific, Waltham, MA, USA.
- α-rabbit IgG (H+L) Alexa Fluor 594-conjugated Goat (pAb) Cat.No. R37117 Thermo Fisher Scientific, Waltham, MA, USA.
- α-mouse IgG (H+L) Cy5-conjugated Donkey (pAb) Cat.No. 715-715-150 The Jackson Laboratory, Bar Harbor, ME, USA.
- α-rabbit IgG (H+L) Cy3-conjugated Donkey (pAb) Cat.No. 138876711-165-152, The Jackson Laboratory, Bar Harbor, ME, USA.
- α-rat IgG (H+L) Cy3-conjugated Donkey (pAb) Cat.No. 138876 711-165-153, The Jackson Laboratory, Bar Harbor, ME, USA.
- α-mouse IgG (H+L) HRP-conjugated Sheep (pAb) Cat.No. NA931, GE Healthcare, Chicago, Il, USA.
- α-rat IgG (H+L) HRP-conjugated Goat (pAb) Cat.No. A9037 Sigma-Aldrich, St Louis, MO, USA.
- α-rabbit IgG (H+L) HRP-conjugated Goat (pAb) Cat.No. A0545 Sigma-Aldrich, St Louis, MO, USA.

Validation

All primary antibodies were validated "in-house" for their specificity in immunofluorescence and WB before use.
All secondary antibodies were validated for immunofluorescence and WB by the manufacturer and tested "in-house" for their specificity before use. In addition to that:

- α-5mC, Mouse (mAb), Clone 32E2, For valitation see Weichmann, F. et al. Validation strategies for antibodies targeting modified ribonucleotides. RNA 26, 1489–1506 (2020).
- α-5hmC, Rabbit (pAb), Cat.No. 39769, Active Motif. For validation see https://www.activemotif.com/catalog/details/39769/5-hydroxymethylcytidine-5-hmc-antibody
- α-5fC, Rabbit (pAb), Cat.No. 61223, Active Motif. For validation see https://www.activemotif.com/catalog/details/61223/5-formylcytosine-antibody-5-fc-pab
- α-5caC, Rabbit (pAb), Cat.No. 61225, Active Motif. For validation see https://www.activemotif.com/catalog/details/61225/5-carboxylcytosine-antibody-5-cac-pab
- α-PCNA, Mouse (mAb), Cat.No. M0879 Agilent Technologies. For validation see https://www.sigmaaldrich.com/AU/en/product/mm/mabe288?gclid=Cj0KCQjwuaiXBhCCARIsAKZLt3kHBj-r2hj_9BcUbJYqCnvcXSV_fmQQMTGvpnjwukEckMYJFMtfZwsaAmFTEALw_wcB
- α-TET1 Rat (mAb), Clone 5D8 and Clone 4H7, Hybridoma supernatant. For validation see 24. Bauer, C. et al. Phosphorylation of TET proteins is regulated via O-GlcNAcylation by the O-linked N-acetylglucosamine transferase (OGT). J. Biol. Chem. 290, 4801–4812 (2015).
- α-TET2 Rat (mAb), Clone 9F7, Hybridoma supernatant. For validation see 24. Bauer, C. et al. Phosphorylation of TET proteins is regulated via O-GlcNAcylation by the O-linked N-acetylglucosamine transferase (OGT). J. Biol. Chem. 290, 4801–4812 (2015).
- α-TET3 Rat (mAb), Clone 11B6, Hybridoma supernatant. For validation see 24. Bauer, C. et al. Phosphorylation of TET proteins is regulated via O-GlcNAcylation by the O-linked N-acetylglucosamine transferase (OGT). J. Biol. Chem. 290, 4801–4812 (2015).
- α-MIN Rat (mAb), Clone 1E1. For validation see Mulholland, C. B. et al. A modular open platform for systematic functional studies under physiological conditions. Nucleic Acids Res. 43, e112 (2015).
- α-GFP Rat (mAb), Clone 3H9, Chromotek. For validation see https://www.ptglab.com/products/GFP-antibody-3H9.htm
- GFP binder nanobody
- α-RFP Rat (mAb), Clone 5F8. For validation see Rottach, A., Kremmer, E., Nowak, D., Leonhardt, H. & Cardoso, M. C. Generation and characterization of a rat monoclonal antibody specific for multiple red fluorescent proteins. Hybridoma (Larchmt) 27, 337–343 (2008).
- α-LINE-1 ORF1p, Rabbit (mAb), ab230966, abcam. For validation see https://www.abcam.com/line-1-orf1p-antibody-epr22227-54-ab230966.html
- α-Cul4 Mouse (mAb), Clone H-11, sc-377188, Santa Cruz Biotechnology. For validation see https://www.scbt.com/p/cul-4-antibody-h-11
- α-Cul4B, Rabbit (pAb), Cat.No. HPA058979, Sigma-Aldrich. For validations see https://www.sigmaaldrich.com/AU/en/product/sigma/hpa011880?gclid=Cj0KCQjwuaiXBhCCARIsAKZLt3ncqRuAZCcKcX4x7R9Q4Wsy6qDyj4sDaCcCBaB5IK5aJRpsMoiOOBoaAu7pEALw_wcB
- α-VprBP, Rabbit (pAb), Cat.No. 11612-1-AP, ProteinTech. For validation see https://www.ptglab.com/products/VPRBP-Antibody-11612-1-AP.htm
- α-Uhrf1, Rabbit (pAb), Cat.No. PA5-29884, Invitrogen, Thermo Fisher Scientific. For validation see https://www.thermofisher.com/antibody/product/UHRF1-Antibody-Polyclonal/PA5-29884
- α-hemagglutinin, Mouse (mAb) , Clone 12CA5, Hybridoma supernatant. For validation see Wilson, I. A. et al. The structure of an

antigenic determinant in a protein. Cell 37, 767–778 (1984).
- α-ubiquitin, Mouse (mAb), Cat.No. ST1200, Millipore. For validation see https://www.merckmillipore.com/DE/de/product/Anti-Ubiquitin-Mouse-mAb-FK2,EMD_BIO-ST1200?ReferrerURL=https%3A%2F%2Fwww.google.com%2F
- α-H3K9me3, Rabbit (mAb), Cat.No. 39161 Active Motif. For validation see https://www.activemotif.com/documents/tds/39161.pdf

Secondary antibodies:
- α-rabbit IgG (H+L) Alexa Fluor 488-conjugated Goat (pAb) Cat.No. A-11008 Thermo Fisher Scientific. For validation see https://www.thermofisher.com/antibody/product/Goat-anti-Rabbit-IgG-H-L-Cross-Adsorbed-Secondary-Antibody-Polyclonal/A-11008
- α-mouse IgG (H+L) Alexa Fluor 488-conjugated Goat (pAb) Cat.No. A-11001 Thermo Fisher Scientific. For validation see https://www.thermofisher.com/antibody/product/Goat-anti-Mouse-IgG-H-L-Cross-Adsorbed-Secondary-Antibody-Polyclonal/A-11001
- α-rabbit IgG (H+L) Alexa Fluor 594-conjugated Goat (pAb) Cat.No. R37117 Thermo Fisher Scientific. For validation see https://www.thermofisher.com/antibody/product/Goat-anti-Rabbit-IgG-H-L-Cross-Adsorbed-Secondary-Antibody-Polyclonal/R37117
- α-mouse IgG (H+L) Cy5-conjugated Donkey (pAb) Cat.No. 715-715-150 The Jackson Laboratory. For validation see https://www.jacksonimmuno.com/catalog/products/715-175-150
- α-rabbit IgG (H+L) Cy3-conjugated Donkey (pAb) Cat.No. 138876 711-165-152, The Jackson Laboratory. For validation see https://www.jacksonimmuno.com/catalog/products/711-165-152
- α-rat IgG (H+L) Cy3-conjugated Donkey (pAb) Cat.No. 138876 711-165-153, The Jackson Laboratory. For validation see https://www.jacksonimmuno.com/catalog/products/712-165-153/Donkey-Rat-IgG-HL-Cyanine-Cy3
- α-mouse IgG (H+L) HRP-conjugated Sheep (pAb) Cat.No. NA931, GE Healthcare. For validation see https://www.sigmaaldrich.com/AU/en/product/sigma/gena9311ml
- α-rat IgG (H+L) HRP-conjugated Goat (pAb) Cat.No. A9037 Sigma-Aldrich. For validation see https://www.sigmaaldrich.com/AU/en/product/sigma/a9037
- α-rabbit IgG (H+L) HRP-conjugated Goat (pAb) Cat.No. A0545 Sigma-Aldrich. For validation see https://www.sigmaaldrich.com/AU/en/product/sigma/a0545

Eukaryotic cell lines

Policy information about cell lines

Cell line source(s)	We used the following cell lines:<br>Non-tumorigenic MCF10a (CVCL_0598) and carcinogenic MCF7 (CVCL_0031) human cell lines, obtained from Prof. Dr. Alexander Loewer, Technical University of Darmstadt, Germany.<br>C2C12 mouse myoblast (CVCL_0188), obtained from Bernardo Nadal-Ginard, Harvard Medical School, Boston, USA.<br>Mouse embryonic fibroblast MEF-PM (Dnmt1-/-, p53-/-) and MEF-P (p53-/-), children of CVCL_4240, obtained from Leonhardt laboratory, Ludwig Maximilians University, Munich.<br>Mouse embryonic stem E14 wildtype cells (CVCL_9108) and Uhrf1 deficient cells (Uhrf1-/-), children of CVCL_9108, obtained from Leonhardt laboratory, Ludwig Maximilians University, Munich.<br>HEK293-EBNA human cell line (CVCL_6974), obtained from Invitrogen; Paisley, UK.<br>All the references are given and details are described in Supplementary Table 1.<br>In addition, we describe in this study a series of genetically engineered MCF7 derived lines and their full characterization is shown.
Authentication	For human HEK 293-EBNA, MCF10a and MCF7: Cell Line Authentication Service from ATCC: STR Profiling<br>Mouse cell lines were DAPI counterstained and visually inspected for the presence of chromocenters.<br>Knock-out cells (MEF PM and E14 Uhrf1 deficient cells) were stained with 5mC specific antibody and showed no signal compared to the wild type counterparts. In addition, 5mC was measured in MEF PM by bisulfite sequencing: Casas-Delucchi et al., 2012, NAR, Vol. 40, No. 1 doi:10.1093/nar/gkr723<br>Mouse ES cells were visually inspected for undifferentiated morphology and alkaline phosphatase assay.<br>C2C12 myoblasts were tested for the ability to generate differentiated myotubes and the karyotype was analyzed (Casas-Delucchi et al., 2011, Nature Com. 2:222, DOI: 10.1038/ncomms1218)
Mycoplasma contamination	All cells were used mycoplasma free, as determined by PCR with mycoplasma strains specific primers and by DAPI counterstaining.
Commonly misidentified lines<br>(See ICLAC register)	We used HEK293 cell line derivative for obtaining high level transfection and correspondingly high level of the protein of interest. This was needed for biochemical analysis of protein-protein interactions. Prior to using them, we had the cells validated by STR profiling at ATCC.
